# Evaluation of health benefits of sea lamprey (*Petromyzon marinus*) isolates using *in vitro* antiinflammatory and antioxidant assays

**DOI:** 10.1371/journal.pone.0259587

**Published:** 2021-11-03

**Authors:** Amila A. Dissanayake, C. Michael Wagner, Muraleedharan G. Nair

**Affiliations:** 1 Department of Horticulture, Michigan State University, East Lansing, Michigan, United States of America; 2 Department of Fisheries and Wildlife, Michigan State University, East Lansing, Michigan, United States of America; Tianjin University of Traditional Chinese Medicine, CHINA

## Abstract

Sea lamprey (*Petromyzon marinus*), a parasitic fish which survives on blood of other fishes, is consumed as a delicacy in many countries. Our earlier studies on sea lamprey compounds that showed potential to deter adult sea lampreys yielded several sterols, glycerides, free fatty acids, amino acids, organic acids and nitrogenous compounds. Therefore, this study was to assess the health-benefits of these compounds including additional isolates from HPLC fractions that kept aside due to lack of activity in sea lamprey deterrent assays. *In vitro* cyclooxygenase enzymes (COX-1 and -2) and lipid peroxidation (LPO) inhibitory assays, respectively, were used to determine antiinflammatory and antioxidant activities. Among the tested sterols, cholesteryl eicosapentaenoate and cholesteryl arachidonate exhibited IC_50_ values of 14.6 and 17.7 μg/mL for COX-1 and 17.3 and 20.8 μg/mL for COX-2, respectively. Cholesteryl palmitate and cholesteryl oleate showed moderate COX-1 and COX-2 enzyme inhibition at 25 μg/mL. Amino acids arginine, tyrosine, glutamic acid, tryptophan and asparagine also showed moderate COX-1 and COX-2 inhibition at the same concentration. Among the twelve new isolates from fractions that we did not investigate earlier, a novel uracil derivative petromyzonacil showed COX-1 and COX-2 inhibition at 25 μg/mL by 35 and 15%, respectively. Cholesterol esters tested at 25 μg/mL exhibited LPO inhibition between 38 and 82 percent. Amino acids cysteine, methionine, aspartic acid, threonine, tryptophan, histidine, glutamic acid, phenylalanine and tyrosine at 25 μg/mL showed LPO inhibition between 37 and 58% and petromyzonacil by 32%. These assay results indicate that consumption of sea lamprey offer health-benefits in addition to nutritional benefits.

## Introduction

The sea lamprey (*Petromyzon marinus*) is native to the northeast Atlantic coasts of Norway, Iceland and the Barents Sea, and south along the Atlantic shore to northern Africa [1]. It is an eel-like fish with an anadromous lifestyle in the native range, with a land-locked population in the Laurentian Great Lakes [2]. Historically, it was consumed during lent by the Romans. It is sold in European supermarkets as a highly priced seasonal delicacy and consumed by incorporating it in pies and blood sausages [3,4].

There are many studies on the composition of lipophilic and nitrogenous constituents in muscles and other organs of sea lamprey [5–8]. We have also studied sea lamprey extracts for its adult sea lamprey deterrent activity in field and raceway assays. This resulted in the identification of several lipophilic and nitrogenous compounds from the deterrent fractions of sea lamprey extract [9,10]. Some of the subfractions in the past research, minor in quantities and not deterrent, were kept aside [10]. Since this study was to evaluate the health-benefits of the constituents in the sea lamprey, we isolated and identified pure compounds from those subfractions and conducted bioassays of new and earlier isolates as a block by *in vitro* antiinflammatory and antioxidant activities [11–15].

## Materials and methods

### Isolation and identification of compounds in fractions I-IV

Sea lampreys were collected according to the process approved by the Michigan State University institutional animal care and use committee (permit # AUF 01/14-007-00) [9,10] (see S1 File for experimental details). Experimental details of sea lamprey skin extraction, solvent-solvent partition and MPLC fractionation are provided in the S1 File. Previously, the purification of the water-soluble fraction of the sea lamprey skin extract afforded many subfractions by HPLC. Among these, subfractions **I**-**IV** were not investigated due to lack of sea lamprey deterrent activity in the field assays [10]. However, human health-effects are independent of adult sea lamprey deterrent activity. Therefore, isolation of compounds from all fractions including subfractions (**I**-**IV**) was important to assess the health-beneficial effects of sea lamprey isolates (S1 File).

**Fig 1:**
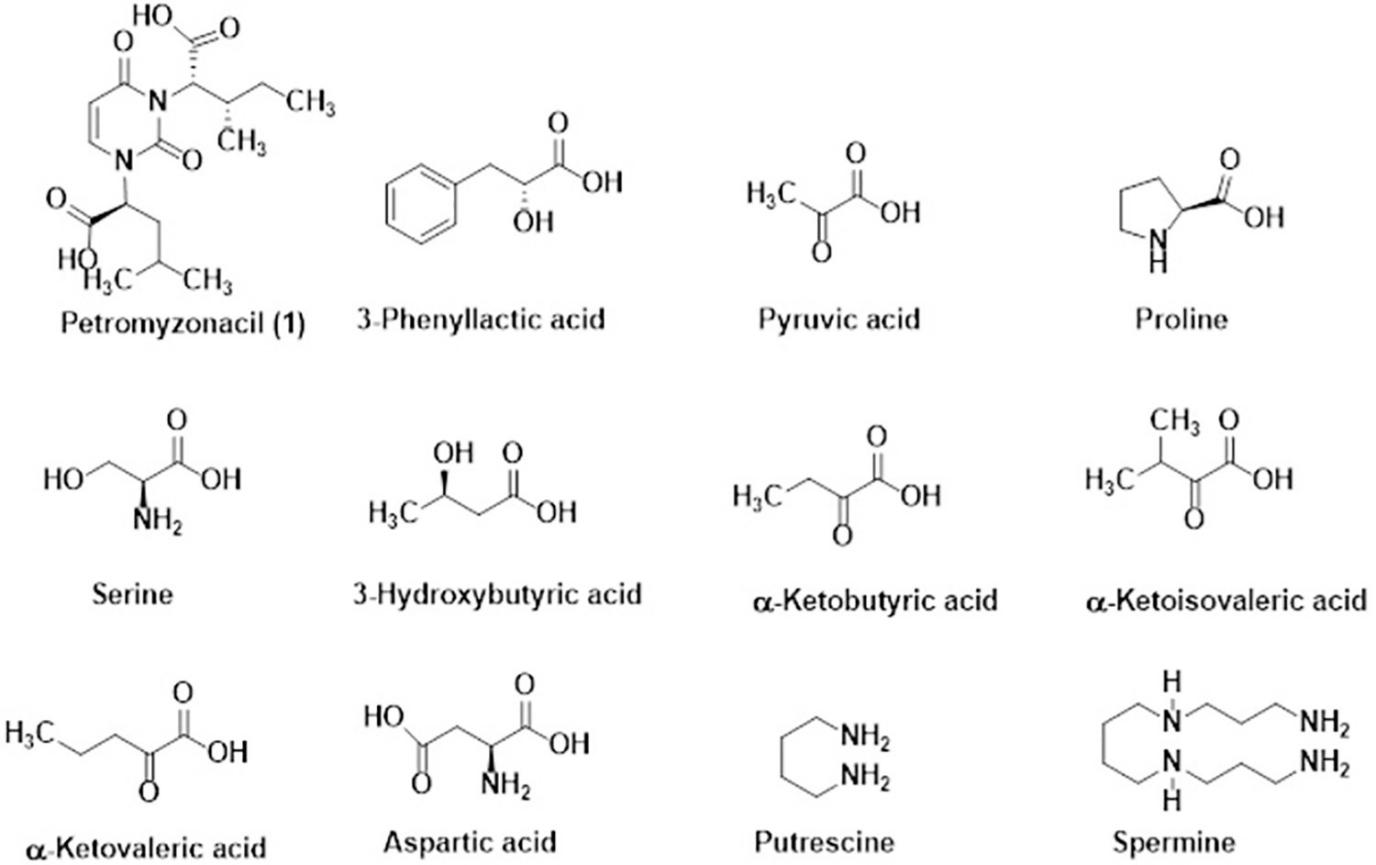
Chemical structures of compounds isolated from subfractions I-IV of the sea lamprey aqueous ethanolic extract: petromyzonacil (1), 3-phenyllactic acid, pyruvic acid, proline, serine, 3-hydroxybutyric acid, α-ketobutyric acid, α-ketoisovaleric acid, α-ketovaleric acid, aspartic acid, putrescine and spermine.

**Fig 2:**
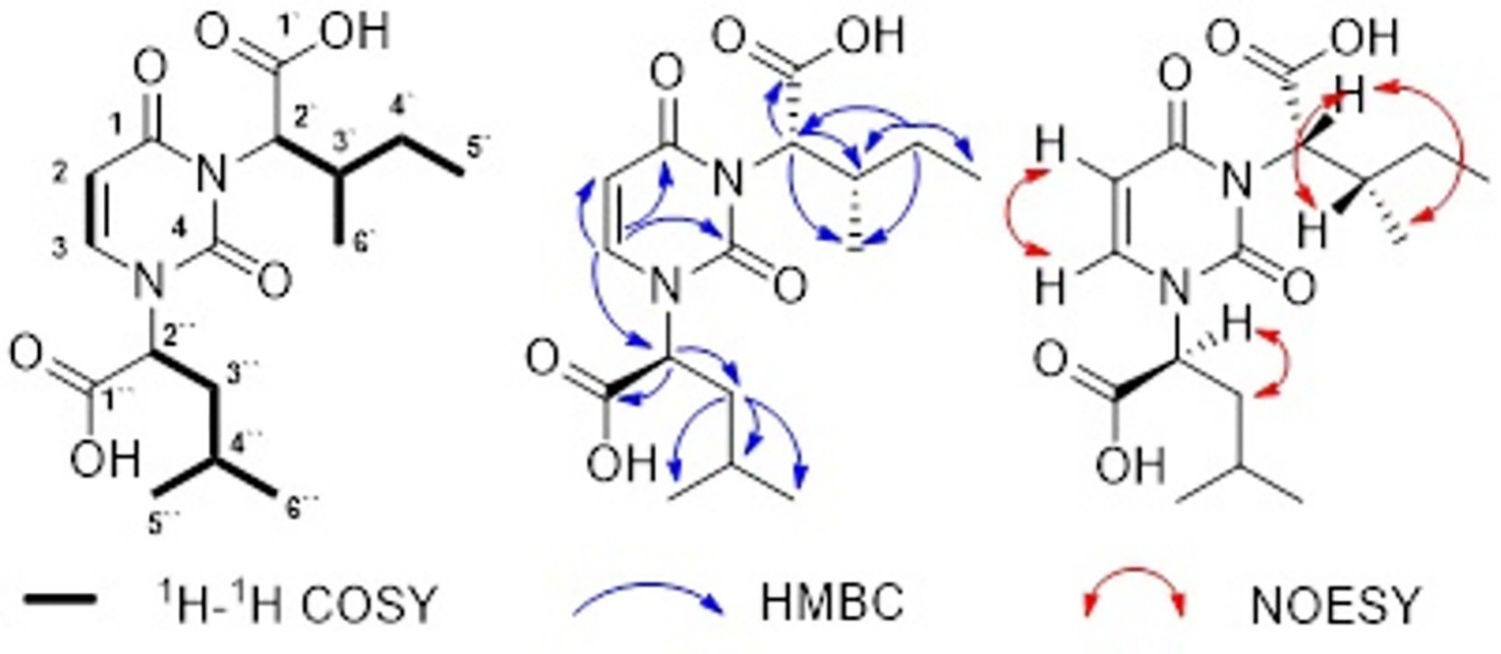
Structure, key 1H-1H COSY (bold lines), HMBC (blue arrows) and NOESY (red arrows) correlations of petromyzonacil (1).

Purification of subfraction **I** (160 mg) was accomplished by HPLC by eluting the column with water:methanol (95:5, *v/v*, 3 mL/min) under isocratic condition. This yielded compound **1** (40 mg, 29.9 min, Figs 1 and 2, Figs A.1-A.14 in S1 File) [16–19], 3-phenyllactic acid (87 mg, 31.4 min, Fig 1, Figs B.1-B5 in S1 File) and pyruvic acid (26 mg, 40.9 min, Fig 1, Figs C.1-C.3 in S1 File), respectively [20]. Similarly, purification of subfraction **II** (150 mg) by HPLC under identical conditions afforded proline (64 mg, 36.9 min, Figs D.1-D.5 in S1 File), serine (19 mg, 39.9 min, Fig 1, Figs E.1-E.4 in S1 File) and 3-hydroxybutyric acid (52 mg, 45.6 min, Fig 1, Figs F.1-F.3 in S1 File), respectively [20].

The subfraction **III** (140 mg) was purified by HPLC by eluting with water:methanol (95:5, *v/v*, 3 mL/min) under isocratic conditions and yielded α-ketobutyric acid (69 mg, 27.7 min, Fig 1, Figs G.1-G.5 in S1 File), α-ketoisovaleric acid (55 mg, 29.9 min, Fig 1, Figs H.1-H.5 in S1 File) and α-ketovaleric acid, respectively (15 mg, 36.9 min, Fig 1, Figs I.1-I.3 in S1 File) [20]. Similarly, purification of subfraction **IV** (225 mg) by HPLC under identical conditions yielded aspartic acid (154 mg, 41.3 min, Fig 1, Figs J.1-J.3 in S1 File), putrescine (37 mg, 45.4 min, Fig 1, Figs K.1-K.5 in S1 File) and spermine (22 mg, 48.4 min, Fig 1, Figs L.1-L.5 in S1 File), respectively [20].

### Cyclooxygenase enzymes (COX) inhibitory assays

The COX-1 and -2 enzyme antiinflammatory assays of pure compounds and positive controls were carried out according to published procedures from our laboratory [11–15,21] (see S1 File for experimental details).

### Lipid peroxidation (LPO) inhibitory assay

Pure compounds and positive controls were assayed for LPO inhibitory activities according to published procedures from our laboratory [11–15,21] (see S1 File for experimental details).

## Results

Fractionation and purification of the uninvestigated fractions earlier from water-soluble fraction of sea lamprey extract used in the sea lamprey deterrent studies were carried out with reverse phase MPLC and preparative HPLC methods [10]. The chemical identity of all pure isolates was determined by ^1^H (500 MHz), ^13^C-NMR (125 MHz) and HRESIMS experiments. The ^1^H and ^13^C chemical shift values were expressed in parts per million (ppm), and residual solvent signals for D_2_O at 3.79 ppm, and for DMSO-d_6_ at 2.50 and 39.9 ppm were used as internal standards, respectively.

New isolates characterized from subfractions **I**-**IV** were compound **1**, 3-phenyllactic acid, pyruvic acid, proline, serine, 3-hydroxybutyric acid, α-ketobutyric acid, α-ketoisovaleric acid, α-ketovaleric acid, aspartic acid, putrescine and spermine. Among these, compound **1** was found to be novel from preliminary spectral evaluations. Therefore, confirmation of its identity in detail using spectral methods is presented below.

Compound **1** was obtained as a white, amorphous powder with [α]^20^_D_ = -3.1 (c = 1.0, H_2_O). The molecular formula was assigned as C_16_H_24_N_2_O_6_, based on the molecular ion at *m/z* 339.1516 [M—H]^-^ (calcd for C_16_H_23_N_2_O_6_, *m/z* 339.1556) in its negative-ion HR-ESITOFMS (Fig A.1 in S1 File). This confirmed six equivalents of unsaturation in the molecule. The UV spectrum showed absorptions for a conjugate carbonyl group (λ_max_ 246 nm), and its IR spectrum showed absorption bands for free carboxylic acids (2925 cm^-1^), carbonyl (1665, 1606, 1573 and 1509 cm^-1^) and aromatic ring (1467 and 1404 cm^-1^) functionalities (Figs A.2 and A.3 in S1 File).

The ^1^H NMR spectrum of compound **1** displayed two set of doublets at δ_H_ 7.35 (H-3, J = 7.6 Hz) and δ_H_ 5.62 (H-2, J = 7.6 Hz) which suggested two coupled olefinic protons in the molecule. It also showed two set of signals at δ_H_ 3.48 (H-2’, d, J = 5.1 Hz) and δ_H_ 3.55 (H-2’’, dd, J = 10.0 and 5.5 Hz) suggested for protons adjacent to free carboxylic acid and imine functionality. In addition, ^1^H NMR displayed signals for two methylene groups at δ_H_ 1.54 (H-3’’, m), δ_H_ 1.29 (H-4a’, ddd, J = 13.5, 7.4 and 4.9 Hz) and δ_H_ 1.08 (H-4b’, ddd, J = 13.5, 7.6 and 4.8 Hz); two methine signals at δ_H_ 1.80 (H-3’, ddd, J = 14.0, 9.7 and 4.8 Hz) and δ_H_ 1.54 (H-4’’, m); four methyl groups at δ_H_ 0.75 (H-5’, dd, J = 7.4 and 7.4 Hz), δ_H_ 0.83 (H-6’, d, J = 7.0 Hz), and δ_H_ 0.79 (H-5’’ and H-6’’, dd, J = 6.0 and 6.0 Hz), respectively (Fig A.4 in S1 File) (Table 1).

**Table 1:** ^1^H and ^13^C NMR spectral data for petromyzonacil (1).

No.	*δ*_H_ (multi, *J* in Hz)	*δ*_C,_ type	HMBC
**1**		167.6 (qC)	
**2**	5.62 (d, *J* = 7.6 Hz, 1H)	100.9 (CH)	3
**3**	7.35 (d, *J* = 7.6 Hz, 1H)	143.6 (CH)	1, 2, 4, 2’’
**4**		153.3 (qC)	
**1’**		174.2 (qC)	
**2’**	3.48 (d, *J* = 4.0 Hz, 1H)	59.4 (CH)	1’, 3’, 4’, 6’
**3’**	1.80 (ddd, *J* = 14.0, 9.7, 4.8 Hz, 1H)	35.8 (CH)	4’, 5’, 6’
**4’a**	1.29 (ddd, *J* = 13.5, 7.4, 4.9 Hz, 1H)	24.3 (CH_2_)	2’, 3’, 5’, 6’
**4’b**	1.08 (ddd, *J* = 13.5, 7.6, 4.8 Hz, 1H)		2’, 3’, 5’, 6’
**5’**	0.75 (dd, *J* = 7.4, 7.4 Hz, 3H)	10.9 (CH_3_)	3’, 4’
**6’**	0.83 (d, *J* = 7.0 Hz, 3H)	14.6 (CH_3_)	2’, 3’, 4’
**1’’**		175.6 (qC)	
**2’’**	3.55 (d, *J* = 5.5 Hz, 1H)	53.3 (CH)	1’’, 3’’, 4’’
**3’’**	1.54 (m, 2H)^a^	39.7 (CH_2_)	2’’, 5’’, 6’’
**4’’**	1.54 (m, 1H) ^a^	24.0 (CH)	2’’, 3’’, 5’’, 6’’
**5’’**	0.79 (dd, *J* = 6.0, 6.0 Hz, 6H)	21.9 (CH_3_)	3’’, 4’’, 6’’
**6’’**		20.7 (CH_3_)	3’’, 4’’, 5’’

^a^ Signals were overlapped.

^b^ HMBC correlations are from proton(s) stated to the indicated carbon.

Carbon-carbon connectivity of compound **1** was elucidated by analysis of HOMODEC and ^1^H-^1^H COSY NMR correlations (Figs A.5-A.7 in S1 File). Selective decoupling of the doublet at δ_H_ 7.35 (H-3) resulted in the collapse of the doublet at δ_H_ 5.62 (H-2) which suggested protons H-2 and H-3 were strongly coupled to each other. Selective decoupling of the doublet at δ_H_ 3.48 (H-2’) resulted in the collapse of the multiplet at δ_H_ 1.80 (H-3’). Similarly, irradiation of the multiplet at δ_H_ 1.80 (H-3’) resulted in the collapse of the doublet at δ_H_ 3.48 (H-2’) to a singlet, multiplets at δ_H_ 1.29 (H-4a’) and δ_H_ 1.08 (H-4b’) to two sets of doublets of doublets and the methyl group doublet δ_H_ 0.83 (H-6’) to a singlet. Furthermore, decoupling of the methyl group doublet at δ_H_ 0.83 (H-6’) changed the multiplet at δ_H_ 1.80 (H-3’) to a quintet (Figs A.5 and A.6 in S1 File). Selective decoupling of the doublet at δ_H_ 3.55 (H-2’’) resulted in the collapse of the multiplet at δ_H_ 1.54 (H-3’’ and H-4’’). Similarly, selective irradiation of the multiplet at δ_H_ 1.54 (H-3’’ and H-4’’) resulted in the collapse the doublet at δ_H_ 3.55 (H-2’’) to a singlet and two methyl doublets resonated at δ_H_ 0.79 (H-5’’ and H-6’’) to a singlet (Figs A.5 and A.6 in S1 File). Analysis of ^1^H-^1^H COSY NMR correlations showed the presence of three isolated proton spin systems and correlations between H-2 to H-3 which supported a vinyl group in compound **1** (Fig 2) (Fig A.7 in S1 File). The ^1^H-^1^H COSY correlations of protons H-2’ to H-3’, H-3’ to H-4’a, H-4’b, H-5’, and H-6’ along with HOMODEC NMR data indicated the presence of a 2-substituted-3-methylpentanoic acid subunit [CH_3_CH_2_CH(CH_3_)CH-COOH] in the molecule (Fig 2) (Figs A.5-A.7 in S1 File). Furthermore, ^1^H-^1^H COSY NMR correlations of protons H-2’’ to H-3’’ and H-4’’ and H-4’’ to H-5’’ and H-6’’ along with HOMODEC NMR data supported a 2-substituted-4-methylpentanoic acid subunit [(CH_3_)_2_CHCH_2_CH-COOH] in the molecule (Fig 2) (Figs A.5-A.7 in S1 File) [16,17].

^13^C NMR along with DEPT NMR indicated four quaternary (δ_C_ 175.6, 174.2, 167.6, 153.3), six methine (δ_C_ 143.6, 100.9, 59.4, 53.3, 38.5, 24.0), two methylene (δ_C_ 39.7, 24.3) and four methyl (δ_C_ 21.9, 20.7, 14.6, 10.9) carbons in compound **1** (Figs A.8 and A.9 in S1 File) (Table 1). The protons H-3 and H-2 were correlated to two olefinic methine carbons at δ_C_ 143.6 (C-3) and δ_C_ 100.9 (C-2), respectively, in its HSQC spectrum (Fig A.10 in S1 File). The HMBC correlations for olefinic methine H-3 to C-1 (δ_C_ 167.6), C-2 (δ_C_ 100.9) and C-4 (δ_C_ 153.3); and olefinic methine H-2 to C-3 (δ_C_ 143.6) combined with ^1^H-^1^H COSY NMR correlations, UV and IR spectral data supported a uracil moiety in compound **1** (Fig A.11 in S1 File). This attributed to four equivalents of unsaturation in the molecule. The protons H-2’, H-3’, H-4’, H-5’, and H-6’ were correlated to C-2’ (δ_C_ 59.4), C-3’ (δ_C_ 35.8), C-4’ (δ_C_ 24.3), C-5’ (δ_C_ 10.9) and C-6’ (δ_C_ 14.6), respectively, in its HSQC spectrum (Fig A.10 in S1 File). The HMBC correlation of methine group at H-2’ to C-1’ (δ_C_ 174.2), C-3’ (δ_C_ 35.8), C-4’ (δ_C_ 24.3), and C-6’ (δ_C_ 14.6); H-3’ to C-4’ (δ_C_ 24.3), C-5’ (δ_C_ 10.9) and C-6’ (δ_C_ 14.6) further supported the 2-substituted-3-methylpentanoic acid subunit in compound **1** (Fig A.11 in S1 File). Furthermore, protons H-2’’, H-3’’, H-4’’, H-5’’, and H-6’’ were correlated to C-2’’ (δ_C_ 53.3), C-3’’ (δ_C_ 39.7), C-4’’ (δ_C_ 24.0), C-5’’ (δ_C_ 21.9) and C-6’’ (δ_C_ 20.7), respectively, in its HSQC spectrum (Figs A.10 and A.11 in S1 File). The HMBC correlation of methine group at H-2’’ to C-1’’ (δ_C_ 175.6), C-3’’ (δ_C_ 39.7) and C-4’’ (δ_C_ 24.0), H-4’’ to C-2’’ (δ_C_ 53.3), C-5’’ (δ_C_ 21.9), and C-6’’ (δ_C_ 20.7) further supported the evidence of a 2-substituted-4-methylpentanoic acid subunit [(CH_3_)_2_CHCH_2_CH-COOH] in compound **1** (Figs A.10 and A.11 in S1 File) [16,17].

Connectivity of the 2-substituted-4-methylpentanoic acid subunit to the uracil ring was deduced by the HMBC correlations of H-3 (δ_H_ 7.35) to C-2’’ (δ_C_ 59.4) (Fig 2) (Fig A.11 in S1 File). The relative configuration of compound **1** was determined by the analysis of the NOESY experiment and spin-spin coupling constants (Fig A.12 in S1 File) (Table 1) [18,19]. The strong NOESY correlations between H-2’ (δ_H_ 3.48)/H-3’ (δ_H_ 1.80) and H-2’ (δ_H_ 3.48)/CH_3_-6’ (δ_H_ 0.83) indicated that the relative configuration of the 2-substituted-3-methylpentanoic acid subunit of compound **1** was as shown in Fig 2 (Fig A.12 in S1 File). This was further supported by the comparison of spin-spin coupling constants between protons H-2’ and H-3’ (^3^J_CH-CH_ 5.1 Hz) and chemical shift values of the H-2’ and H-3’ protons with the similar structural fragments of 2-amino-3-methylpentanoic acid subunits (Fig 2) (Fig A.12 in S1 File) [18,19]. Similarly, relative stereochemistry of the 2-substituted-4-methylpentanoic acid subunit of compound **1** was assigned by the analysis of NOESY correlations between H-2’’ (δ_H_ 3.55)/H-3’’ (δ_H_ 1.55) and H-2’’ (δ_H_ 3.55)/CH_3_-5’’ and 6’’ (δ_H_ 0.79) as shown in Fig 2 (Fig A.12 in S1 File). It was also confirmed by the analysis of spin-spin coupling constants of identical structural fragments of protons H-2’’ and H-3’’ (^3^J_CH-CH_ 10.0 and 5.5 Hz) and chemical shift values of the H-2’’ and H-3’’ protons with the similar uracil structural fragment. Based on all spectral data and analyses, the proposed structure of compound **1** was assigned as a novel uracil analog petromyzonacil (S)-2-(3-((1S,2S)-1-carboxy-2-methylbutyl)-2,4-dioxo-3,4-dihydropyrimidin-1(2H)-yl)-4-methylpentanoic acid (Fig 2) (Figs A.1-A.14 in S1 File) [16–19].

To evaluate the health-beneficial activity of the sea lamprey compounds, we included all isolates of sea lamprey extract for the evaluation of *in vitro* antiinflammatory and antioxidant activities. *In vitro* cyclooxygenase enzyme (COX-1 and -2) inhibitory assays were used to assess antiinflammatory activities of all compounds [11–15] (S1 File). Antiinflammatory activities of glycerides and free fatty acids have been reported by us in other studies and hence the glycerides and fatty acids isolated from sea lamprey were not included in this study [12,14,21]. Commercial NSAIDs ibuprofen, naproxen, Celebrex® and aspirin were used as positive controls and tested at 15, 12, 1, and 108 μg/mL, respectively. The controls showed COX-1 enzyme inhibition by 56, 61, 26 and 56% and COX-2 by 39, 60, 93 and 30%, respectively (Table 2) [11–15,21]. All compounds isolated from sea lamprey were initially tested at 25 μg/mL concentration with results summarized in Table 2 (Figs M-P in S1 File). Cholesteryl palmitate, cholesteryl oleate, cholesteryl eicosapentaenoate and cholesteryl arachidonate showed strong antiinflammatory activity at 25 μg/mL. Therefore, a dose response study was carried out at 6.25, 12.5, 25 and 50 μg/mL concentrations for these compounds as well as petromyzonacil at 12.5, 25, 50, 100 and 200 μg/mL (Figs Q and R in S1 File). Antiinflammatory activities of arginine, tyrosine, tryptophan, asparagine and inosine were assayed at 50 μg/mL concentration. Organic acids α-ketovaleric acid, α-ketoisovaleric acid, α-ketobutyric acid, β-hydroxybutyric acid, pyruvic acid and 3-phenyllactic acid were not active at 25 μg/mL concentration (Table 2).

**Table 2:** Percent antiinflammatory (COX-1 and -2) and antioxidant (LPO) inhibitory activities of compounds isolated from sea lamprey lipophilic and water-soluble fractions tested at 25 μg/mL concentration.

No.	Compound		Bioassay results		
			COX-1^a^	COX-2 ^a^	LPO ^a^
**1**	**Assay controls**	Aspirin	55.9 ± 1.5	29.8 ± 0.9	-
**2**		Celebrex®	25.7 ± 1.9	93.0 ± 1.3	-
**3**		Naproxen	61.4 ± 1.4	60.5 ± 1.3	-
**4**		Ibuprofen	56.4 ± 0.6	38.7 ± 2.0	-
**5**		BHT	-	-	88.0 ± 1.4
**6**		BHA	-	-	88.5 ± 0.7
**7**		TBHQ	-	-	89.5 ± 0.7
**8**	**Sterols**	Cholesteryl palmitate^#^	37.7 ± 2.4	17.7 ± 2.9	38.0 ± 1.4
**9**		Cholesteryl oleate^#^	45.9 ± 0.5	31.1 ± 2.8	59.5 ± 0.7
**10**		Cholesteryl eicosapentaenoate^#^	63.9 ± 2.1	58.5 ± 1.7	72.0 ± 1.4
**11**		Cholesteryl arachidonate^#^	74.7 ± 0.4	67.9 ± 2.2	82.0 ± 1.4
**12**		Cholesterol	2.92 ± 1.4	4.94 ± 2.4	16.5 ± 0.7
**13**	**Lipids**	1,3-Di(cis-9-hexadecenoyl)-2-hexadecanoyl-glycerol	*	*	*
**14**		1,3-Di(cis-9-octadecenoyl)-glycerol	*	*	*
**15**		1,2-Di(cis-9-octadecenoyl)-glycerol	*	*	*
**16**		Free fatty acids	*	*	*
**17**	**Amino acids**	Arginine	29.7 ± 1.3	11.9 ± 1.9	22.0 ± 1.4
**18**		Valine	21.6 ± 0.9	8.33 ± 1.3	18.5 ± 0.7
**19**		Leucine	18.2 ± 1.3	4.89 ± 3.9	19.5 ± 0.7
**20**		Tyrosine	37.2 ± 1.1	21.8 ± 2.4	55.0 ± 1.4
**21**		Isoleucine	23.3 ± 0.6	9.92 ± 0.5	17.5 ± 0.7
**22**		Phenylalanine	15.6 ± 1.7	11.5 ± 1.3	44.0 ± 1.4
**23**		Glutamic acid	32.2 ± 1.9	13.8 ± 2.4	46.0 ± 1.4
**24**		Histidine	21.6 ± 0.4	6.12 ± 0.9	39.5 ± 2.1
**25**		Tryptophan	37.6 ± 0.8	19.6 ± 1.8	57.5 ± 0.7
**26**		Threonine	19.4 ± 1.4	5.82 ± 2.9	45.0 ± 1.4
**27**		Asparagine	26.8 ± 2.3	17.3 ± 0.9	28.0 ± 1.4
**28**		Methionine	15.2 ± 1.5	4.36 ± 0.4	59.0 ± 1.4
**29**		Glycine	12.9 ± 1.4	7.01 ± 0.9	16.5 ± 0.7
**30**		Cysteine	12.9 ± 3.5	9.65 ± 1.3	47.0 ± 1.4
**31**		Proline	21.6 ± 2.4	7.14 ± 1.1	17.0 ± 1.4
**32**		Serine	6.25 ± 2.4	2.91 ± 0.3	29.0 ± 1.4
**33**		Aspartic acid	11.8 ± 0.6	4.23 ± 0.6	36.5 ± 0.7
**34**	**Nitrogenous compounds**	Creatine	13.3 ± 2.7	9.65 ± 1.3	16.5 ± 0.7
**35**		Inosine	35.8 ± 0.5	26.1 ± 0.9	16.5 ± 2.1
**36**		Adenine	11.7 ± 1.8	10.0 ± 1.4	19.5 ± 0.7
**37**		Xanthine	12.7 ± 3.8	4.37 ± 0.6	22.0 ± 1.4
**38**		Hypoxanthine	10.9 ± 1.4	5.82 ± 0.4	24.5 ± 0.7
**39**		Adenosine	16.6 ± 1.1	14.7 ± 0.8	23.5 ± 0.7
**40**		Petromyzonacil (**1**)^#^	25.9 ± 2.2	15.5 ± 1.3	31.5 ± 1.4
**41**		Putrescine	16.9 ± 0.2	10.3 ± 1.4	17.0 ± 1.4
**42**		Spermine	17.5 ± 1.9	3.03 ± 0.5	16.5 ± 0.7
**43**	**Organic acids**	α-Ketovaleric acid	4.13 ± 0.6	4.37 ± 0.5	15.0 ± 1.4
**44**		α-Ketoisovaleric acid	17.1 ± 2.1	10.5 ± 1.8	15.5 ± 0.7
**45**		α-Ketobutyric acid	5.24 ± 0.3	9.65 ± 0.4	16.5 ± 0.7
**46**		β-Hydroxybutyric acid	19.7 ± 1.5	12.3 ± 1.6	21.5 ± 0.7
**47**		Pyruvic acid	9.48 ± 2.9	7.80 ± 0.8	23.5 ± 0.7
**48**		3-Phenyllactic acid	4.42 ± 0.3	11.5 ± 1.6	25.0 ± 1.4

^a^ Positive controls NSAIDs aspirin, Celebrex®, naproxen and ibuprofen were tested for COX enzymes inhibitory assays at 108, 1, 12, and 15 μg/mL, respectively. Standard deviation of each data point (n = 4) and experiments repeated three times. Positive controls BHA, BHT and TBHQ used for LPO assay and tested at 1.80, 2.20 and 1.66 μg/mL. The varying concentrations of positive controls used were to yield a comparable activity profile between 50 and 100%.

* Samples were not assayed since their COX and LPO activities has already been reported from our laboratory.

^#^ For dose-response studies see Figs Q and R in S1 File.

To determine antioxidant activity, lipid peroxidation inhibitory (LPO) assay was used as per earlier publications [11–15,21] (S1 File). As in the case of COX assays, the LPO inhibitory activities of the glycerides and free fatty acids were not assayed in this study since their activities have already been reported from our laboratory [11–15,21]. The positive controls TBHQ, BHA and BHT were tested at 1.66, 1.80 and 2.20 μg/mL. The respective LPO inhibitions obtained for the controls were 89.5, 88.5 and 88% (Table 2) [11–15,21]. Antioxidant activities of compounds from sea lamprey were initially tested at 25 μg/mL concentration and the inhibitory data summarized in Table 2 (Figs S-V in S1 File). Organic acids α-ketovaleric, α-ketoisovaleric, α-ketobutyric, β-hydroxybutyric, pyruvic and 3-phenyllactic acids did not show significant LPO activity at 25 μg/mL test concentration (Table 2).

## Discussion

The aqueous ethanolic Soxhlet extraction of 1 kg of whole adult sea lamprey yielded 83.5 g of lipophilic and 37.6 g of water-soluble constituents. Similarly, separate extraction of 1 kg of skin gave 12.3 g of lipophilic and 10.3 g of water-soluble constituents [9,10]. Previous work by Araujo et al reported about 30–35% of adult sea lamprey body contained dry matter [6]. Further analysis of the dry matter revealed that about 38–51% and 42–54% of it was composed of lipids and proteins, respectively [5–8]. We have reported that the lipophilic fraction of the sea lamprey skin consisted of 18.4% sterols, 34.5% of glycerides and 40.5% of free fatty acids and its lipophilic fraction of the whole sea lamprey is consisted of 4.2% sterols, 57.6% of glycerides and 38.2% of free fatty acids, respectively [9]. The steroidal composition was primarily composed of free cholesterol followed by cholesterol fatty acid esters such as cholesteryl palmitate, cholesteryl oleate, cholesteryl eicosapentaenoate and cholesteryl arachidonate [9]. The glycerides fraction was composed of 90% triglycerides and 10% di-glycerides (1,3 and 1,2 di-substituted glycerides). Based on GCMS analysis, combined free fatty acids and glycerides fractions from sea lamprey skin extract constituted 36.1% saturated, 46.6% monounsaturated (MUFAs) and 17.3% polyunsaturated (PUFAs) fatty acids [9]. Studies revealed that fatty acids and sterols are essential for the body due to their crucial roles in metabolism and signal mediation as well as source of energy [22]. Among the PUFAs we consume ɷ-3, ɷ-6 and ɷ-9 PUFAs plays a vital role in our body and C18:3ɷ3 (α-linolenic acid), C20:5ɷ3 (eicosapentaenoic acid), C22:6ɷ3 (docosahexaenoic acid), C18:2ɷ6 (linoleic acid) and C2O:4ɷ6 (arachidonic acid) are considered as essential fatty acids. In addition, the protective roles of ɷ-3 PUFAs such as docosahexaenoic acid (DHA, C22:6ɷ3) and eicosapentaenoic acid (EPA, C20:5ɷ3)) have been reported in the prevention of many diseases including cancers, inflammatory and autoimmune diseases, cardiovascular diseases as well as psychiatric and mental illnesses [23–25].

Analysis of amino acids composition in liver, muscles and plasma collected from sea lamprey have been reported. Among the free amino acids from liver and the muscles, about 20–25% of it comprised of essential amino acids [5–7]. Our studies showed that the water-soluble fraction contained 19.2% of amino acids, 80.1% of nitrogenous compounds and 0.72% of organic acids [10]. Even though the previous work on liver and muscle tissues and plasma reported amino acids alanine and lysine, we did not detect those in our studies with se lamprey extract [10,11].

Cyclooxygenase enzymes (COX-1 and -2) catalyze the conversion of arachidonic acid to prostaglandin and other signaling intermediates responsible for the onset of inflammation in the body [11–15,21]. Foods with ability to inhibit COX enzymes therefore could relieve the symptoms of inflammation and pain by functioning as modulators of inflammation signaling pathways. The cholesterol esters from sea lamprey showed moderate COX-1 enzyme inhibition by 37–74%, and COX-2 by 17–66% at 25 μg/mL concentration (Table 2) (Fig M in S1 File). As reported earlier, an increase in double bonds in fatty acid chain enhanced the inhibition of COX enzymes and a similar trend in activity was evident in the case of cholesterol esters [21]. Our data indicated that cholesteryl eicosapentaenoate and cholesteryl arachidonate exhibited IC_50_ values of 14.6 and 17.7 μg/mL for COX-1 enzyme and 17.3 and 20.8 μg/mL for COX-2 enzyme, respectively (Fig Q in S1 File). Increasing the concentrations of cholesteryl palmitate and cholesteryl oleate to 50 μg/mL in the assay did not affect the inhibition of COX enzymes. The COX enzymes inhibitory activity profiles of cholesteryl palmitate and cholesteryl oleate were similar to the activity profiles of aspirin and ibuprofen and cholesteryl eicosapentaenoate and cholesteryl arachidonate to that of naproxen.

The water-soluble fraction composed of 19.2% of free amino acids and among them about 50% were essential amino acids [10]. The COX enzyme inhibitory activities of the isolated amino acids at 25 μg/mL showed that arginine, tyrosine, glutamic acid, tryptophan and asparagine inhibited COX-1 enzyme moderately by 30, 37, 32, 37 and 28%, and COX-2 by 12, 22, 14, 20 and 17%, respectively. Increasing the concentration to 50 μg/mL of these amino acids did not impact the inhibitory activities of both COX enzymes. Rest of the amino acid isolates from sea lamprey did not inhibit COX enzymes at 25 μg/mL (Table 2) (Fig N in S1 File). Petromyzonacil and inosine inhibited COX-1 enzyme by 25 and 35%, and COX-2 by 15 and 26%, respectively at 25 μg/mL (Fig O in S1 File). Dose response of petromyzonacil against COX-1 and COX-2 enzymes were carried out at 12.5, 25, 50, 100 and 200 μg/mL concentrations and showed an IC_50_ value of >200 μg/mL (Fig R in S1 File). Other nitrogenous compounds spermine, putrescine, adenosine, hypoxanthine, xanthine, adenine and creatine and organic acids α-ketovaleric, α-ketoisovaleric, α-ketobutyric, β-hydroxybutyric, pyruvic and 3-phenyllactic acids did not inhibit COX enzymes at 25 μg/mL (Table 2) (Figs O and P in S1 File).

The antioxidant assay, the lipid peroxidation inhibitory (LPO) assay, used in this study measures free radical scavenging capability of pure compounds and extracts [11–15,21]. In this assay, peroxidation of the lipid was initiated by the addition of Fe^2+^ resulting in the generation of radicals, which cause the peroxidation of LUV and in turn the fluorescent probes embedded in it. The loss of fluorescent intensity in the assay indicates the oxidative damage to the lipid component of the LUV [11–15,21] (S1 File). At 25 μg/mL concentration, cholesteryl palmitate, cholesteryl oleate, cholesteryl eicosapentaenoate and cholesteryl arachidonate inhibited LPO by 38, 60, 72 and 82%, respectively (Table 2) (Fig S in S1 File). The most abundant steroidal compound in sea lamprey was cholesterol and it lacked LPO inhibitory activity. Antioxidant activity profiles of the cholesterol esters were like its antiinflammatory profiles. That is, an increase in chain length and unsaturation increased the LPO activity. Majority of the lipophilic fraction of the sea lamprey extract consisted of glycerides and free fatty acids [9]. We have reported antioxidant activities of glycerides and free fatty acids and hence did not investigate those compounds again in this study [12,14,21]. Among the amino acids tested at 25 μg/mL, aspartic acid, cysteine, methionine, threonine, tryptophan, histidine, glutamic acid, phenylalanine and tyrosine showed moderate LPO inhibition by 37, 47,59, 45, 58, 40, 46, 44 and 55%, respectively. Other amino acid isolates from sea lamprey did not exhibit significant LPO inhibition at the same concentration (Table 2) (Fig T in S1 File). Petromyzonacil showed moderate LPO inhibition by 32% at 25 μg/mL (Fig U in S1 File). Nitrogenous isolates spermine, putrescine, adenosine, hypoxanthine, xanthine, adenine, inosine and creatine and organic acids (α-ketovaleric, α-ketoisovaleric, α-ketobutyric, β-hydroxybutyric, pyruvic and 3-phenyllactic acid) did not inhibit LPO at 25 μg/mL (Table 2) (Figs U and V in S1 File).

## Conclusions

The whole adult sea lamprey yielded 8.35% of lipophilic and 3.76% of water-soluble extracts and its skin yielded 1.23% of lipophilic and 1.03% of water-soluble extracts, respectively. We have characterized most of the isolable compounds in sea lamprey extract as sterols, glycerides and free fatty acids, and water-soluble extract contained amino acids, organic acids and nitrogenous compounds. Isolation of petromyzonacil and keto acids from adult sea lamprey in this study is for the first time. This is also the first report on the evaluation of *in vitro* antiinflammatory and antioxidant activities of sea lamprey isolates. Among the tested compounds, cholesteryl eicosapentaenoate and cholesteryl arachidonate showed antiinflammatory activity profiles similar to NSAID naproxen at about the same concentration. Our data suggests that the total fatty acids and glycerides content in sea lamprey is similar to anchovy and mackerel. The monounsaturated and polyunsaturated fatty acids (EPA and DHA) in sea lamprey are strong antiinflammatory and antioxidant agents. Bioassays clearly indicated moderate to good antiinflammatory and antioxidant activities for the most compounds present in sea lamprey and the activity profiles were comparable to over the counter or prescription antiinflammatory agents and approved antioxidants employed in food industry. It implies that consumption of sea lamprey provides health-benefits in addition to nutritional benefits like other functional foods. This study also encourages to view sea lamprey as a potential food source rather than just an invasive parasite to fishes and an economic opportunity for the Great Lakes fishing industry.

## Supporting information

S1 File General experimental procedures; NMR, HRMS and HPLC profiles for petromyzonacil (1); NMR data for 3-phenyllactic acid, pyruvic acid, proline, serine, β-hydroxybutyric acid and α-ketobutyric acid, α-ketoisovaleric acid, α-ketovaleric acid, aspartic acid, putrescine and spermine; Antiinflammatory (COX) and antioxidant (LPO) activities of compounds isolated from sea lamprey. (PDF)

## Abbreviations

BHA: Butylated hydroxyanisole
BHT: Butylated hydroxytoluene
COSY: Correlation spectroscopy
COX-1 and 2: cyclooxygenase enzyme-1 and 2
DMSO: Dimethyl sulfoxide
HSQC: Heteronuclear single quantum coherence
HMBC: Heteronuclear multiple bond correlation
HOMODEC: Homonuclear decoupling
HPLC: High pressure liquid chromatography
HR-ESITOFMS: High resolution electron spray ionization time of flight mass spectroscopy
MPLC: Medium pressure liquid chromatography
MUFAs: Monounsaturated fatty acids
NMR: Nuclear magnetic resonance
NOESY: Nuclear overhauser effect spectroscopy
NSAIDs: Non-steroidal antiinflammatory drugs
LPO: Lipid peroxidation
PUFAs: Polyunsaturated fatty acids
TBHQ: Tertiary butylhydroquinone
