# Supplementary material for: Evaluation of health benefits of sea lamprey (Petromyzon marinus) isolates using in vitro antiinflammatory and antioxidant assays
Source: PLoS One. 2021 Nov 3;16(11):e0259587. doi: 10.1371/journal.pone.0259587 (PMC8565778; doi:10.1371/journal.pone.0259587)
Supplement: S1 File — (PDF) [file pone.0259587.s001.pdf]

Evaluation of Health Benefits of Sea Lamprey (*Petromyzon marinus*) Skin Isolates Using *In Vitro* Antiinflammatory and Antioxidant Assays

Amila A. Dissanayake,[1] C. Michael Wagner,[2] Muraleedharan G. Nair,[1*]

[1] Department of Horticulture, Michigan State University, East Lansing, Michigan, United States of America. [2] Department of Fisheries and Wildlife, Michigan State University, East Lansing, Michigan, United States of America

Supplemental Data

S1 File

General experimental procedures

DyChrom C-18 silica gel (60 mesh size, 35−70 µm, Sunnyvale, CA, USA) was used for reverse phase column chromatography. Preparative HPLC (Japan analytical industry Co., Ltd, Tokyo, Japan) separations were conducted on XTerra Prep MS C-8 column (10 µm, 19 x 250 mm, Waters Corporation, Milford, MA, USA). NMR (Nuclear magnetic resonance) spectra were obtained on an Agilent DDR2 500 MHz spectrometer (500 and 125 MHz for ^{1}H and ^{13}C NMR, respectively). The chemical shifts are reported in δ (ppm) and coupling constants (J) are reported in Hz. IR (Infrared) spectra were recorded using a Mattson Galaxy Series FTIR 3000 spectrometer (Mattson Instruments, Inc., Maddison, WI, USA) and UV (ultraviolet) spectra were acquired using a PerkinElmer Lambda35 UV/vis spectrometer (Perkin Elmer Inc., Hebron, KY, USA) [1]. High-resolution mass spectra (HRMS) were acquired on a Waters Xevo G2-S QTOF LC mass spectrometer (Waters Corporation, Milford, MA, USA) spectrometer in negative ion mode. Optical rotations of pure compounds were recorded at the sodium D-line wavelength (589 nm) using a PerkinElmer model 341 polarimeter at 20 °C (PerkinElmer, Waltham, MA, USA) according to published procedure [1]. Arachidonic acid was purchased from Oxford Biomedical Research, Inc. 1-Stearoyl-2-linoleoyl-sn-glycerol-3-phosphocholine (SLPC) was purchased from Avanti Polar Lipids (Alabaster, AL, USA). The fluorescent probe 3-(p-(6-phenyl)-1,3,5-hexatrienyl)phenylpropionic acid was purchased from Molecular Probes (Eugene, OR, USA). All enzymes and reagents were stored in the Bioactive Natural Products and Phytoceuticals Laboratory at Michigan State University (East Lansing, MI, USA).

Collection of migratory sea lamprey for extraction

Migrating sea lamprey were obtained from the annual spring trapping operations of the US Fish and Wildlife Service and the Canadian Department of Fisheries and Oceans in tributaries to Lake Huron (Cheboygan and Ocqueoc Rivers, Michigan, USA). After capture, government staff transported the lampreys to the U.S. Geological Survey's Hammond Bay Biological Station (Millersburg, Michigan, USA; 45.4976906˚N, 84.0363127˚W) and placed them into 800 L tanks receiving continuous water from Lake Huron water (5-18˚C depending on date). Lampreys were held until use in the behavioral assays, or for the collection of skin. Prior to removal of the skin, each animal was euthanized via anesthetic overdose (Ethyl 3-aminobenzoate methanesulfonate aka tricaine methanesulfonate aka MS-222, CAS No. 886-86-2) by immersion in a bath at a concentration of 10 mg L^{-1} until respiration ceased for five minutes, followed by decapitation. After death, the carcass was rinsed in deionized water and the skin removed with a scalpel. Skins were stored at -20˚C until use in the extraction procedures.

Sample collection and extraction

Chopped frozen sea lamprey skins 4.27 Kg (275 skins) were extracted in a Soxhlet apparatus with aqueous ethanolic solvent system (80% ethanol:20% RO water) according to previous reports [1,2]. The combined aqueous ethanolic extract, evaporated under vacuum to remove ethanol, was lyophilized to yield aqueous ethanolic extract as a brown powder (96.7 g). Solvent partitioning of the aqueous ethanolic extract (96.5 g) resulted in chloroform-insoluble (43.9 g) (water-soluble) and chloroform-soluble (52.6 g) fractions. Both fractions were stored at -20 °C until further use.

Isolation and identification of compounds in fractions I-IV

An aliquot (35 g) of the chloroform-insoluble fraction was fractionated on reverse phase silica C-18 MPLC purification system by eluting with isocratic water:methanol (95:5, *v/v*) solvent system gave fractions that were then combined based on similar UV profile. The resulting subfractions were **A** (17.7 g), **B** (7.35 g), **C** (3.76 g), **D** (1.59 g), **E** (1.17 g) and **F** (3.81 g), respectively (S1 file). The HPLC profile of the subfraction **A** showed two peaks and its retention times matched with the HPLC profiles of pure creatine and arginine isolated earlier from the water-soluble fraction [1]. Hence, it was kept aside. Similarly, HPLC profile of the subfraction **B** showed peaks with retention times identical to the HPLC profiles of pure creatine, arginine, valine, leucine and isoleucine isolated earlier from water-soluble fraction and hence kept aside [1].

An aliquot of subfraction **C** (2.81 g) was stirred with methanol (20 mL) to yield methanol soluble (**C-1**, 1.59 g) and methanol insoluble (**C-2**, 1.22 g) subfractions, respectively. HPLC analysis of methanol soluble subfraction **C-1** showed two peaks in its profile and their retention times were identical to the retention times of pure hypoxanthine and inosine isolated earlier from water-soluble fraction and hence kept aside [1]. An aliquot of subfraction **C-2** (960 mg) was fractionated on reverse phase silica C-18 MPLC purification system by eluting with water:methanol (90:10, *v/v*), yielded three subfractions **C-2a** (593 mg), **I** (200 mg), and **II** (161 mg), respectively. HPLC profile of subfraction **C-2a** was identical to the HPLC profile of the subfraction **C-1**. Therefore, subfraction **C-2a** was kept aside. Purification of subfraction **I** (160 mg) was accomplished by HPLC and elution with water:methanol (95:5, *v/v*, 3 mL/min) under isocratic conditions yielded compound **1** (40 mg, 29.9 min), 3-phenyllactic acid (87 mg, 31.4 min) and pyruvic acid (26 mg, 40.9 min), respectively. Similarly, purification of subfraction **II** (150 mg) accomplished by HPLC and elution with water:methanol (95:5, *v/v*, 3 mL/min) under isocratic

conditions yielded proline (64 mg, 36.9 min), serine (19 mg, 39.9 min) and 3-hydroxybutyric acid (52 mg, 45.6 min), respectively.

An aliquot of subfraction **D** was stirred with methanol (15 mL) to yield methanol soluble (**D-1**, 534 mg) and methanol insoluble (**D-2**, 816 mg) subfractions, respectively. Analyses of methanol soluble fraction **D-1** by HPLC showed three peaks in its profile and their retention times were identical to the retention times of pure isolates tyrosine, adenine and xanthine isolated earlier from water-soluble fraction and hence kept aside. An aliquot of subfraction **D-2** (815 mg) was fractionated on reverse phase silica C-18 MPLC purification system by eluting with water:methanol (95:5, *v/v*), yielded three subfractions **D-2a** (442 mg), **III** (140 mg), and **IV** (227 mg), respectively. HPLC profile of sub-fraction **D-2a** was identical to the HPLC profile of the subfraction **D-1**. Therefore, subfraction **D-2a** was kept aside. Purification of subfraction **III** (140 mg) was accomplished by HPLC and elution with water:methanol (95:5, *v/v*, 3 mL/min) under isocratic conditions yielded α-ketobutyric acid (69 mg, 27.7 min), α-ketoisovaleric acid (55 mg, 29.9 min) and α-ketovaleric acid (15 mg, 36.9 min), respectively. Similarly, purification of subfraction **IV** (225 mg) accomplished by HPLC and elution with water:methanol (95:5, *v/v*, 3 mL/min) under isocratic conditions yielded aspartic acid (154 mg, 41.3 min), putrescine (37 mg, 45.4 min) and spermine (22 mg, 48.4 min), respectively.

There were 5 peaks in the HPLC profile of the subfraction **E**. The retention times of all peaks were identical to the retention times of pure isolates histidine, phenylalanine, glutamic acid, tryptophan and threonine isolated and identified earlier from the water-soluble fraction of the sea lamprey skin extract. This fraction, therefore, requires no further investigation and hence kept aside [1]. The subfraction **F** also showed 5 peaks in its HPLC profile. The retention time of these peaks were identical to pure samples of asparagine, methionine, cysteine, adenosine, and glycine isolated

earlier from the water-soluble fraction of the sea lamprey skin extract. This fraction was also kept aside since it requires no further analyses [1].

Cyclooxygenase enzymes (COX) inhibitory assays

COX-1 and -2 enzymes were prepared in our laboratory from ram seminal vesicles (Oxford Biomedical Research, Inc., Oxford, MI, USA) and insect cells cloned with human PGHS-2 enzyme, respectively according to previous reports [2-7]. Inhibitory effects of test samples were measured by monitoring the initial rate of O_2 uptake using an Instech micro-oxygen chamber and electrode (Instech Laboratories, Plymouth Meeting, PA, USA) attached to a YSI model 5300 biological oxygen monitor (Yellow Springs Instrument, Inc., Yellow Springs, OH, USA) at 37 °C following the published procedure [3-8]. The test samples (6 µL) were initially added to the chamber full of assay buffer (Tris 1 mM phenol buffer, 600 µL, pH 7) and hemoglobin (17 µg). COX-1 or COX-2 enzyme (20 µL) was then added and incubated for 2 min. Arachidonic acid (10 µL of solution at 1 mg/mL) was added to initiate the reaction. The data were recorded using Quick Log for Windows data acquisition and control software (Strawberry Tree Inc., Sunnyvale, CA, USA). The positive controls, commercial aspirin, Celebrex, naproxen, and ibuprofen were tested at 108, 1, 15, and 12 µg/mL, respectively. The varying concentrations of pure samples used were to obtain inhibitory activity profiles at 0-100 % range. Each sample was tested in duplicate, and the percent inhibition calculated with respect to DMSO control.

Lipid peroxidation (LPO) inhibitory assay

To determine antioxidant activity, test samples were assayed for LPO inhibition by using large unilamellar vesicles (LUVs) according to procedures established in our laboratory [3-8]. The

positive controls t-butylhydroquinone (TBHQ), butylated hydroxyanisole (BHA) and butylated hydroxytoluene (BHT) were tested at 1.66, 1.80 and 2.20 μg/mL, respectively and pure compounds were tested at 25 μg/mL, respectively. The peroxidation was initiated by the addition of 20 μL of $FeCl_2 \cdot 4H_2O$ (0.5 mM) to the assay mixture [HEPES (100 μL), 1 M NaCl (200 μL), N_2-sparged Millipore water (1.64 mL), DMSO or test sample (20 μL)] and 20 μL of liposome suspension. The fluorescence was monitored at 384 nm on a Turner model 450 fluorometer (Barnstead/ Thermolyne Corp.) at 0, 1, and 3 min and every 3 min thereafter up to 21 min. The decrease in fluorescence intensity over time (21 min) indicated the rate of peroxidation. Each sample was assayed in duplicate, and the percent inhibition was calculated with respect to DMSO control. The varying concentrations of positive controls used were to obtain inhibitory activity profiles at 0-100 % range.

References

1. Dissanayake AA, Wagner MC, Nair MG. Nitrogenous compounds characterized in the deterrent skin extract of migratory adult sea lamprey from the Great Lakes Region. PLOS One. 2019; 14(5):e0217417. https://doi.org/10.1371/journal.pone.0217417.
2. Dissanayake AA, Wagner MC, Nair MG. Chemical characterization of lipophilic constituents in the skin of migratory adult sea lamprey from the Great Lakes Region. PLOS ONE. December 19, 2016. https://doi.org/10.1371/journal.pone.0168609.
3. Dissanayake AA, Ameen BAH, Nair MG. Lipid Peroxidation and Cyclooxygenase Enzyme Inhibitory Compounds from Prangos haussknechtii. Journal of Natural Products. 2017; 80(9):2472–2477. https://doi.org/10.1021/acs.jnatprod.7b00322.
4. Dissanayake AA, Zhang C, Mills GL, Nair MG. Cultivated maitake mushroom demonstrated functional food quality as determined by in vitro bioassays. Journal of Functional Foods. 2018; 44:79-85. https://doi.org/10.1016/j.jff.2018.02.031.
5. Dissanayake AA, Zhang, C, Gabor MAA, Nair MG. Salicylic glycosides in Salix mucronata with antioxidant and anti-inflammatory activities. Natural Product Communications. 2017; 12:1755-1758. https://doi.org/10.1177/1934578X1701201126.
6. Zhang C, Dissanayake AA, Nair MG. Functional food property of honey locust (Gleditsia triacanthos) flowers. Journal of Functional Foods. 2015; 18:266-274. https://doi.org/10.1016/j.jff.2015.07.012.
7. Zhang C, Dissanayake AA, Kevseroğlu K, Nair MG. Evaluation of coriander spice as a functional food by using in vitro bioassays. Food Chemistry. 2015; 167:24-29. https://doi.org/10.1016/j.foodchem.2014.06.120.
8. Henry GE, Momin RA, Nair MG, DeWitt DL. Antioxidant and cyclooxygenase efficacies of fatty acids found in food. J. Agric. Food Chem. 2002; 50:2231-2234. https://doi.org/10.1021/jf0114381.

List of Figures

Figure A.1 Negative-ion HR-ESITOFMS of petromyzonacil (**1**)

Figure A.2 UV/Vis spectrum of petromyzonacil (**1**) in water

Figure A.3 IR spectrum of petromyzonacil (**1**)

Figure A.4 ^{1}H NMR spectrum of petromyzonacil (**1**) in D_2O

Figure A.5 HOMODEC NMR spectrum of petromyzonacil (**1**) in D_2O (δ_H = 7.35, 5.62, 3.55, 3.48, 1.80)

Figure A.6 HOMODEC NMR spectrum of petromyzonacil (**1**) in D_2O (δ_H = 1.54, 1.29, 1.08, 0.75, 0.83)

Figure A.7 ^{1}H-^{1}H COSY NMR spectrum of petromyzonacil (**1**) in D_2O

Figure A.8 ^{13}C NMR spectrum of petromyzonacil (**1**) in D_2O

Figure A.9 DEPT NMR spectrum of petromyzonacil (**1**) in D_2O

Figure A.10 HSQC NMR spectrum of petromyzonacil (**1**) in D_2O

Figure A.11 HMBC NMR spectrum of petromyzonacil (**1**) in D_2O

Figure A.12 NOESY NMR spectrum of petromyzonacil (**1**) in D_2O

Figure A.13 HPLC profile of subfraction of the water-soluble fraction, Solvent system; water: methanol, 98:2 @ 26 °C on a C-8 preparative HPLC column (Xtera, Waters Corp.). Flow rate 3.0 mL/min and peaks monitored at 210 nm

Figure A.14 HPLC profile of pure petromyzonacil (**1**), Solvent system; water: methanol, 98:2 @ 26 °C on a C-8 preparative HPLC column (Xtera, Waters Corp.). Flow rate 3.0 mL/min and peaks monitored at 210 nm

Figure B.1 ^{1}H NMR spectrum of 3-phenyllactic acid in DMSO

Figure B.2 ^{13}C NMR spectrum of 3-phenyllactic acid in DMSO

Figure B.3 HSQC NMR spectrum of 3-phenyllactic acid in DMSO

Figure B.4 HMBC NMR spectrum of 3-phenyllactic acid in DMSO

Figure B.5 ^{1}H-^{1}H COSY NMR spectrum of 3-phenyllactic acid in DMSO

Figure C.1 ^{1}H NMR spectrum of pyruvic acid in D_2O

Figure C.2 ^{13}C NMR spectrum of pyruvic acid in D_2O

Figure C.3 DEPT NMR spectrum of pyruvic acid in D_2O

Figure D.1 ^{1}H NMR spectrum of proline in D_2O

Figure D.2 ^{13}C NMR spectrum of proline in D_2O

Figure D.3 HSQC NMR spectrum of proline in D_2O

Figure D.4 HMBC NMR spectrum of proline in D_2O

Figure D.5 ^{1}H-^{1}H COSY NMR spectrum of proline in D_2O

Figure E.1 ^{1}H NMR spectrum of serine in D_2O

Figure E.2 ^{13}C NMR spectrum of serine in D_2O

Figure E.3 HSQC NMR spectrum of serine in D_2O

Figure E.4 HMBC NMR spectrum of serine in D_2O

Figure F.1 ^{1}H NMR spectrum of 3-hydroxybutyric acid in D_2O

Figure F.2 ^{13}C NMR spectrum of 3-hydroxybutyric acid in D_2O

Figure F.3 DEPT NMR spectrum of 3-hydroxybutyric acid in D_2O

Figure G.1 ^{1}H NMR spectrum of α-ketobutyric acid in D_2O

Figure G.2 ^{13}C NMR spectrum of α-ketobutyric acid in D_2O

Figure G.3 HSQC NMR spectrum of α-ketobutyric acid in D_2O

Figure G.4 HMBC NMR spectrum of α-ketobutyric acid in D_2O

Figure G.5 ^{1}H-^{1}H COSY NMR spectrum of α-ketobutyric acid in D_2O

Figure H.1 ^{1}H NMR spectrum of α-ketoisovaleric acid in D_2O

Figure H.2 ^{13}C NMR spectrum of α-ketoisovaleric acid in D_2O

Figure H.3 HSQC NMR spectrum of α-ketoisovaleric acid in D_2O

Figure H.4 HMBC NMR spectrum of α-ketoisovaleric acid in D_2O

Figure H.5 ^1H-^1H COSY NMR spectrum of α-ketoisovaleric acid in D_2O

Figure I.1 ^1H NMR spectrum of α-ketovaleric acid in D_2O

Figure I.2 ^{13}C NMR spectrum of α-ketovaleric acid in D_2O

Figure I.3 DEPT NMR spectrum of α-ketovaleric acid in D_2O

Figure J.1 ^1H NMR spectrum of aspartic acid in D_2O

Figure J.2 ^{13}C NMR spectrum of aspartic acid in D_2O

Figure J.3 HMBC NMR spectrum of aspartic acid in D_2O

Figure K.1 ^1H NMR spectrum of putrescine in D_2O

Figure K.2 ^{13}C NMR spectrum of putrescine in D_2O

Figure K.3 HSQC NMR spectrum of putrescine in D_2O

Figure K.4 HMBC NMR spectrum of putrescine in D_2O

Figure K.5 ^1H-^1H COSY NMR spectrum of putrescine in D_2O

Figure L.1 ^1H NMR spectrum of spermine in D_2O

Figure L.2 ^{13}C NMR spectrum of spermine in D_2O

Figure L.3 HSQC NMR spectrum of spermine in D_2O

Figure L.4 HMBC NMR spectrum of spermine in D_2O

Figure L.5 ^1H-^1H COSY NMR spectrum of spermine in D_2O

Figure M COX enzyme inhibitory activity of NSAIDs and sterols

Figure N COX enzyme inhibitory activity of NSAIDs and amino acids

Figure O COX enzyme inhibitory activity of NSAIDs and nitrogenous compounds

Figure P COX enzyme inhibitory activity of NSAIDs and organic acids

Figure Q Dose-response curves for the inhibition COX enzymes by cholesterol esters

Figure R Dose-response curves for the inhibition COX enzymes by petromyzonacil (**1**)

Figure S Antioxidant activities of positive controls and cholesterol esters

Figure T Antioxidant activities of positive controls and amino acids

Figure U Antioxidant activities of positive controls and nitrogenous compounds

Figure V Antioxidant activities of positive controls and organic acids

Figure A.1 Negative-ion HR-ESITOFMS of petromyzonacil (**1**)

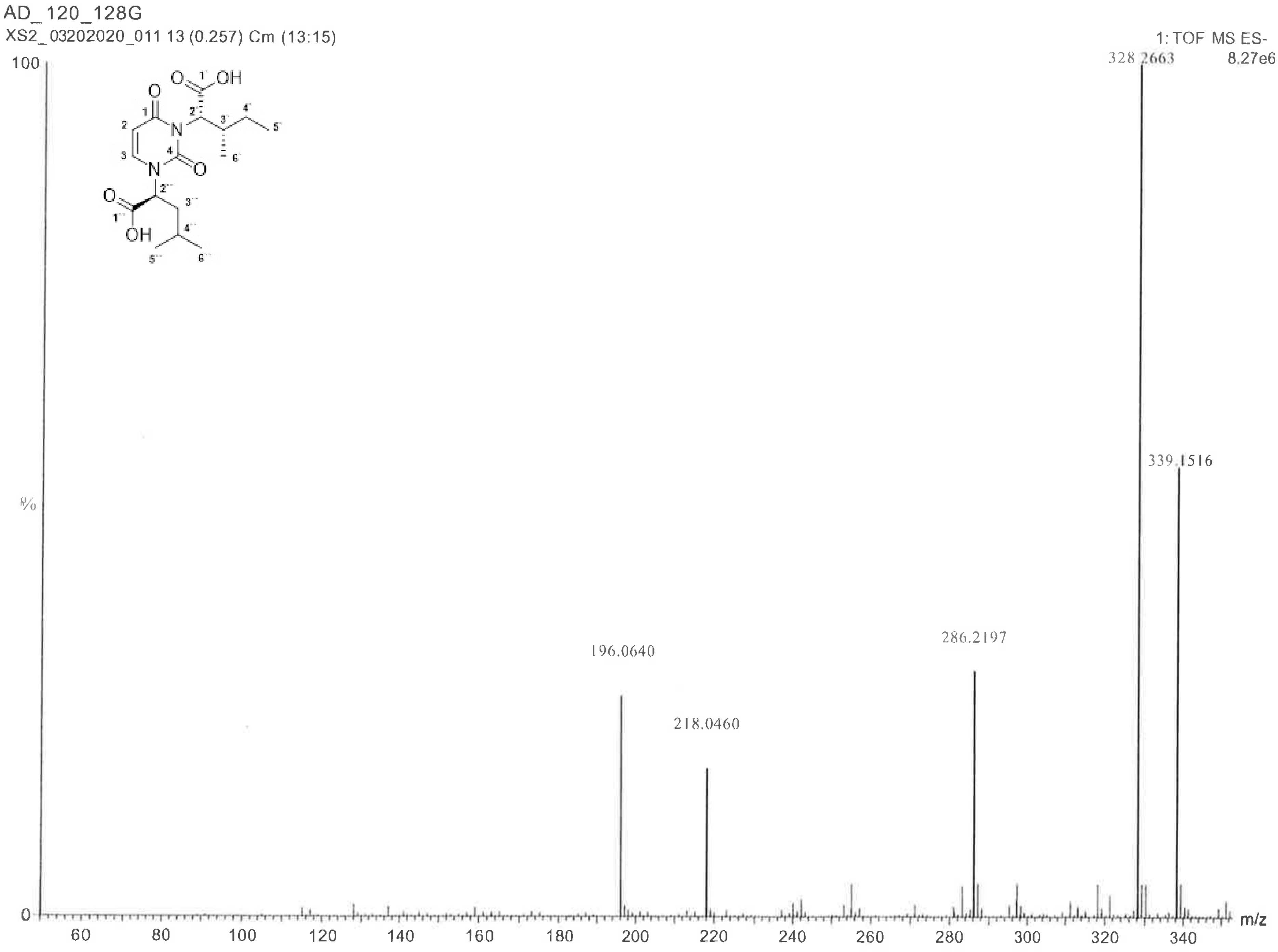

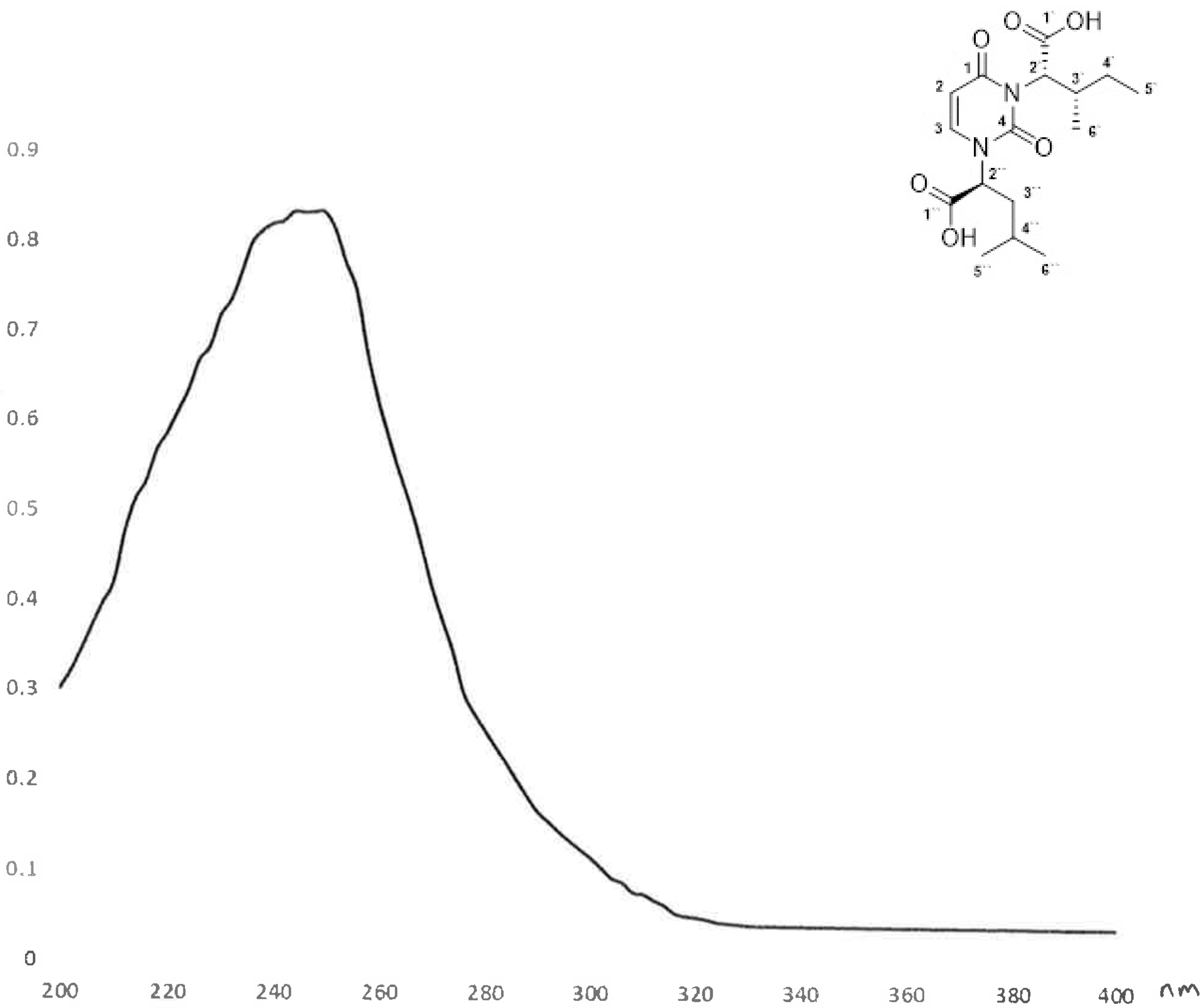

Figure A.2 UV/Vis spectrum of petromyzonacil (**1**) in water

Figure A.3 IR spectrum of petromyzonacil (**1**)

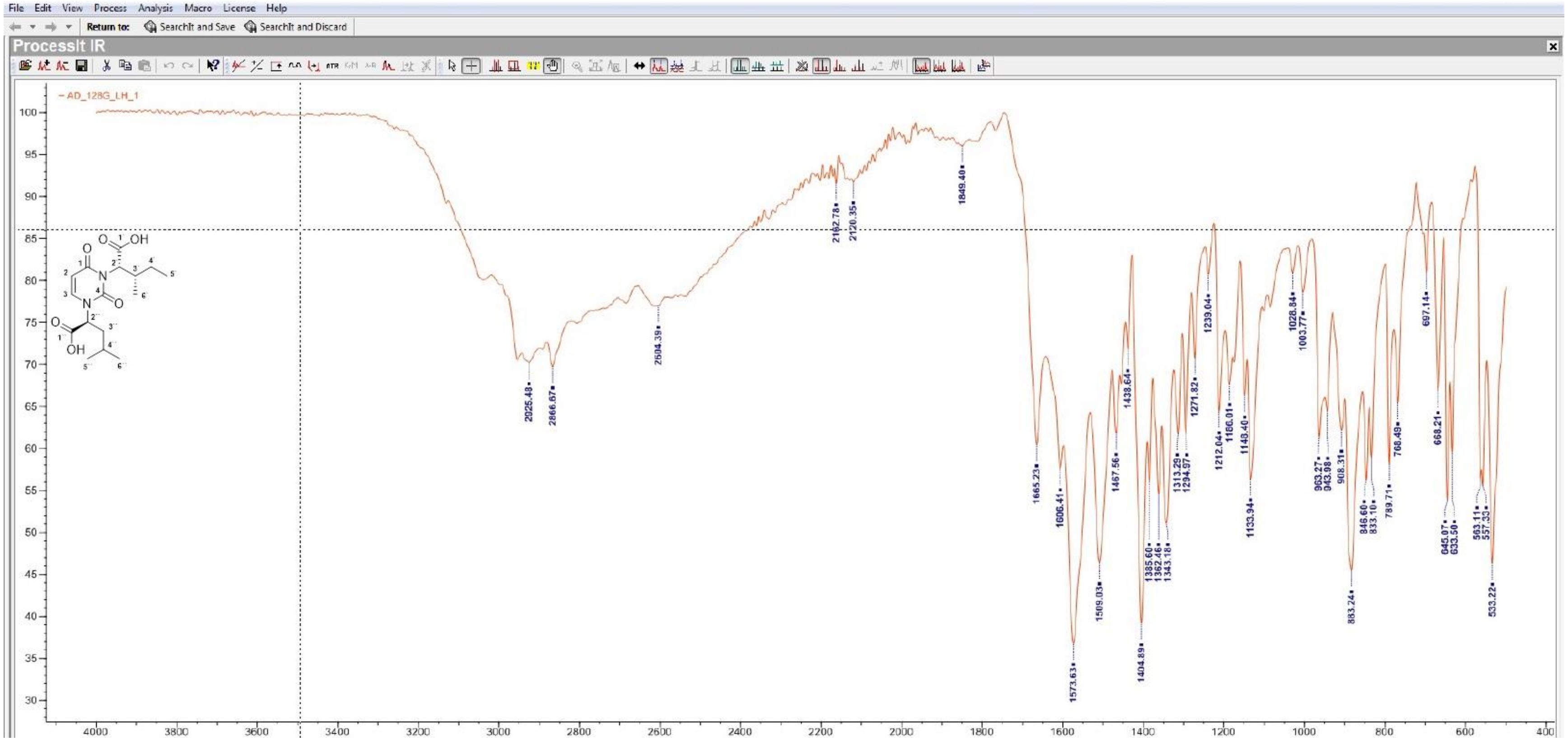

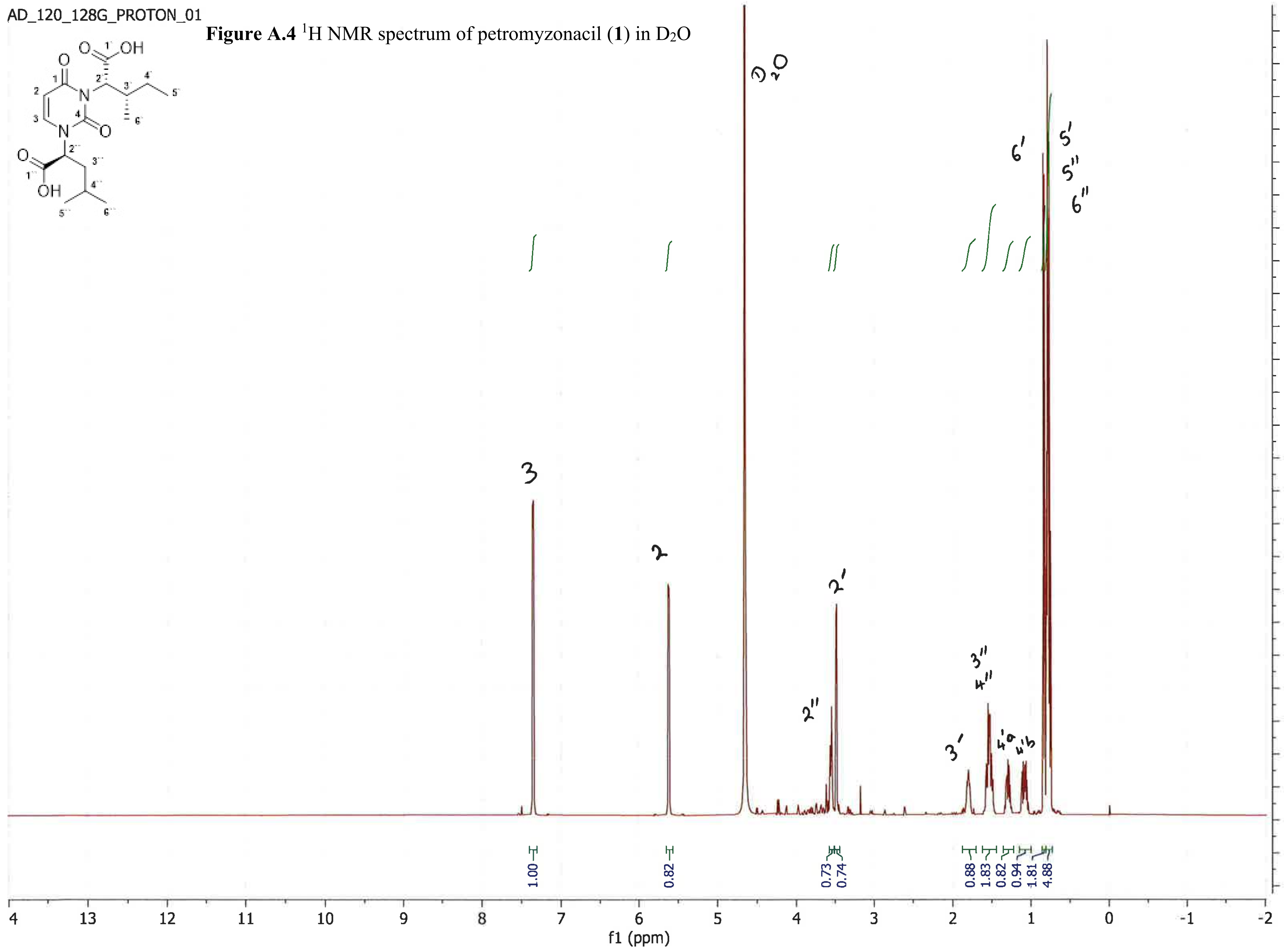

Figure A.4 ^{1}H NMR spectrum of petromyzonacil (**1**) in D_2O

Figure A.5 HOMODEC NMR spectrum of petromyzonacil (**1**) in D_2O (δ_H = 7.35, 5.62, 3.55, 3.48, 1.80)

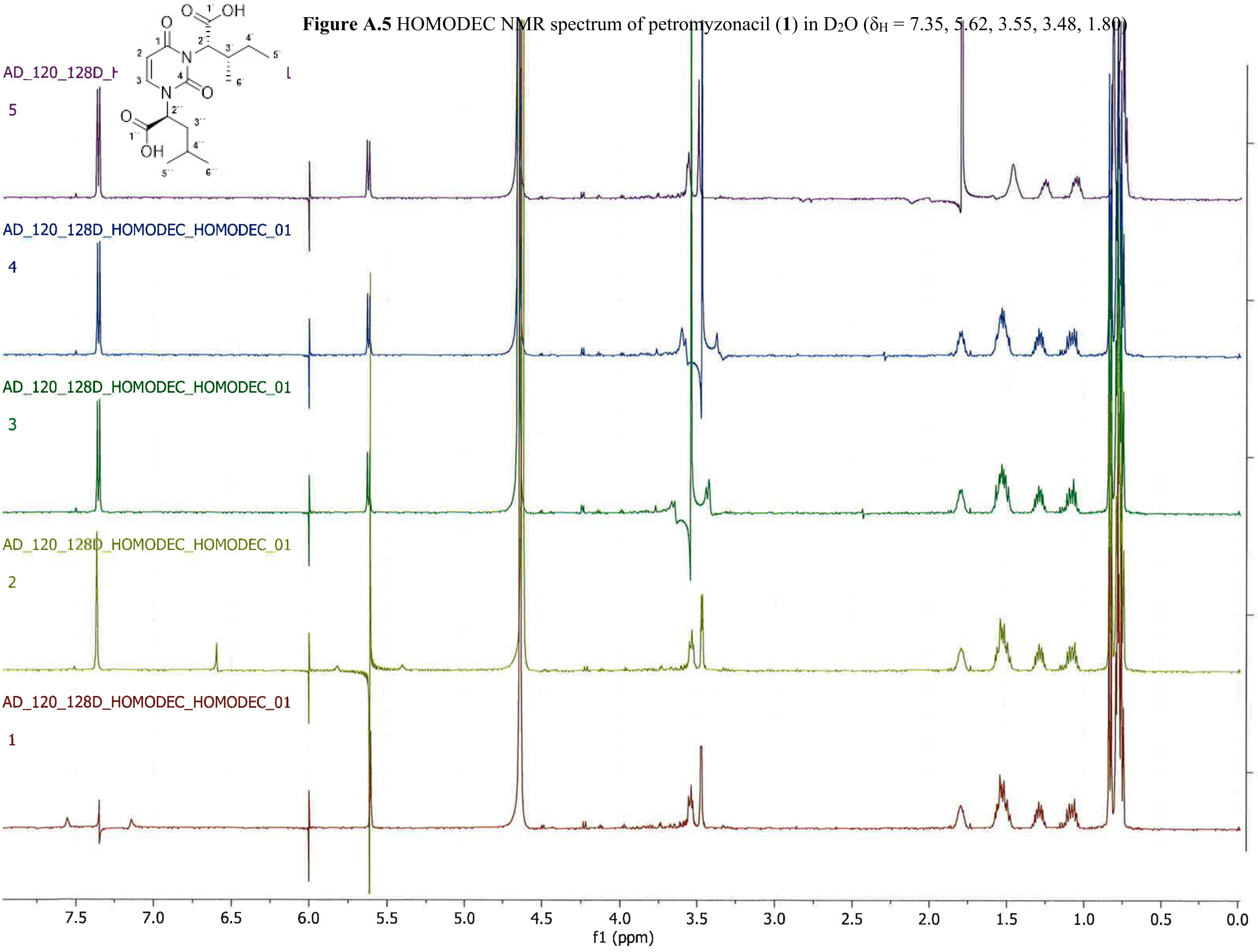

Figure A.6 HOMODEC NMR spectrum of petromyzonacil (**1**) in D_2O (δ_H = 1.54, 1.29, 1.08, 0.75, 0.83)

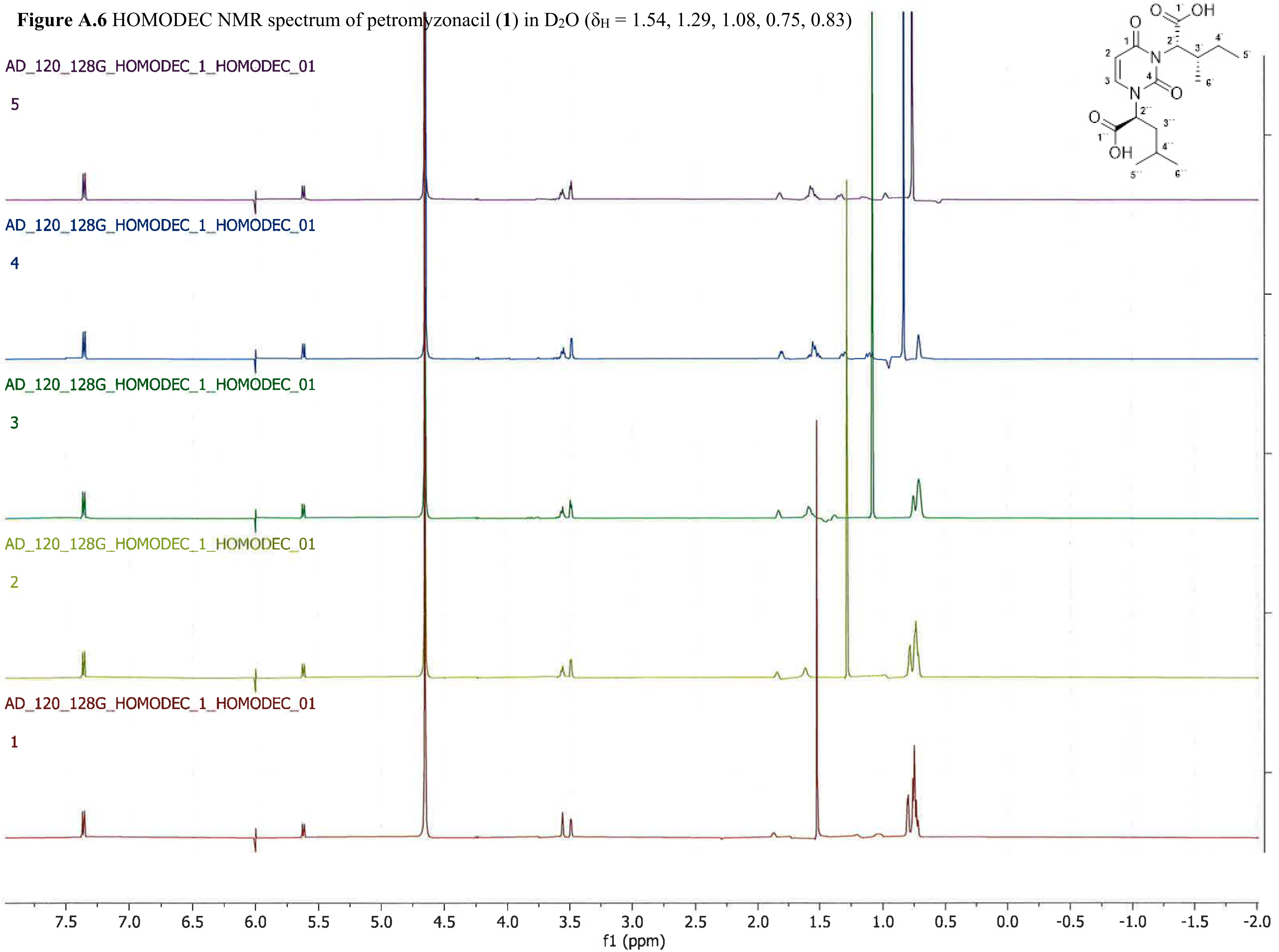

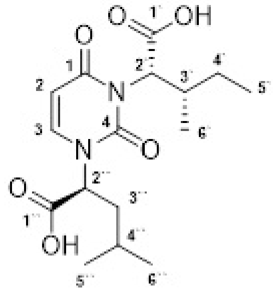

Figure A.7 ^{1}H-^{1}H COSY NMR spectrum of petromyzonacil (**1**) in D_2O

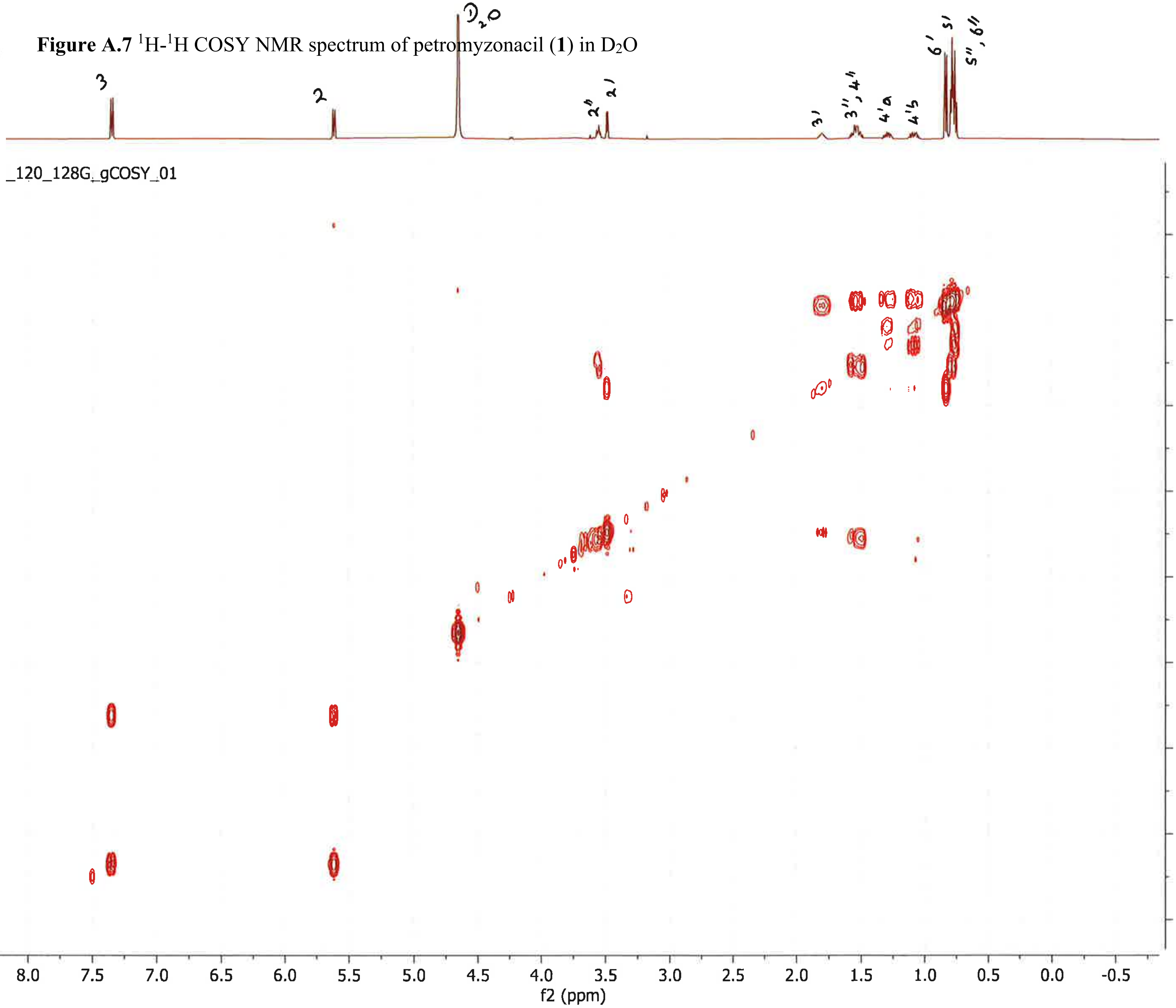

AD_120_128G_CARBON_01

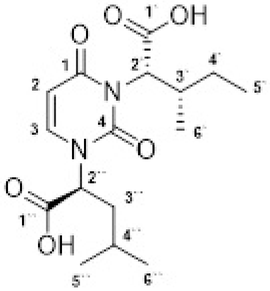

Figure A.8 ^{13}C NMR spectrum of petromyzonacil (**1**) in D_2O

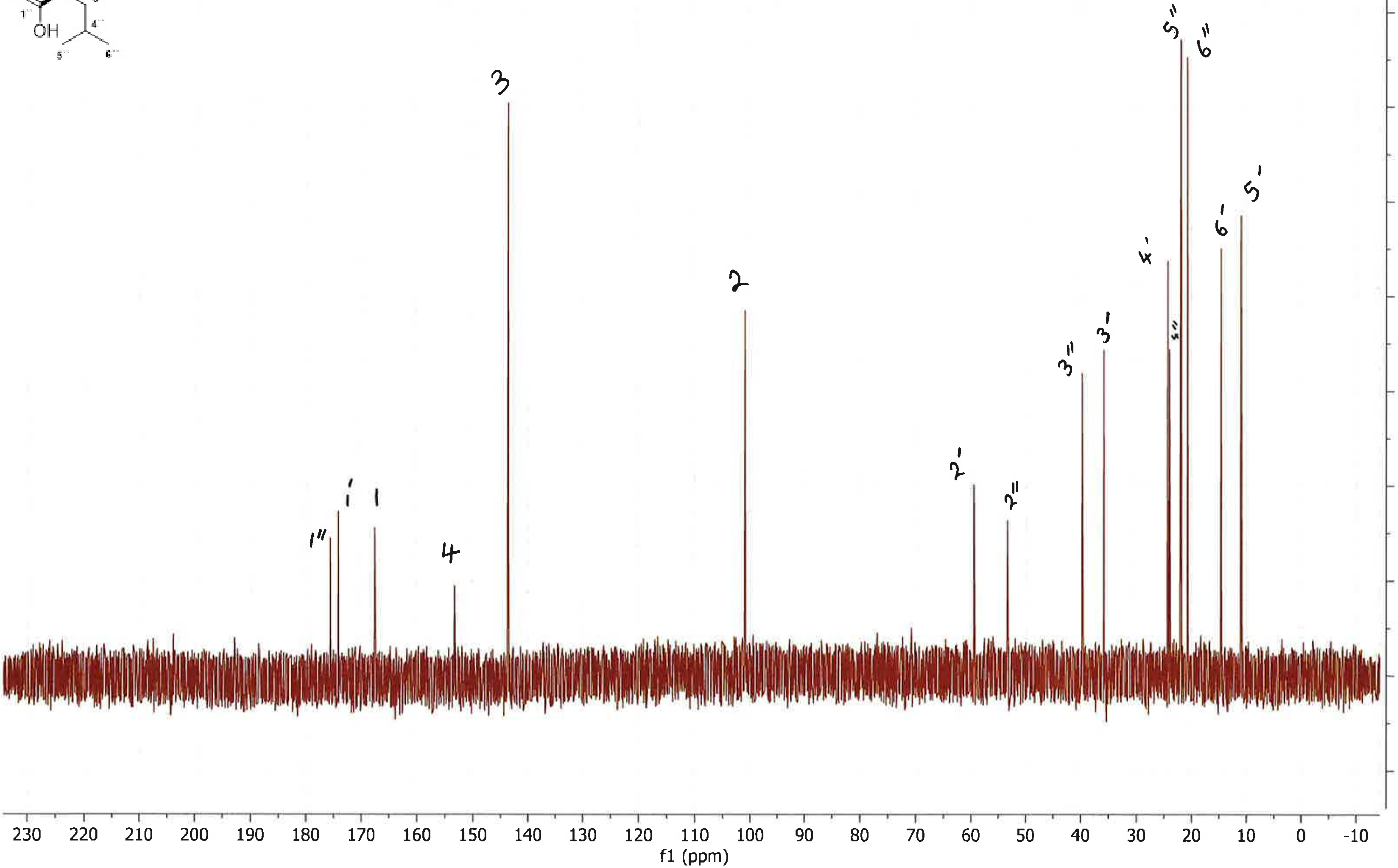

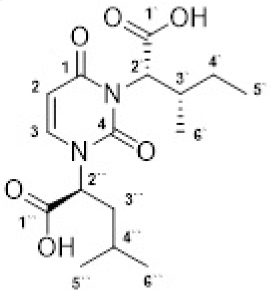

Figure A.9 DEPT NMR spectrum of petromyzonacil (**1**) in D_2O

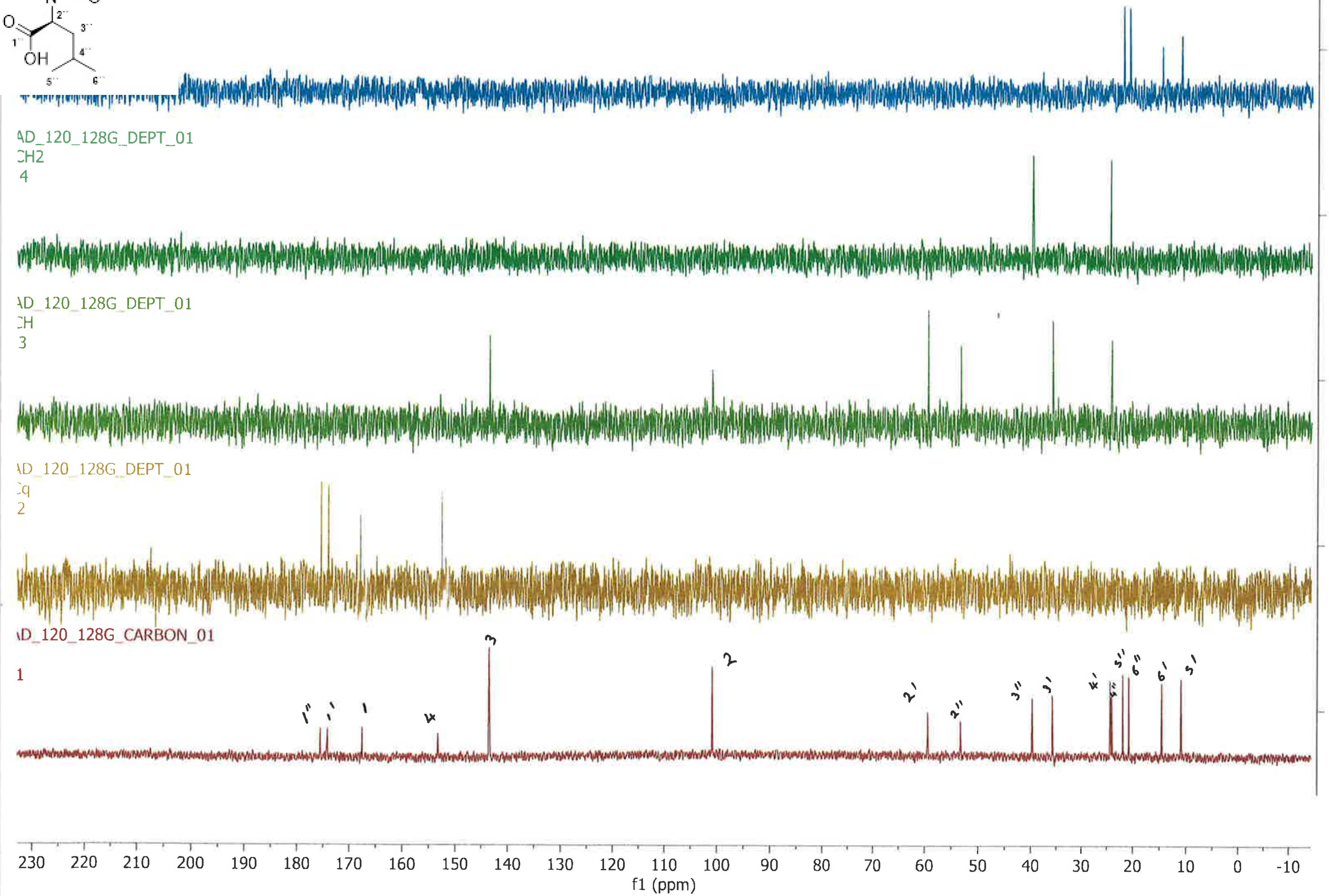

Figure A.10 HSQC NMR spectrum of petromyzonacil (**1**) in D_2O

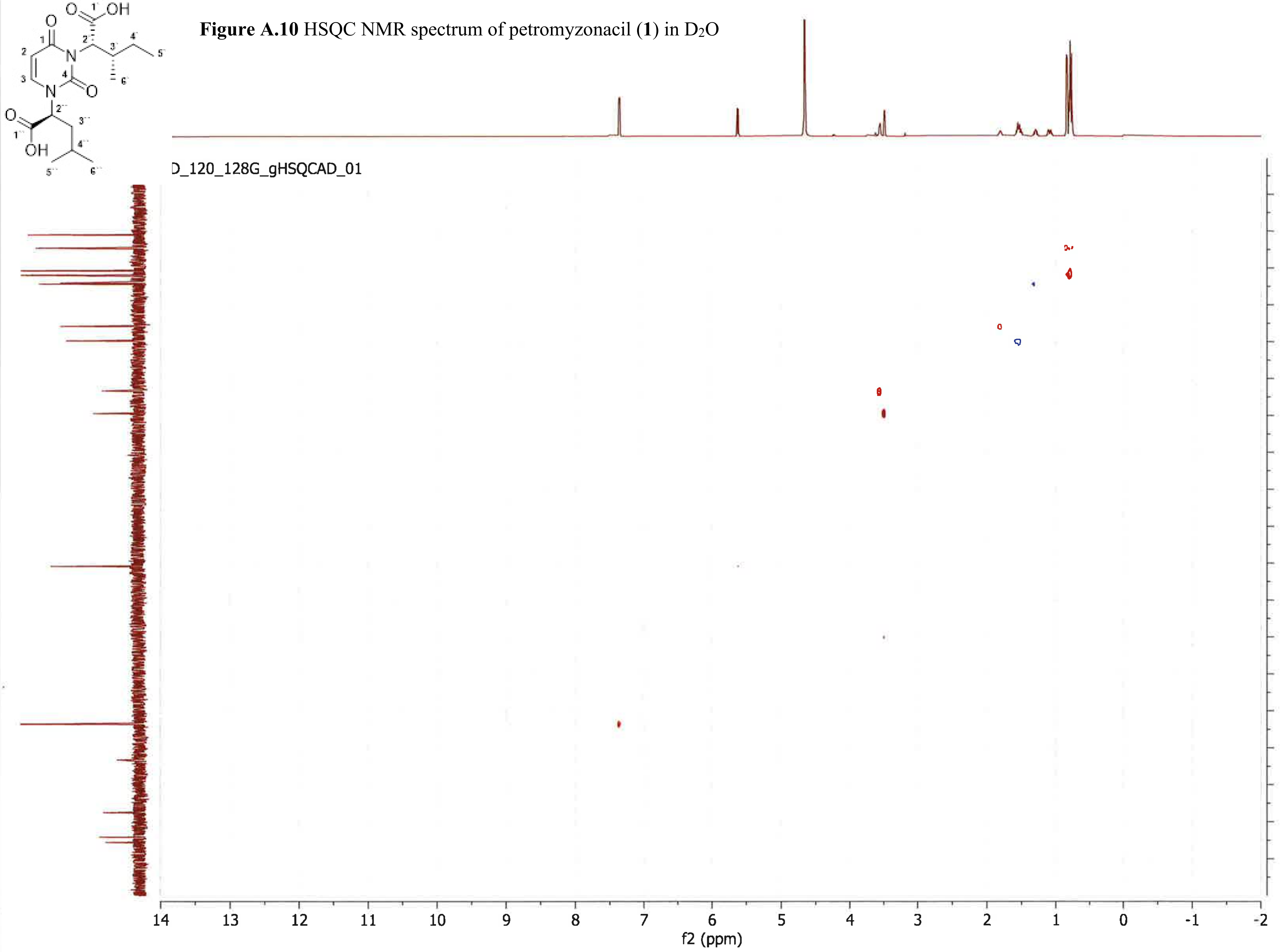

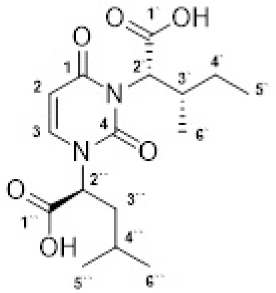

Figure A.11 HMBC NMR spectrum of petromyzonacil (**1**) in D_2O

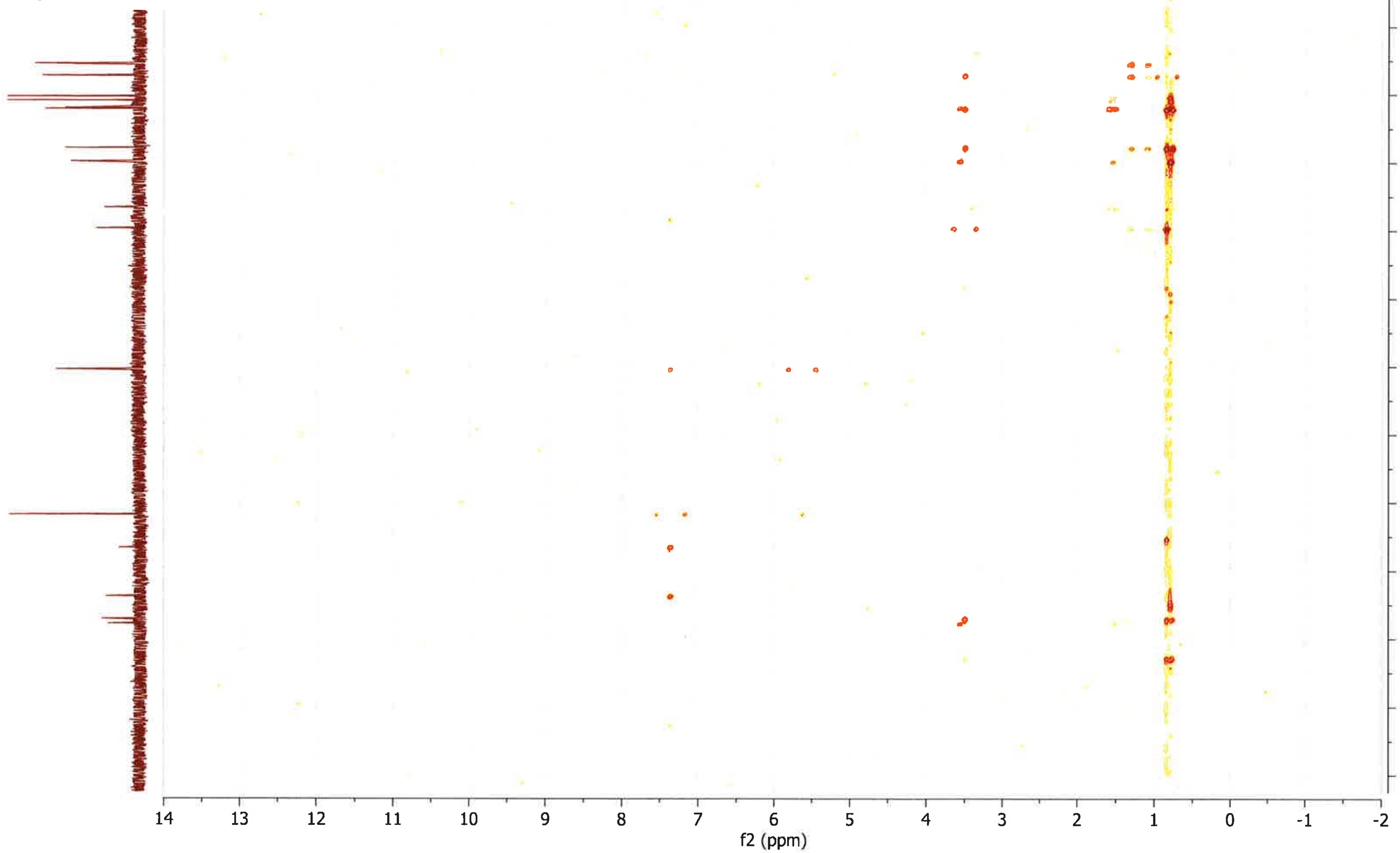

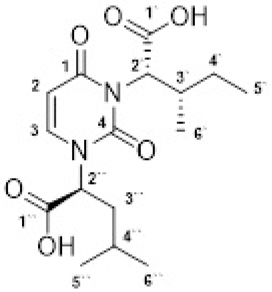

Figure A.12 NOESY NMR spectrum of petromyzonacil (**1**) in D_2O

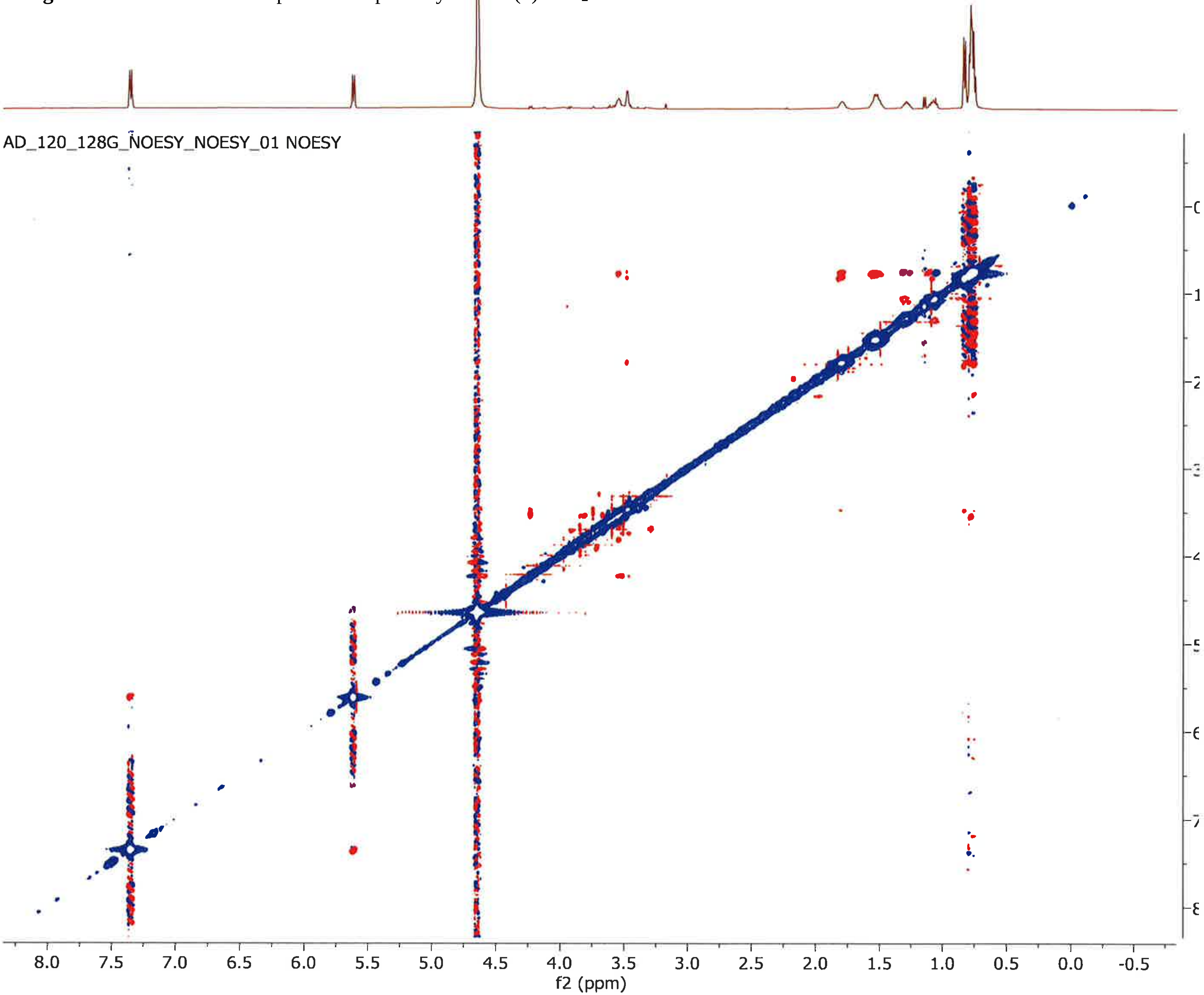

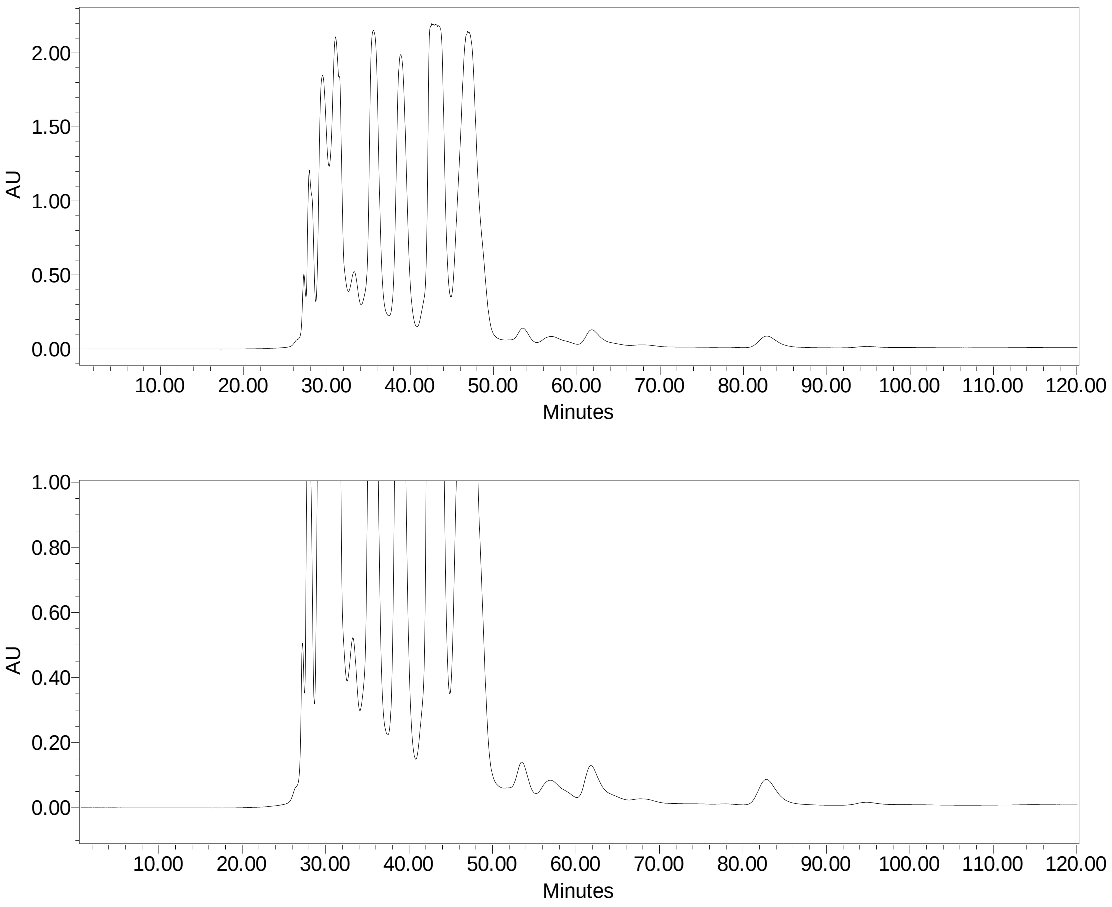

Figure A.13 HPLC profile of subfraction of the water-soluble fraction, Solvent system; water: methanol, 98:2 @ 26 °C on a C-8 preparative HPLC column (Xtera, Waters Corp.). Flow rate 3.0 mL/min and peaks monitored at 210 nm

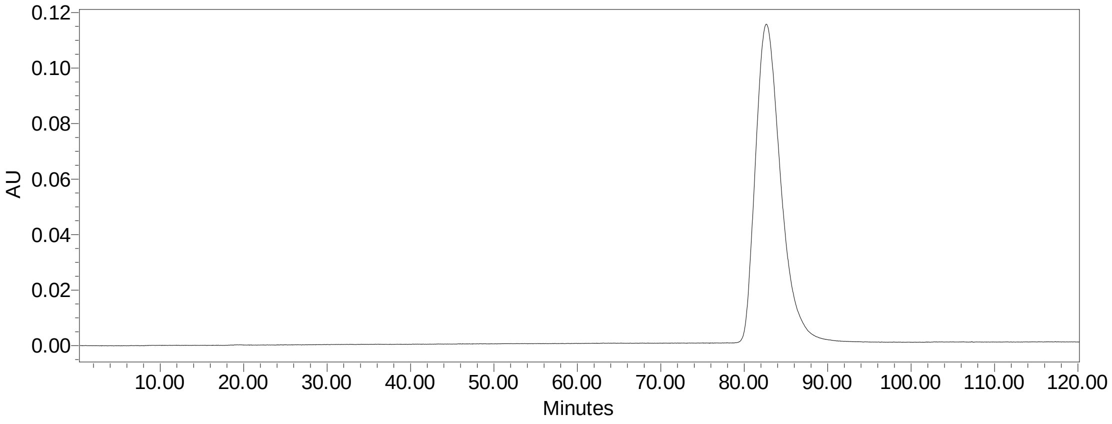

Figure A.14 HPLC profile of pure petromyzonacil (**1**), Solvent system; water: methanol, 98:2 @ 26 °C on a C-8 preparative HPLC column (Xtera, Waters Corp.). Flow rate 3.0 mL/min and peaks monitored at 210 nm

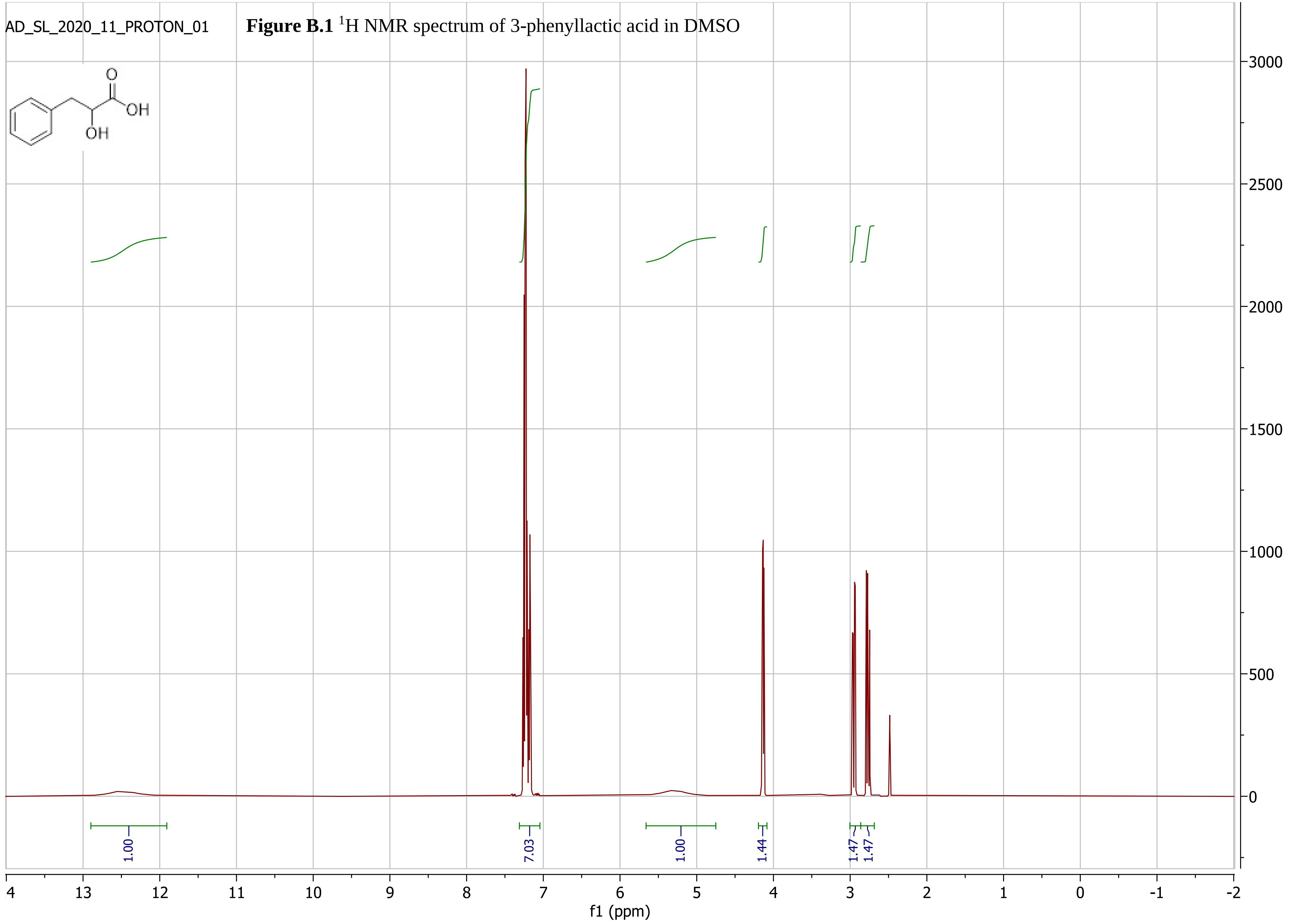

Figure B.1 ^{1}H NMR spectrum of 3-phenyllactic acid in DMSO

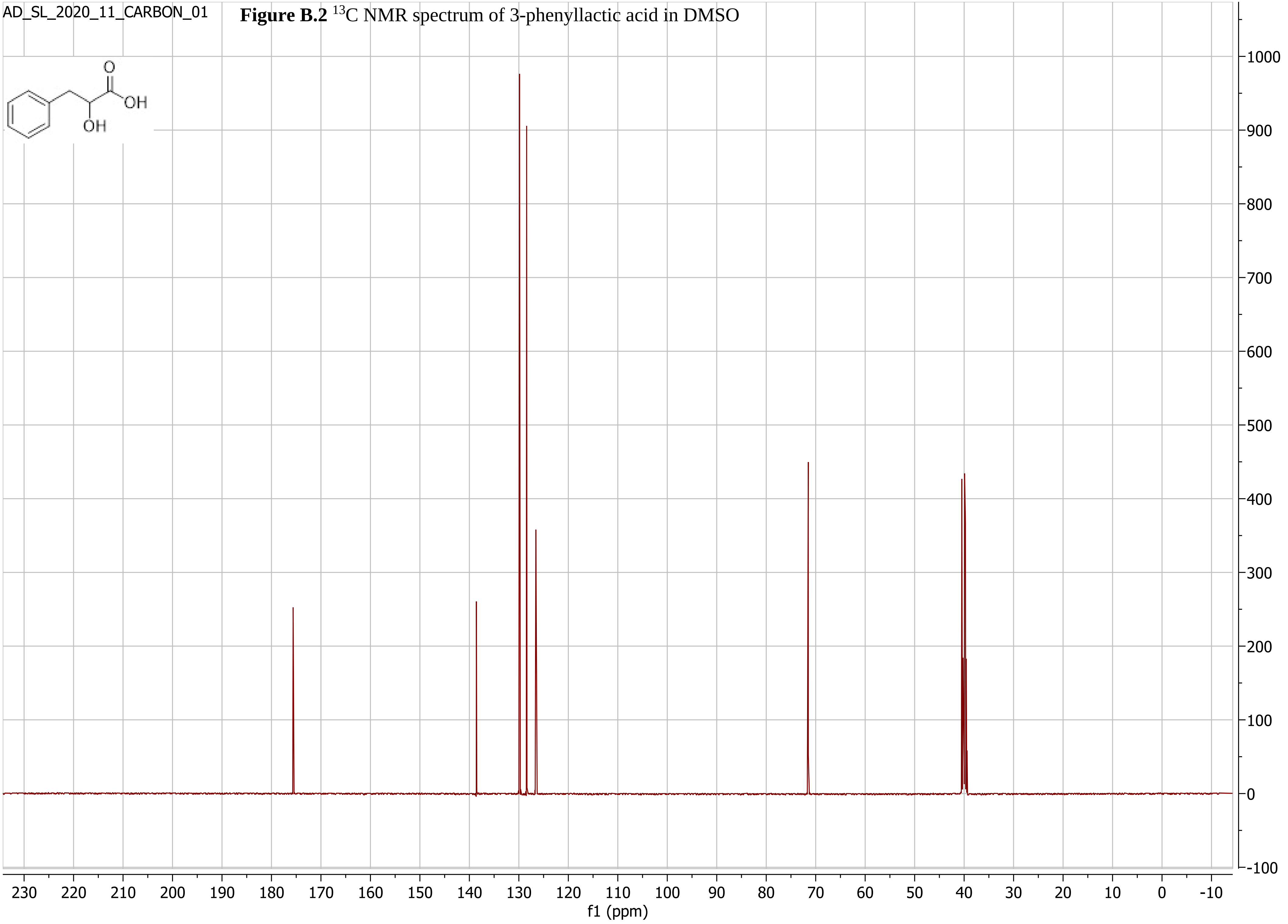

Figure B.2 ^{13}C NMR spectrum of 3-phenyllactic acid in DMSO

Figure B.3 HSQC NMR spectrum of 3-phenyllactic acid in DMSO

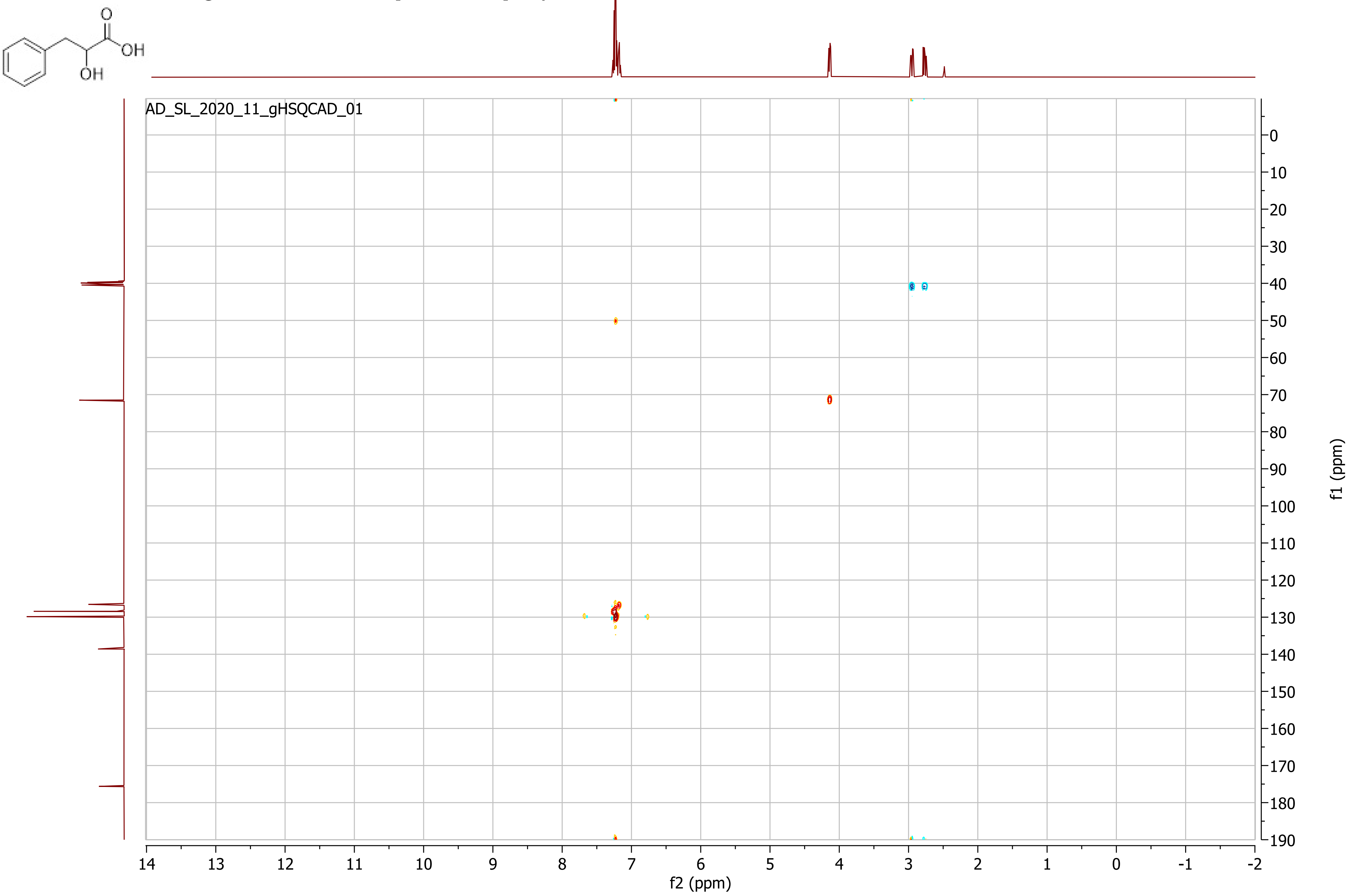

Figure B.4 HMBC NMR spectrum of 3-phenyllactic acid in DMSO

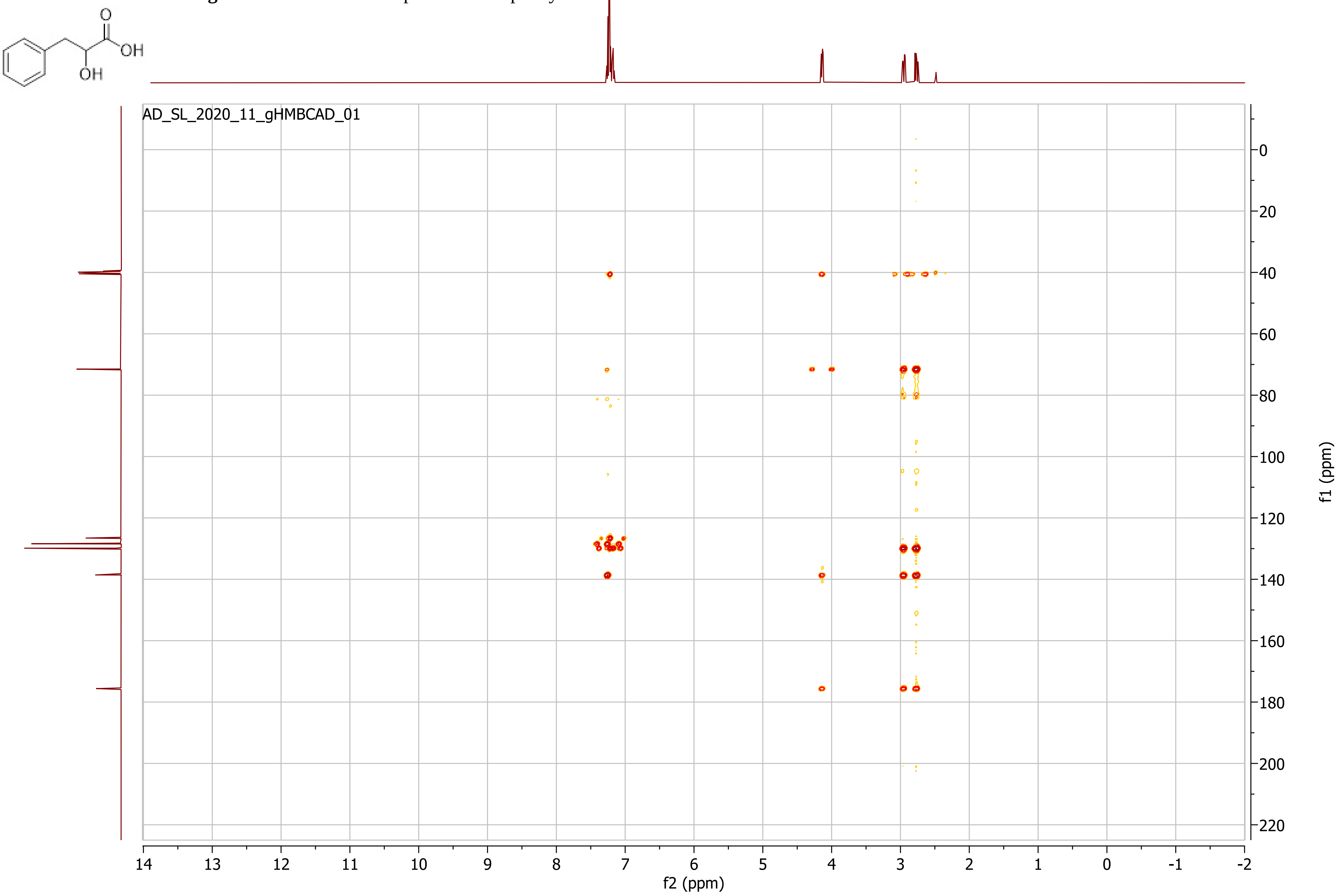

Figure B.5 ^1H-^1H COSY NMR spectrum of 3-phenyllactic acid in DMSO

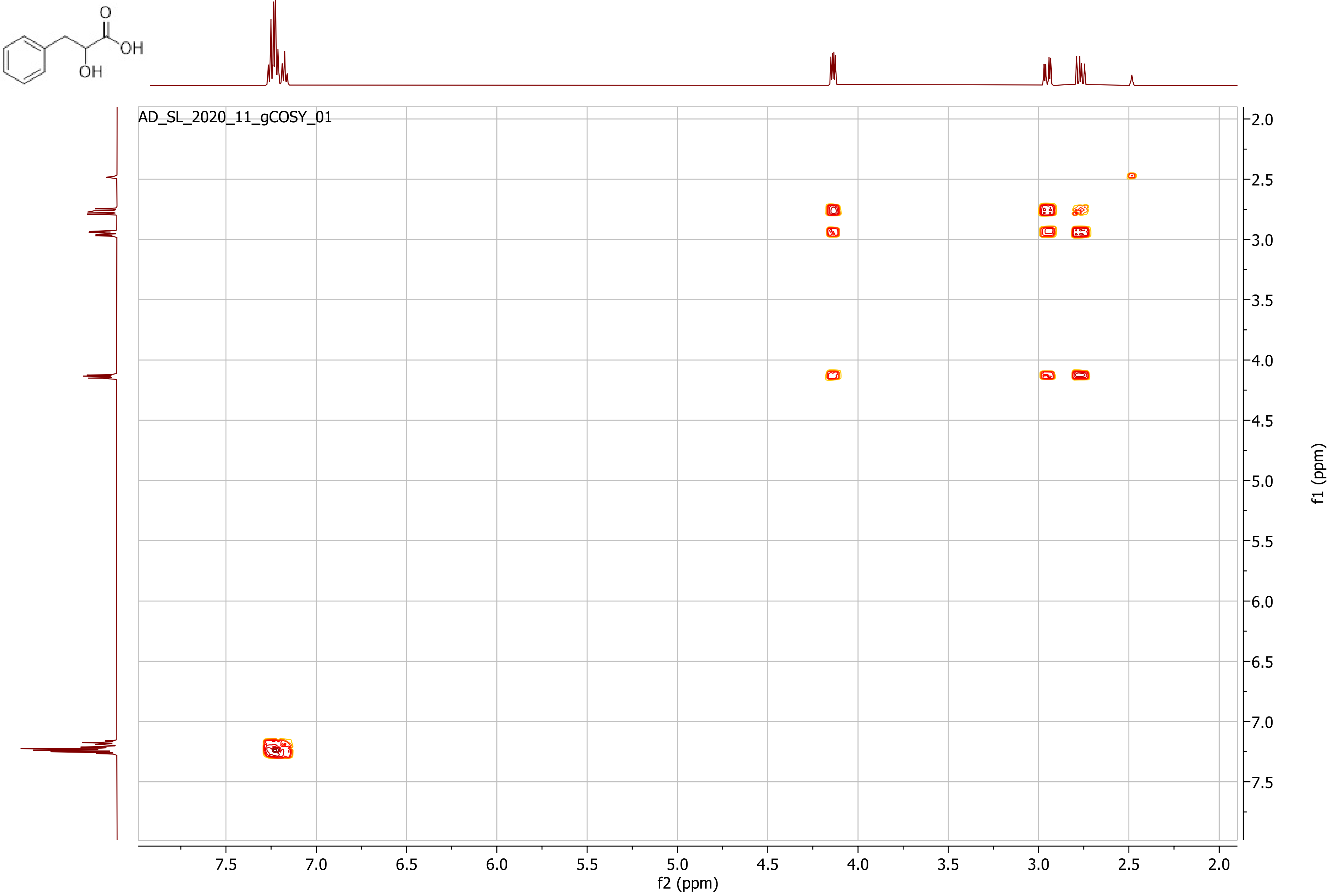

Figure C.1 ^{1}H NMR spectrum of pyruvic acid in D_2O

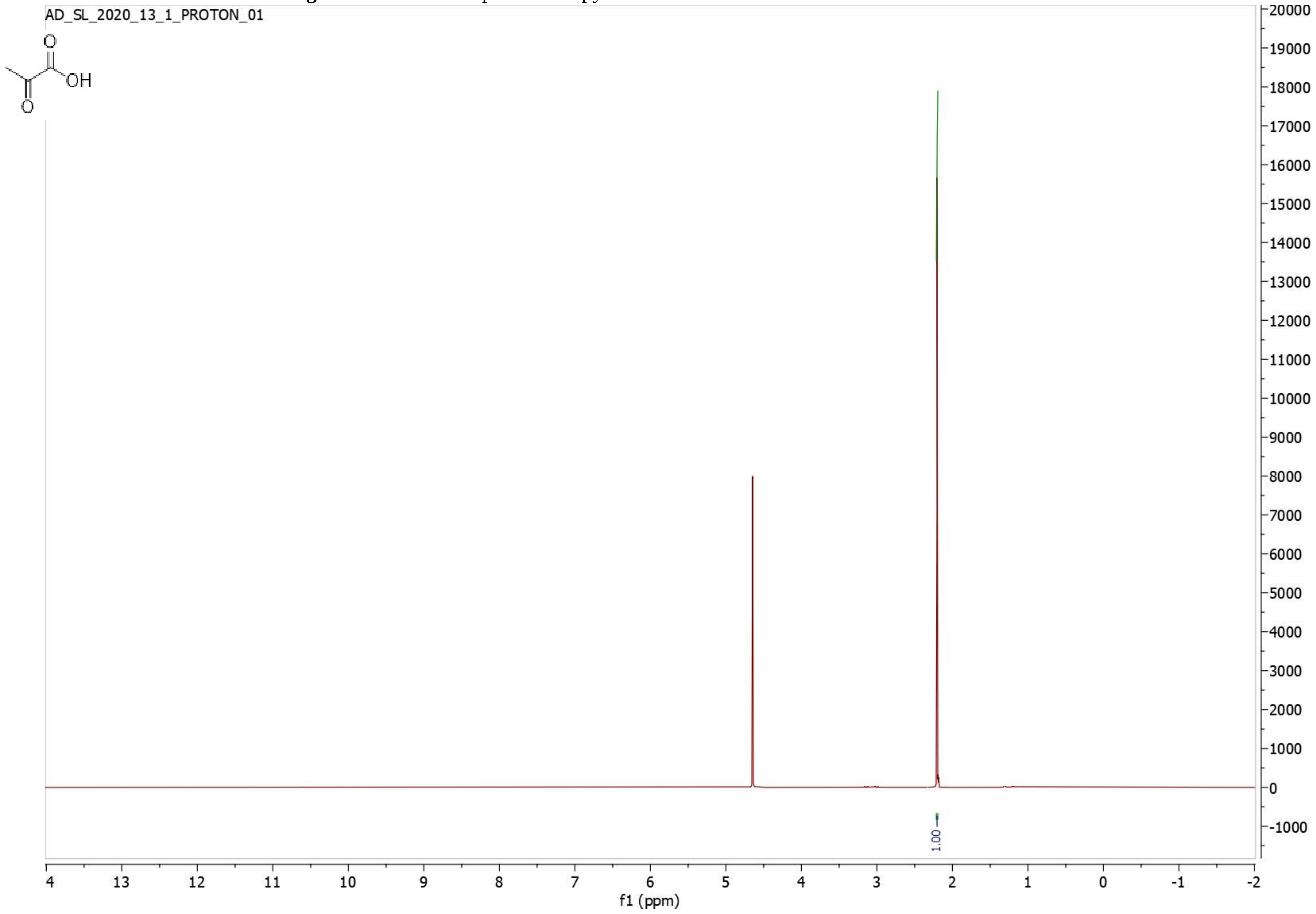

Figure C.2 ^{13}C NMR spectrum of pyruvic acid in D_2O

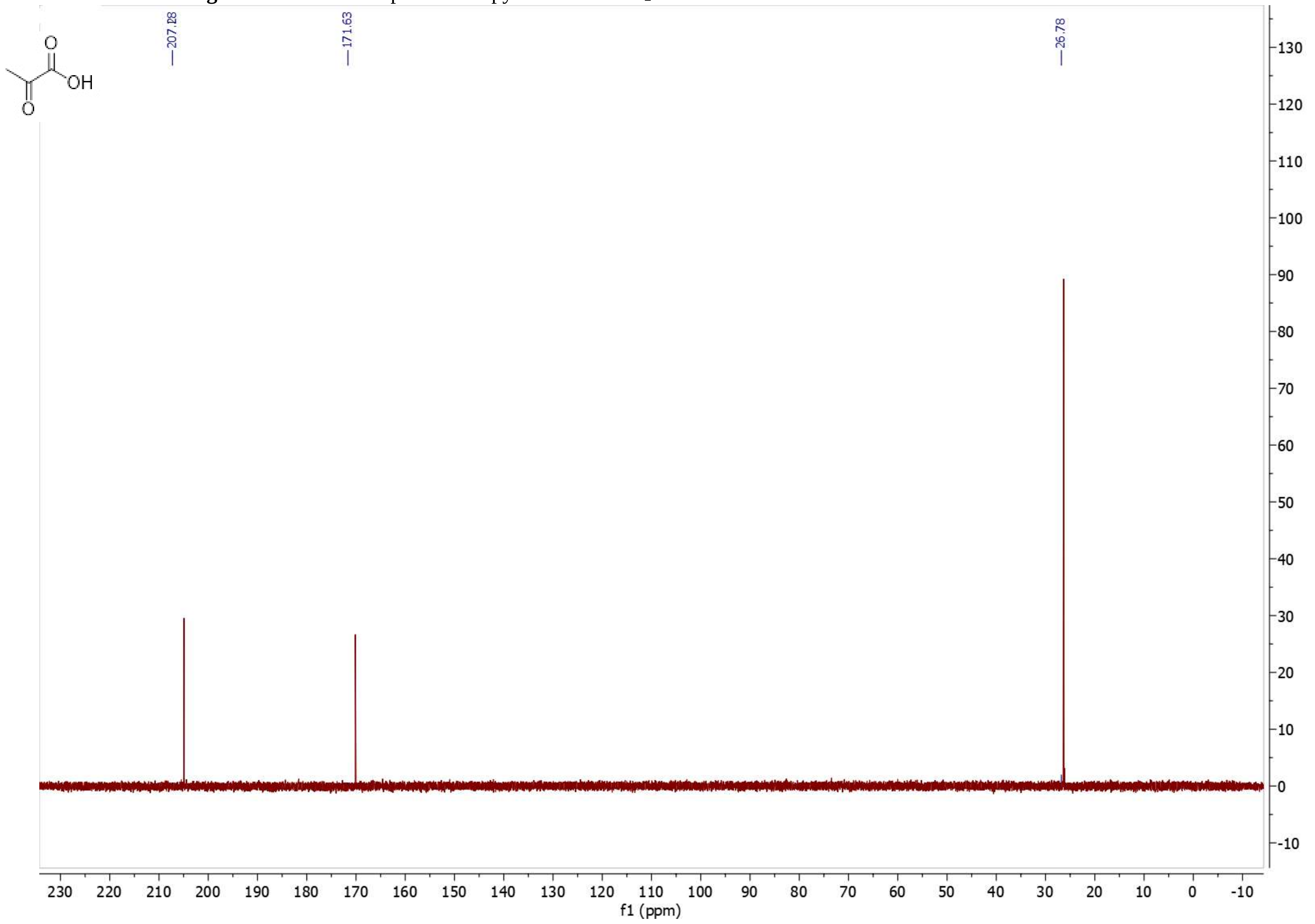

Figure C.3 DEPT NMR spectrum of pyruvic acid in D_2O

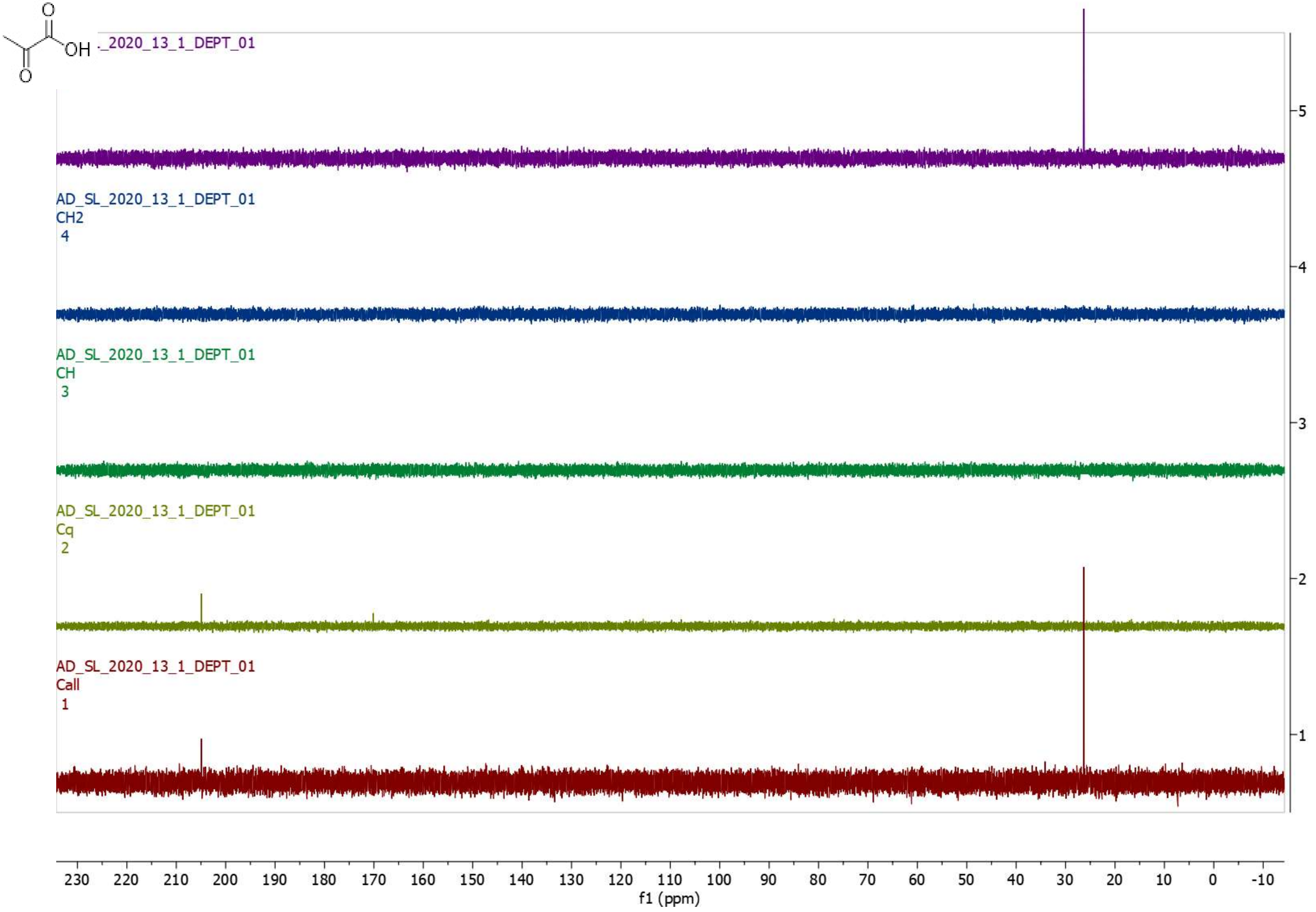

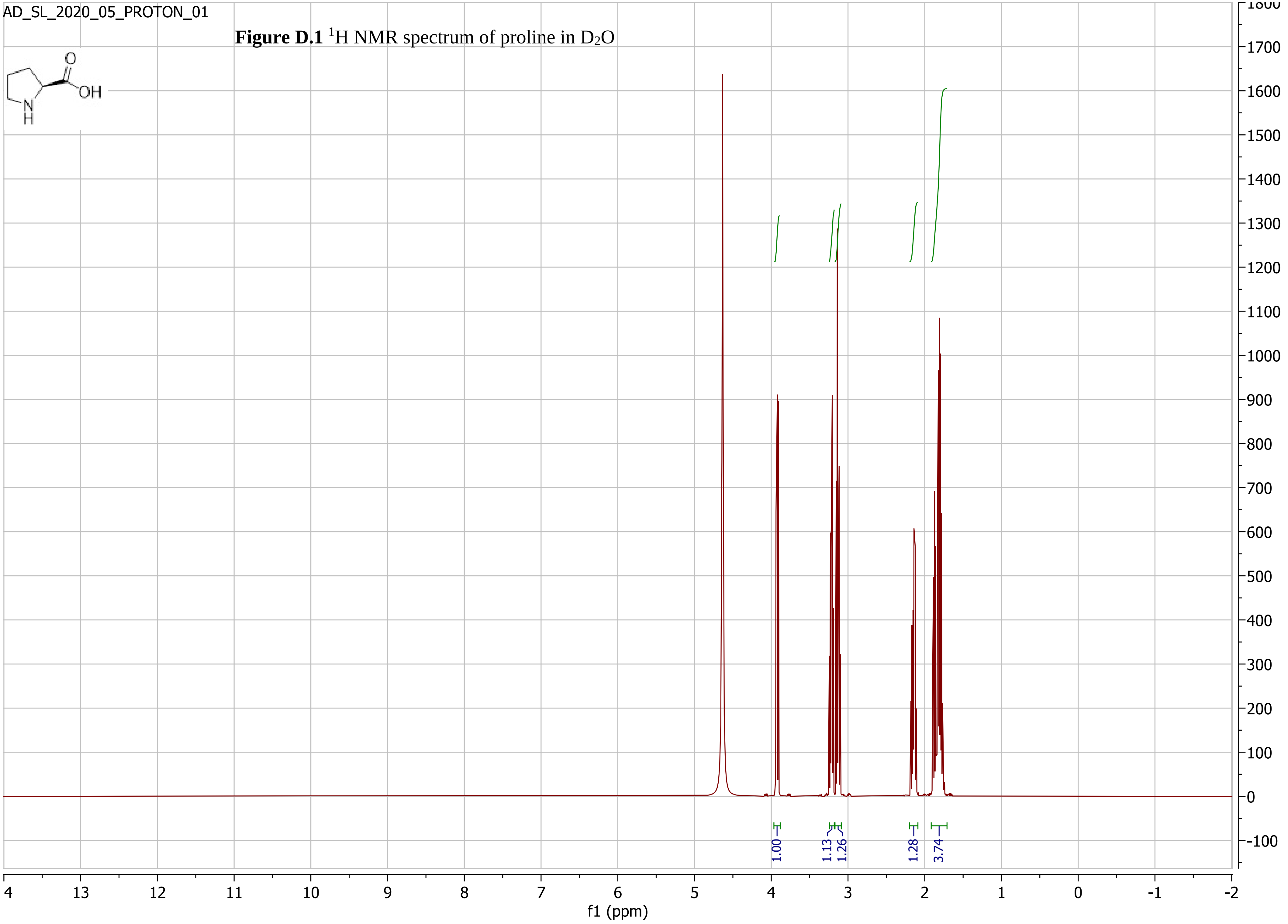

Figure D.1 ^{1}H NMR spectrum of proline in D_2O

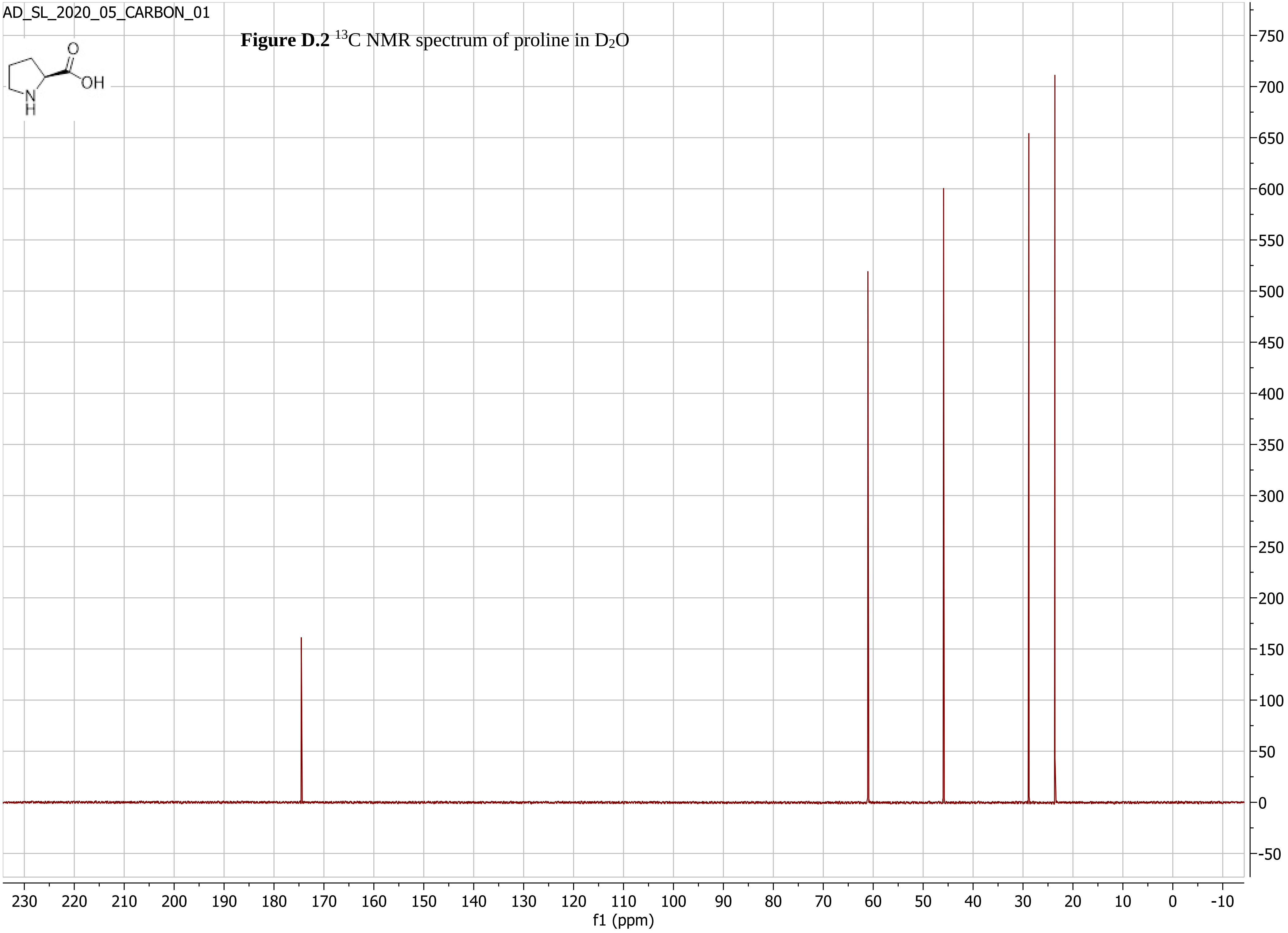

Figure D.2 ^{13}C NMR spectrum of proline in D_2O

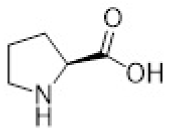

Figure D.3 HSQC NMR spectrum of proline in D_2O

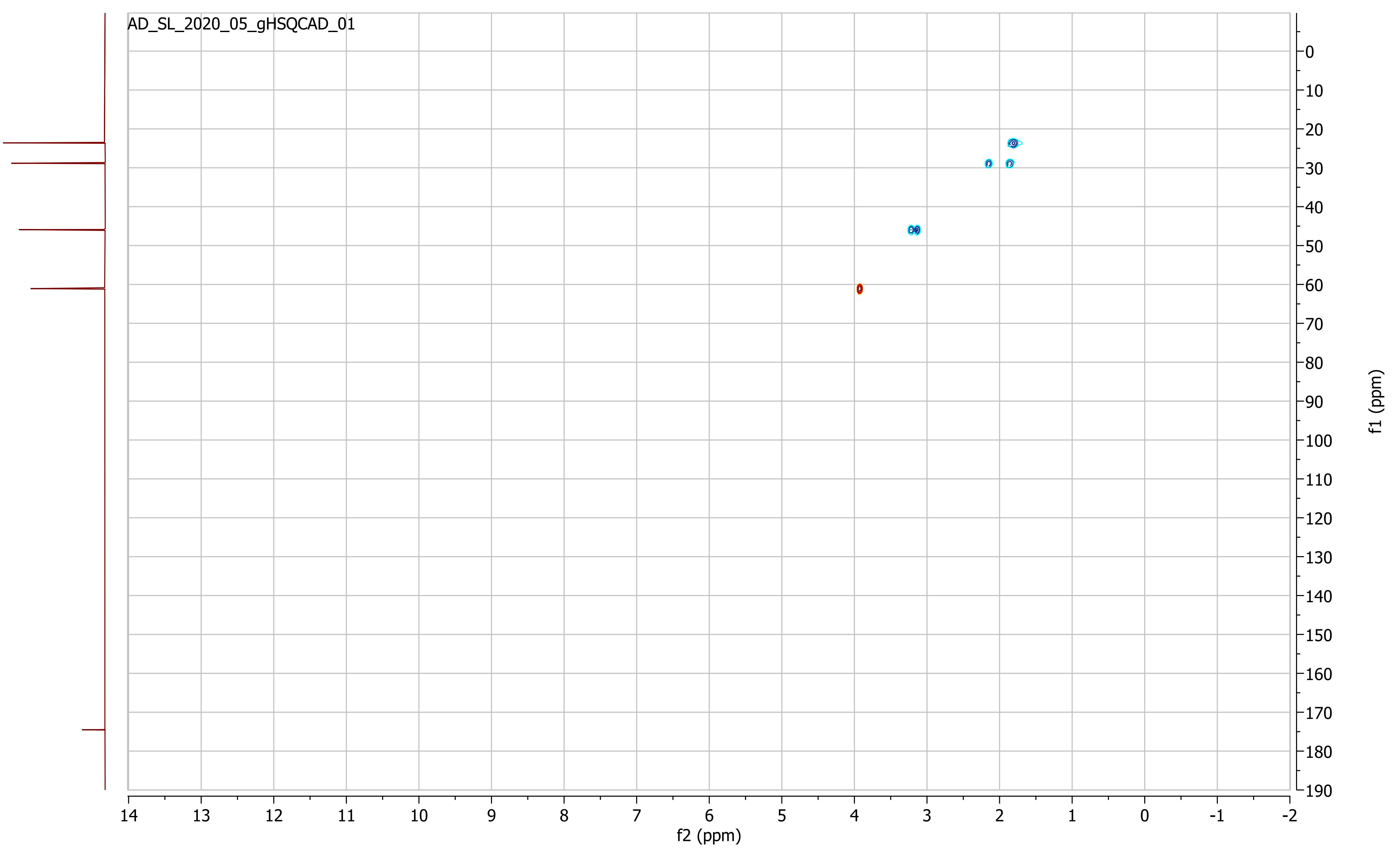

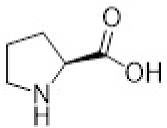

Figure D.4 HMBC NMR spectrum of proline in D_2O

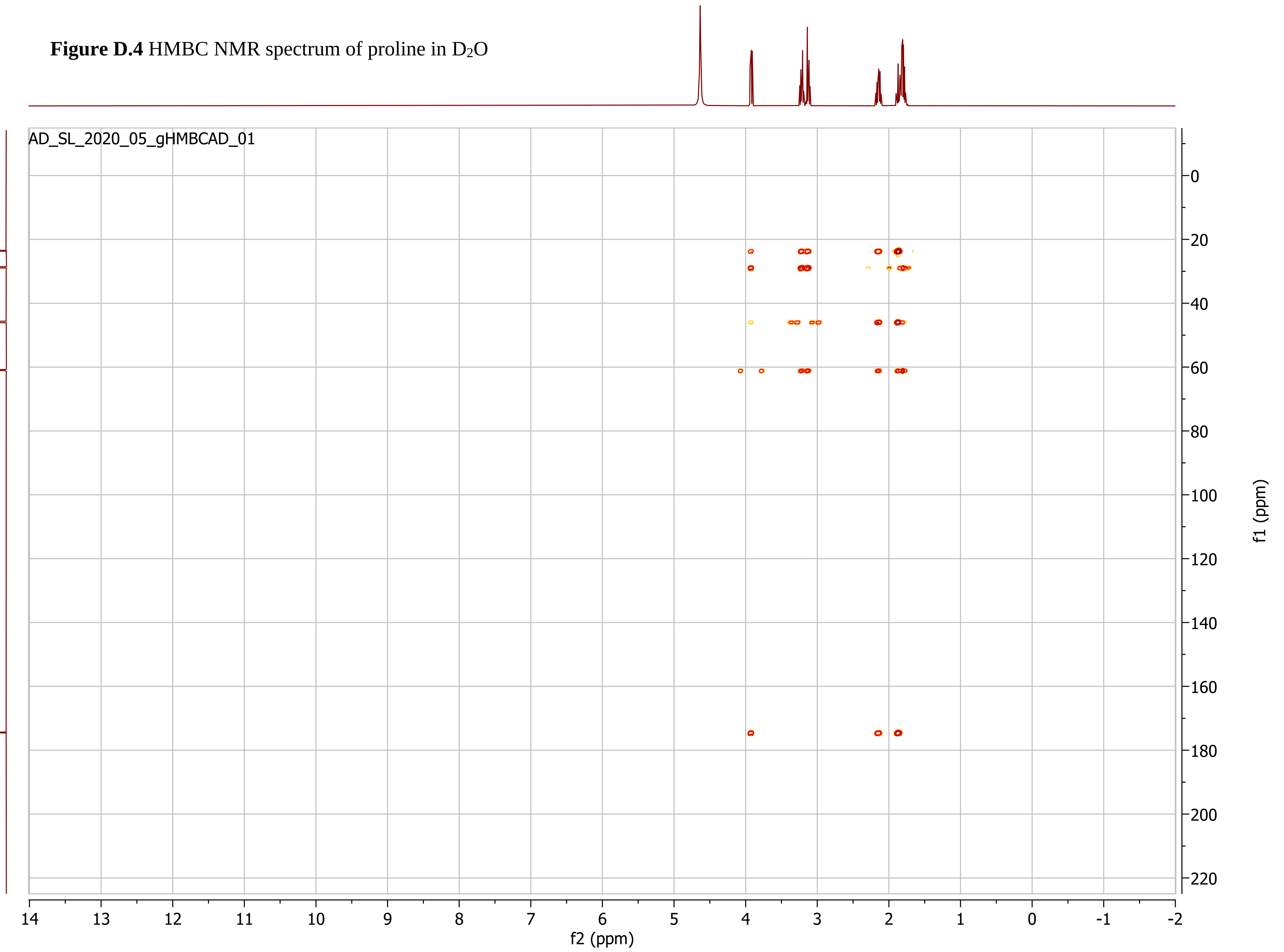

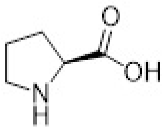

Figure D.5 ^{1}H-^{1}H COSY NMR spectrum of proline in D_2O

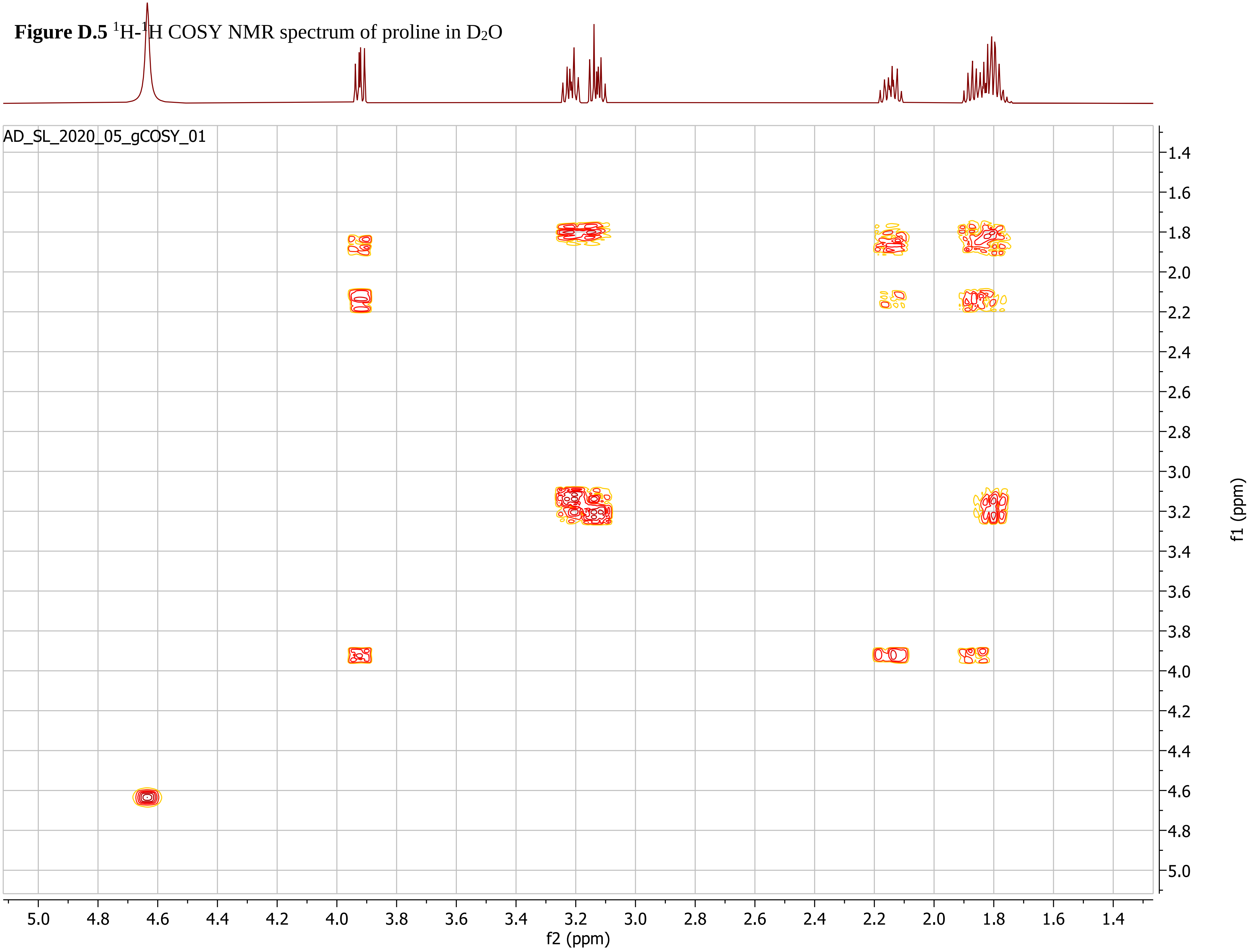

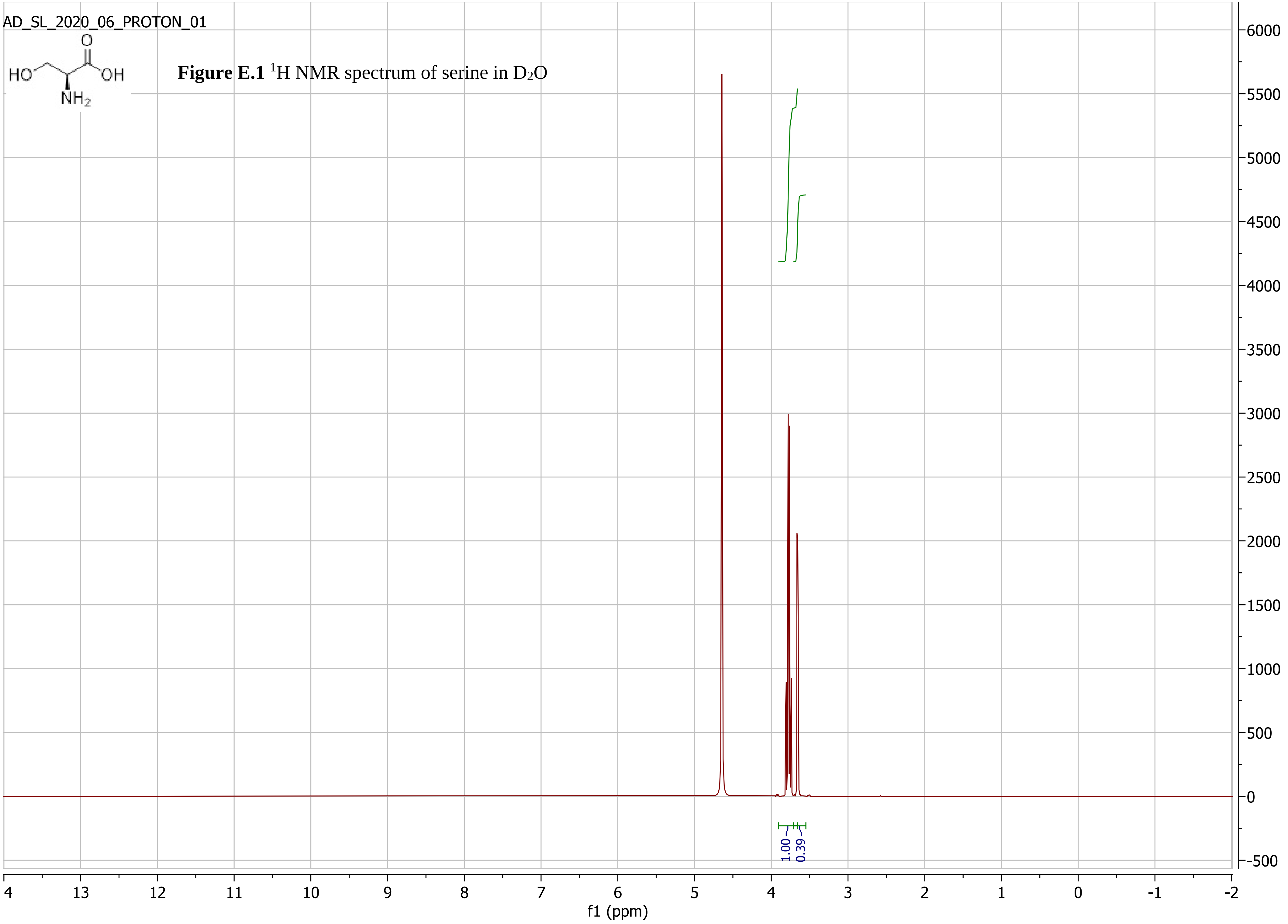

Figure E.1 ^{1}H NMR spectrum of serine in D_2O

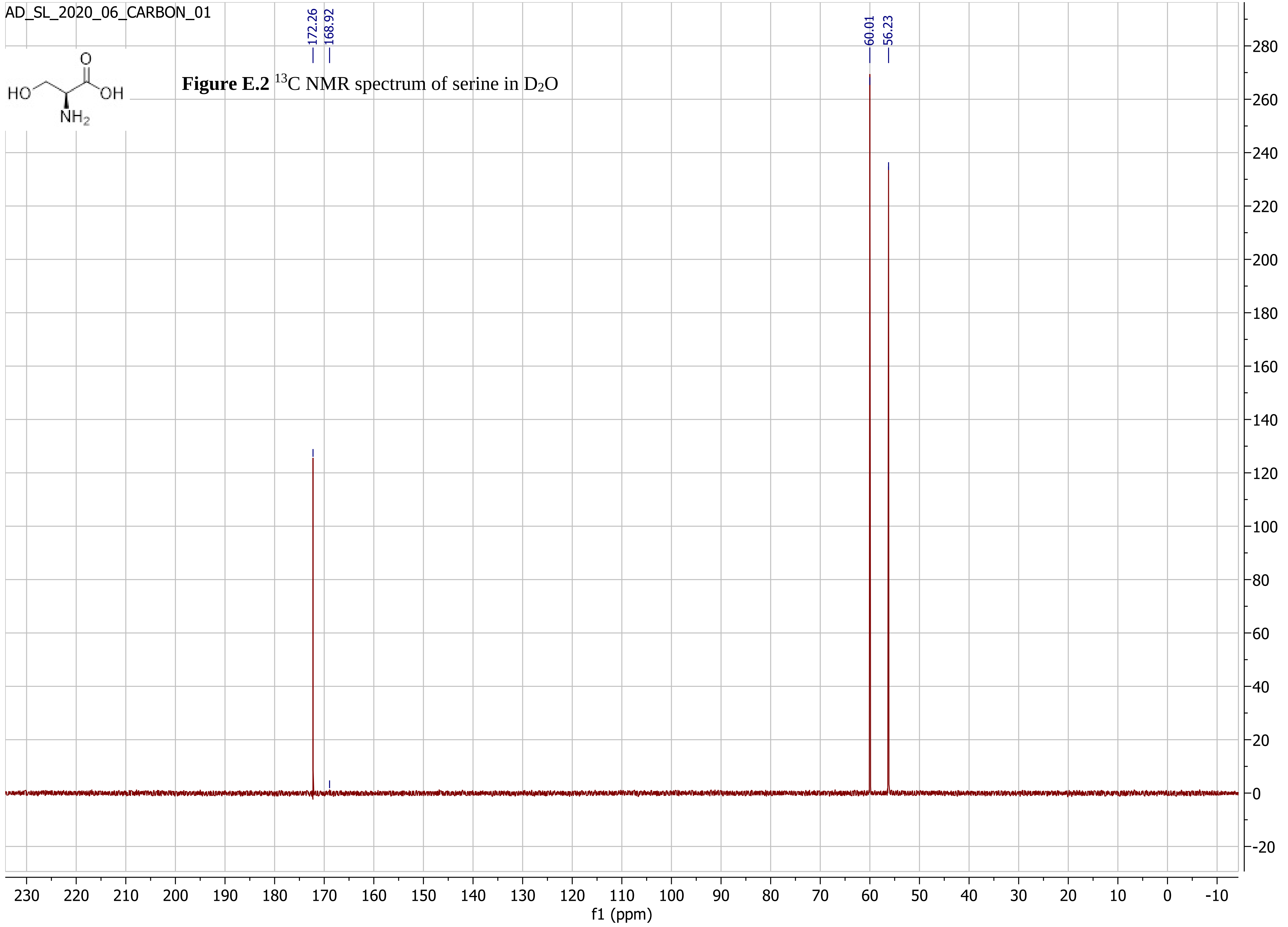

Figure E.2 ^{13}C NMR spectrum of serine in D_2O

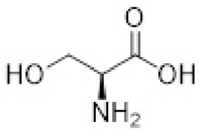

Figure E.3 HSQC NMR spectrum of serine in D_2O

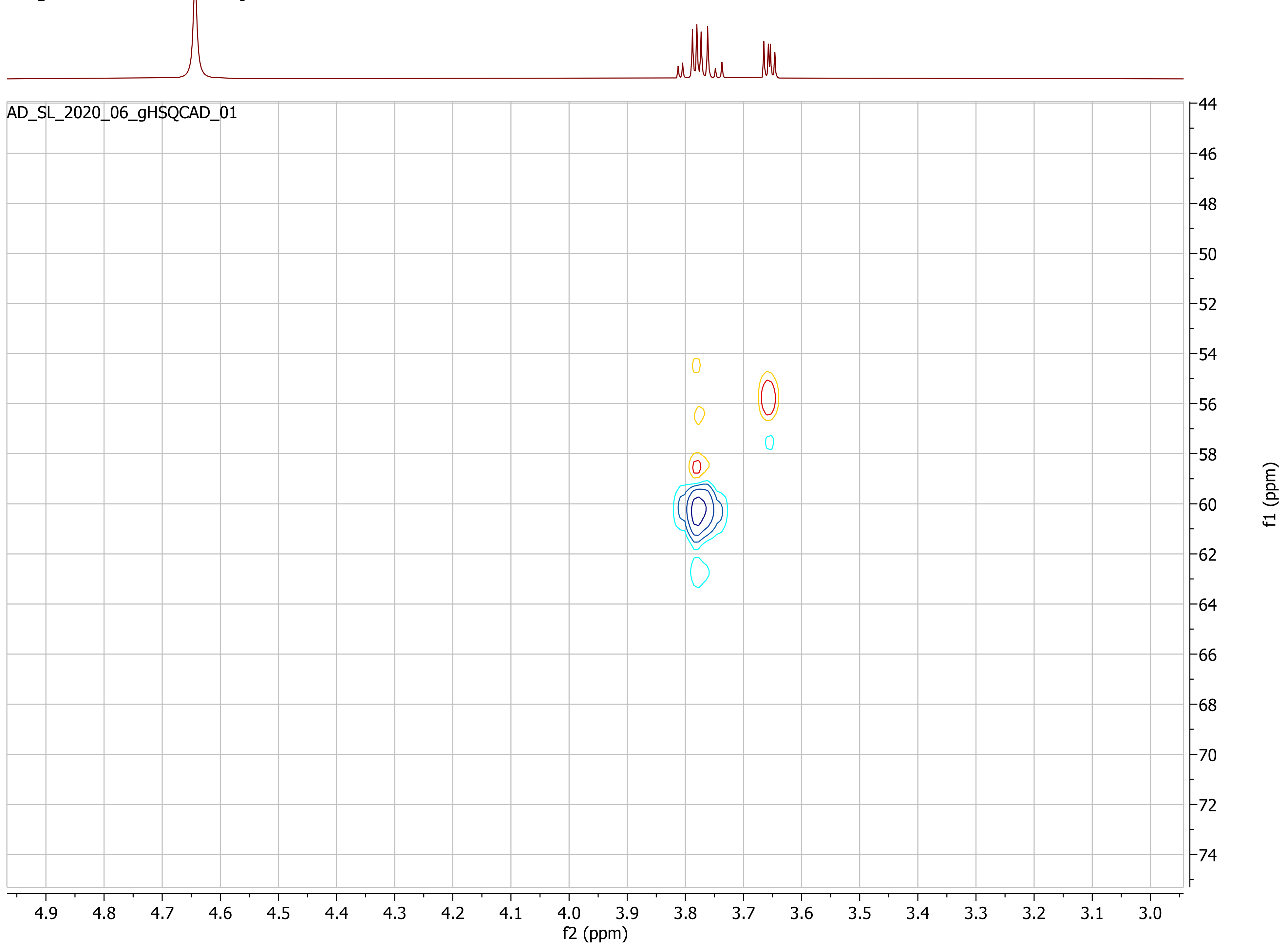

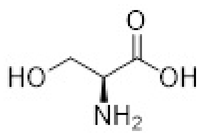

Figure E.4 HMBC NMR spectrum of serine in D_2O

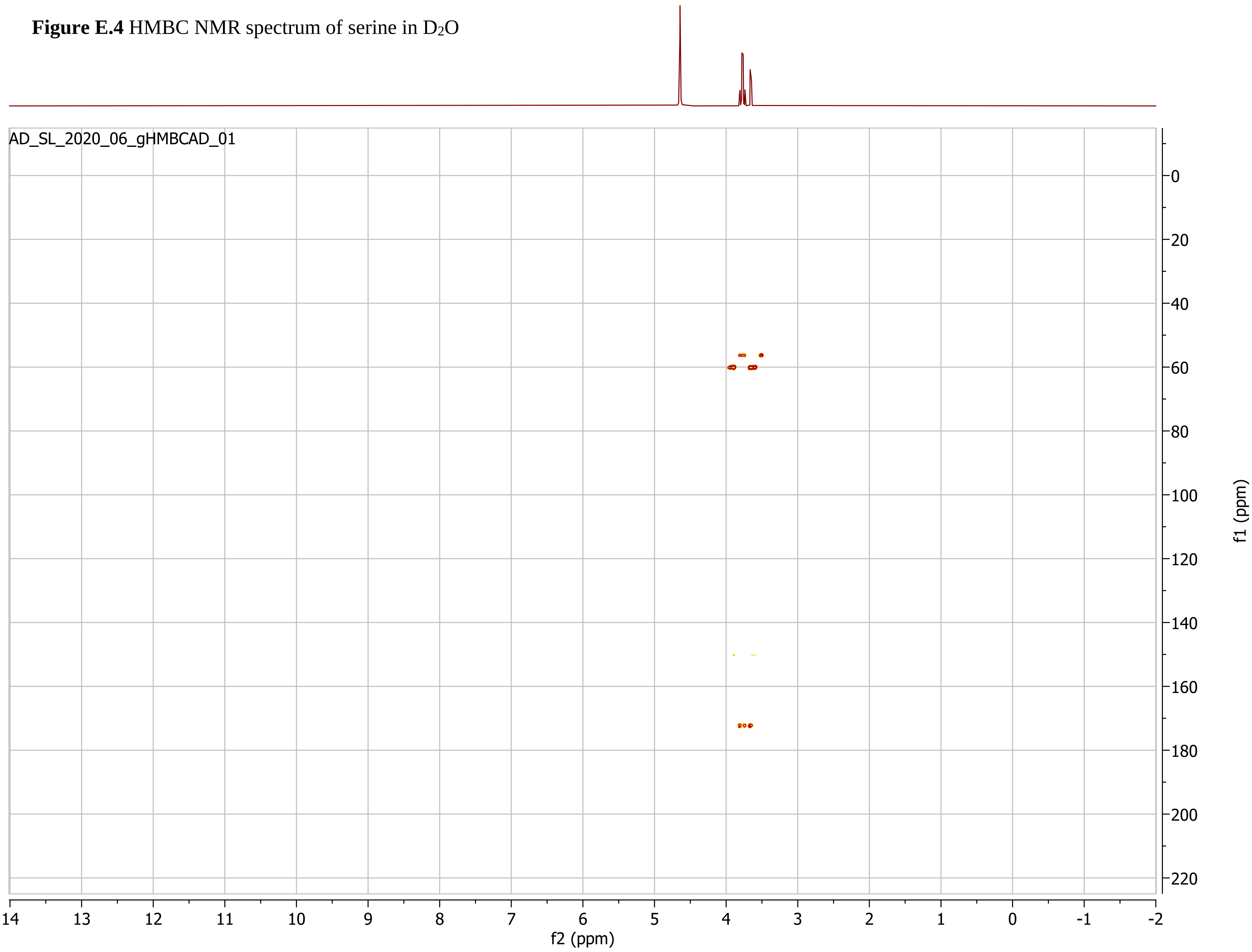

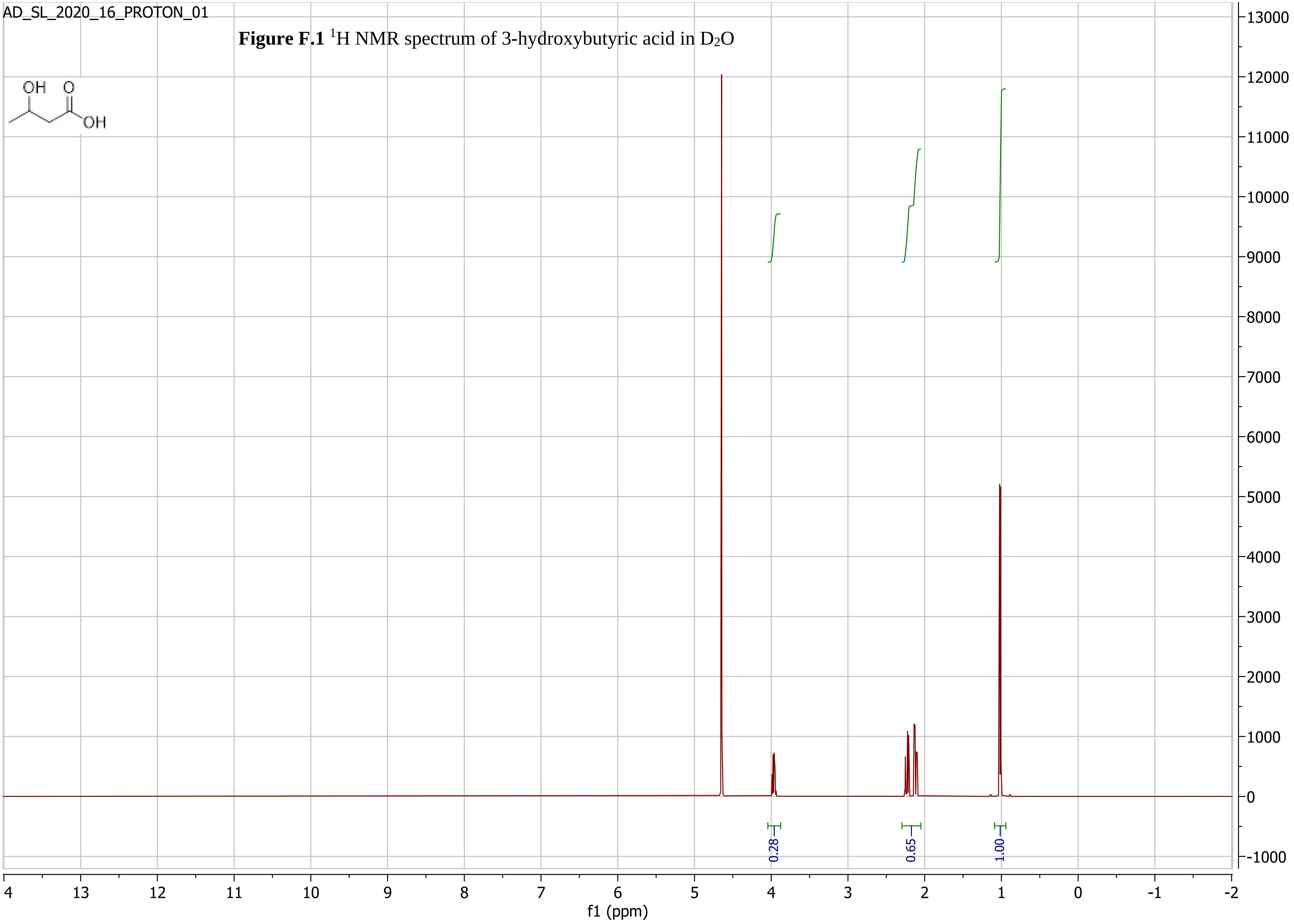

Figure F.1 ^{1}H NMR spectrum of 3-hydroxybutyric acid in D_2O

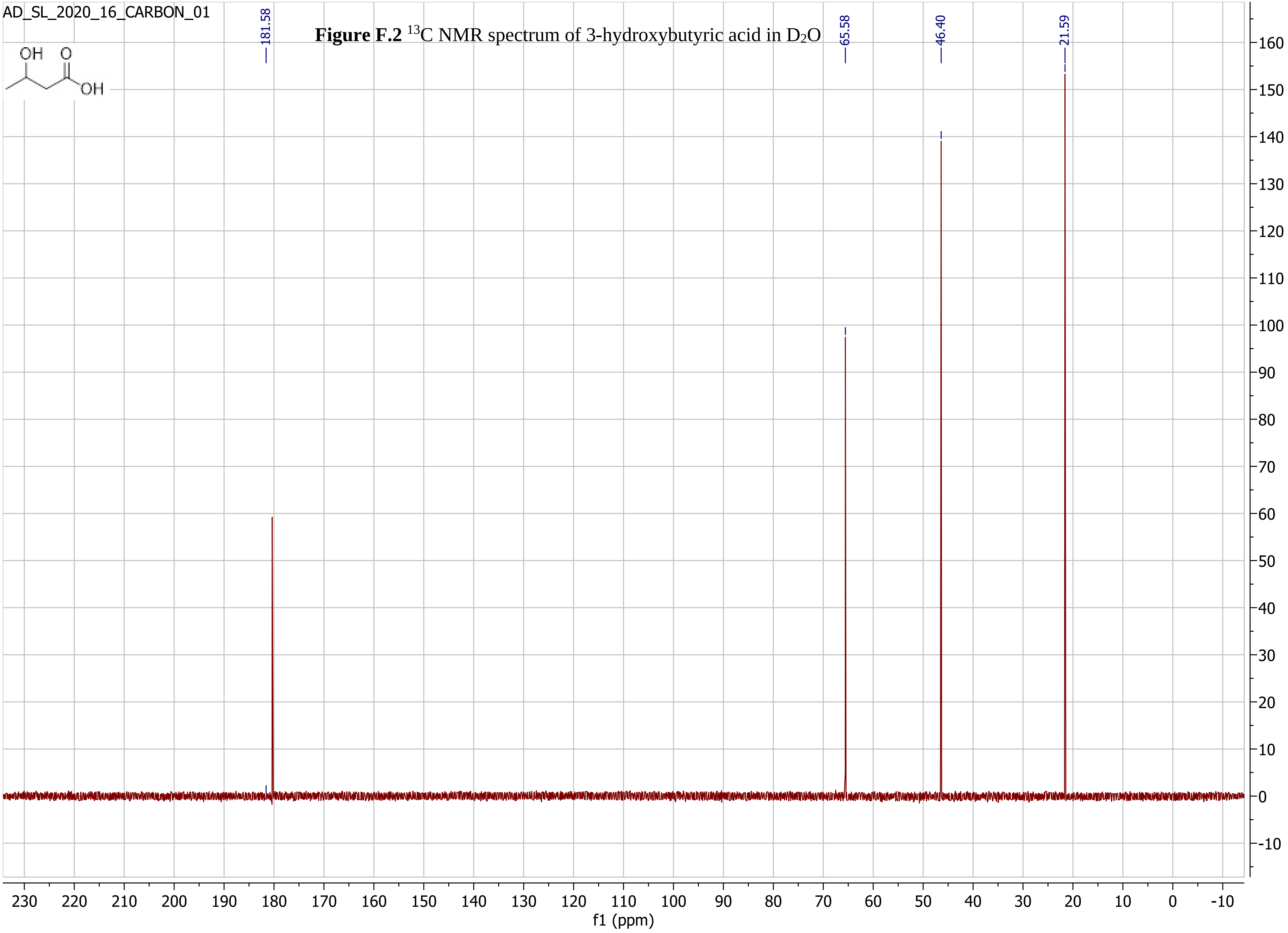

Figure F.2 ^{13}C NMR spectrum of 3-hydroxybutyric acid in D_2O

Figure F.3 DEPT NMR spectrum of 3-hydroxybutyric acid in D_2O

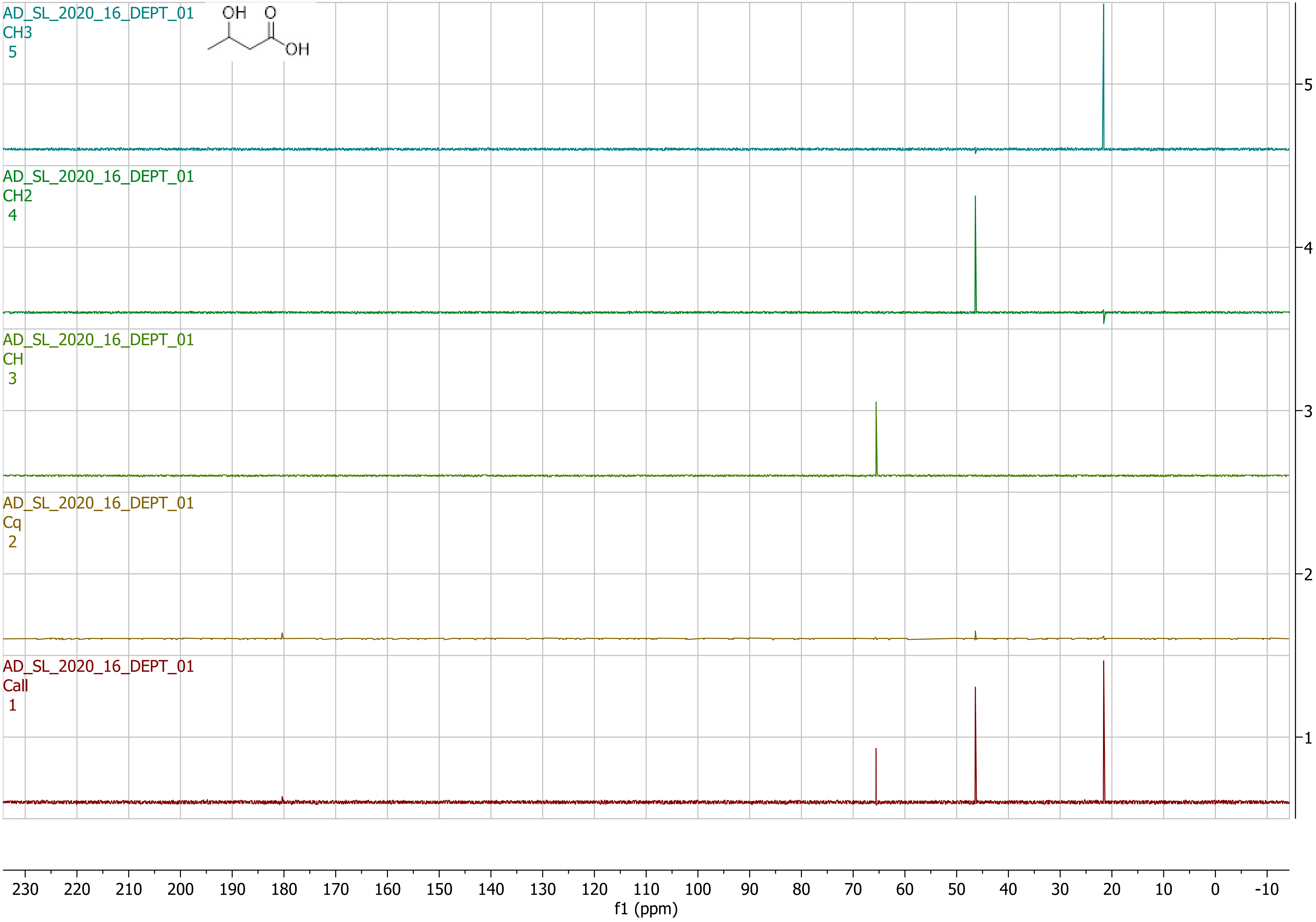

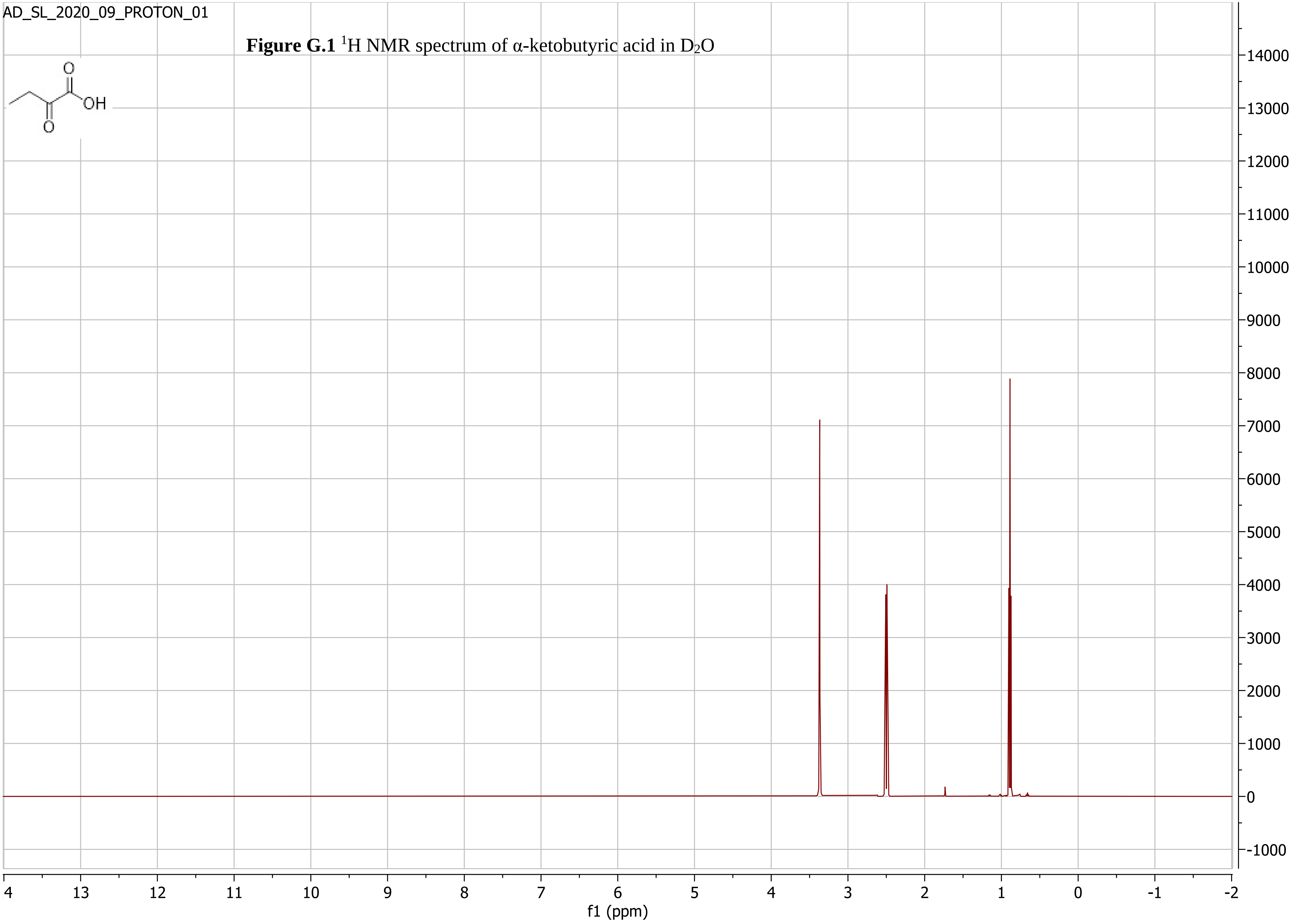

Figure G.1 ^{1}H NMR spectrum of α-ketobutyric acid in D_2O

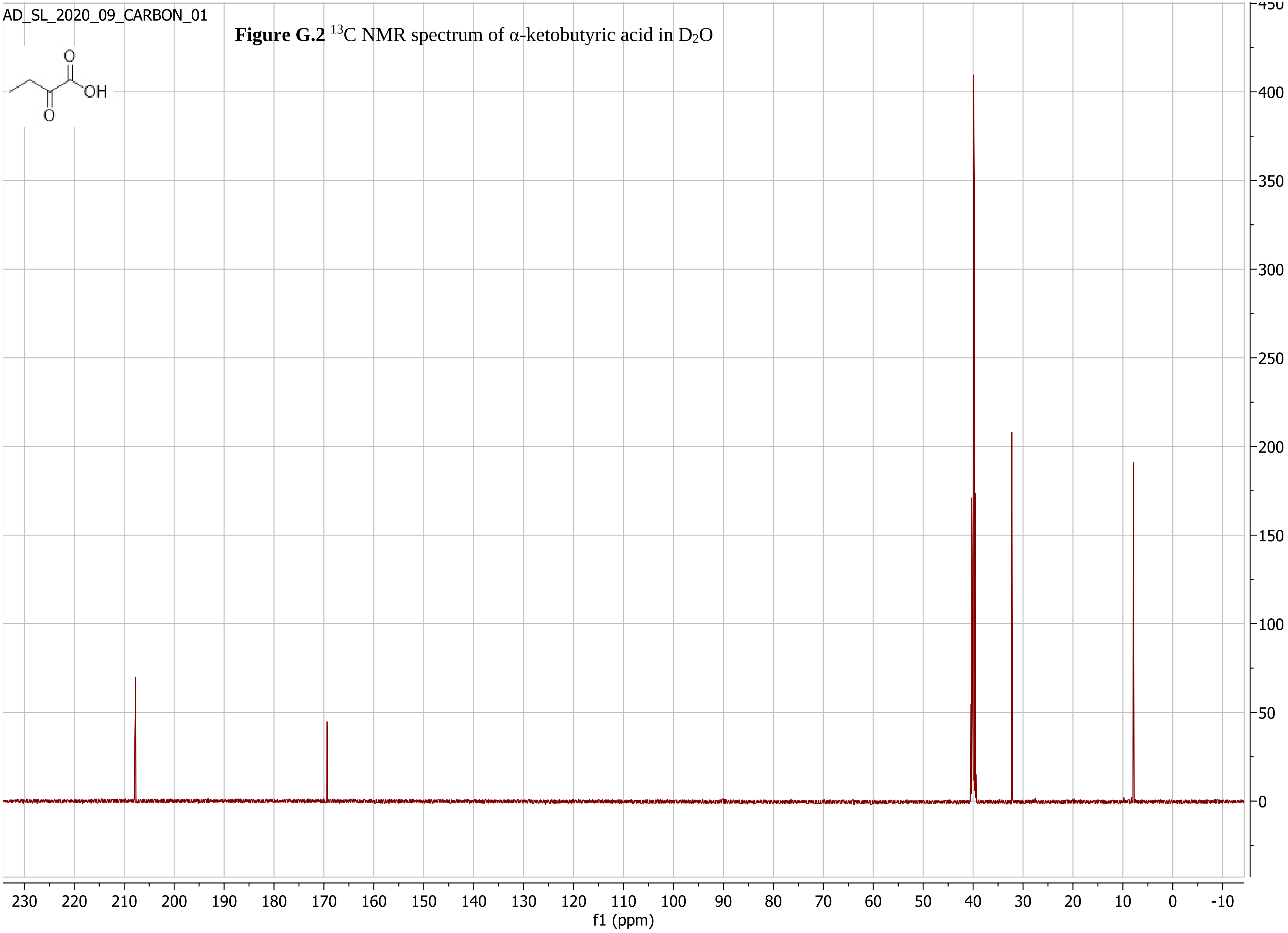

Figure G.2 ^{13}C NMR spectrum of α-ketobutyric acid in D_2O

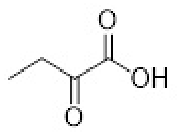

Figure G.3 HSQC NMR spectrum of α-ketobutyric acid in D_2O

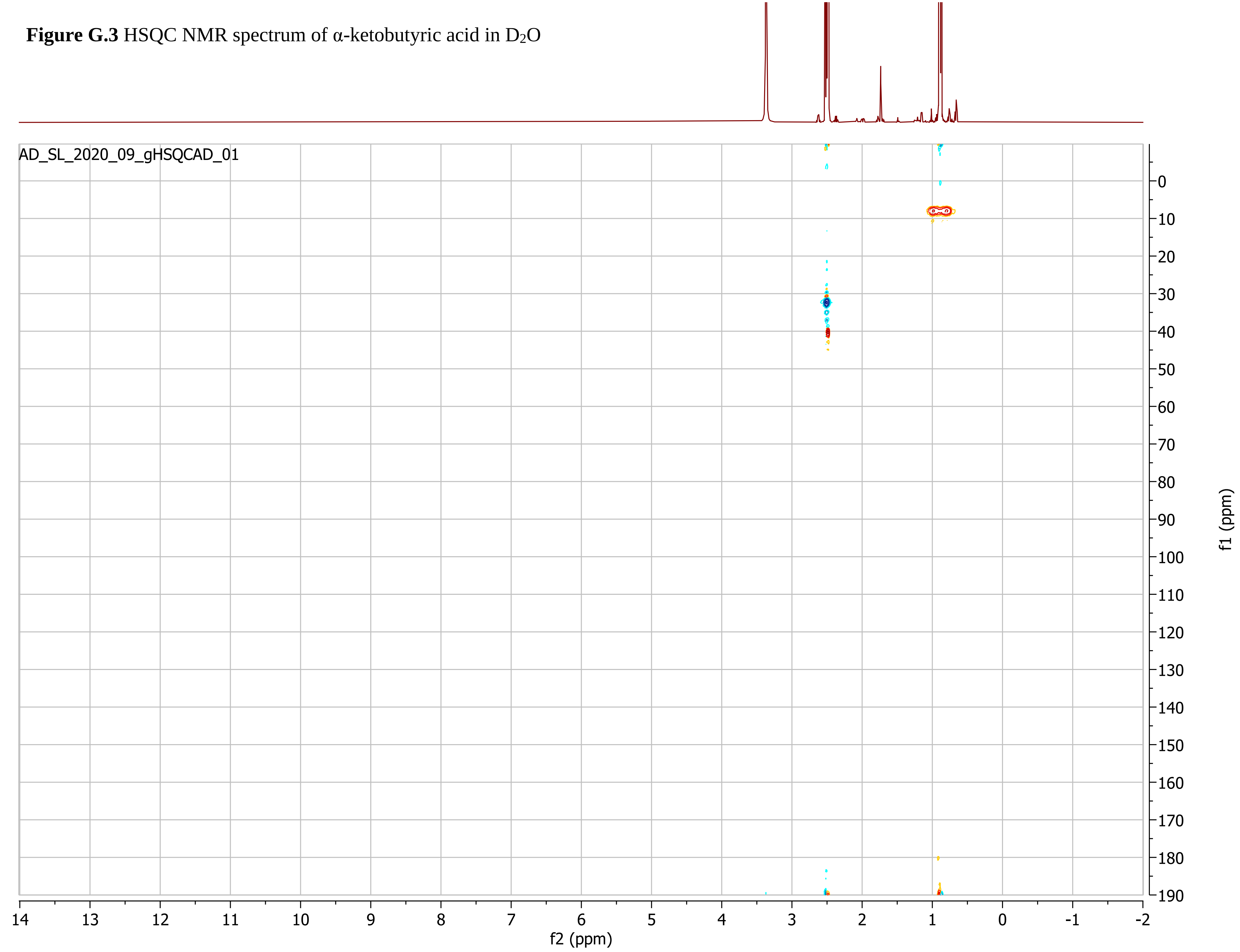

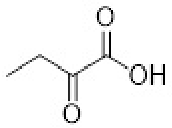

Figure G.4 HMBC NMR spectrum of α-ketobutyric acid in D_2O

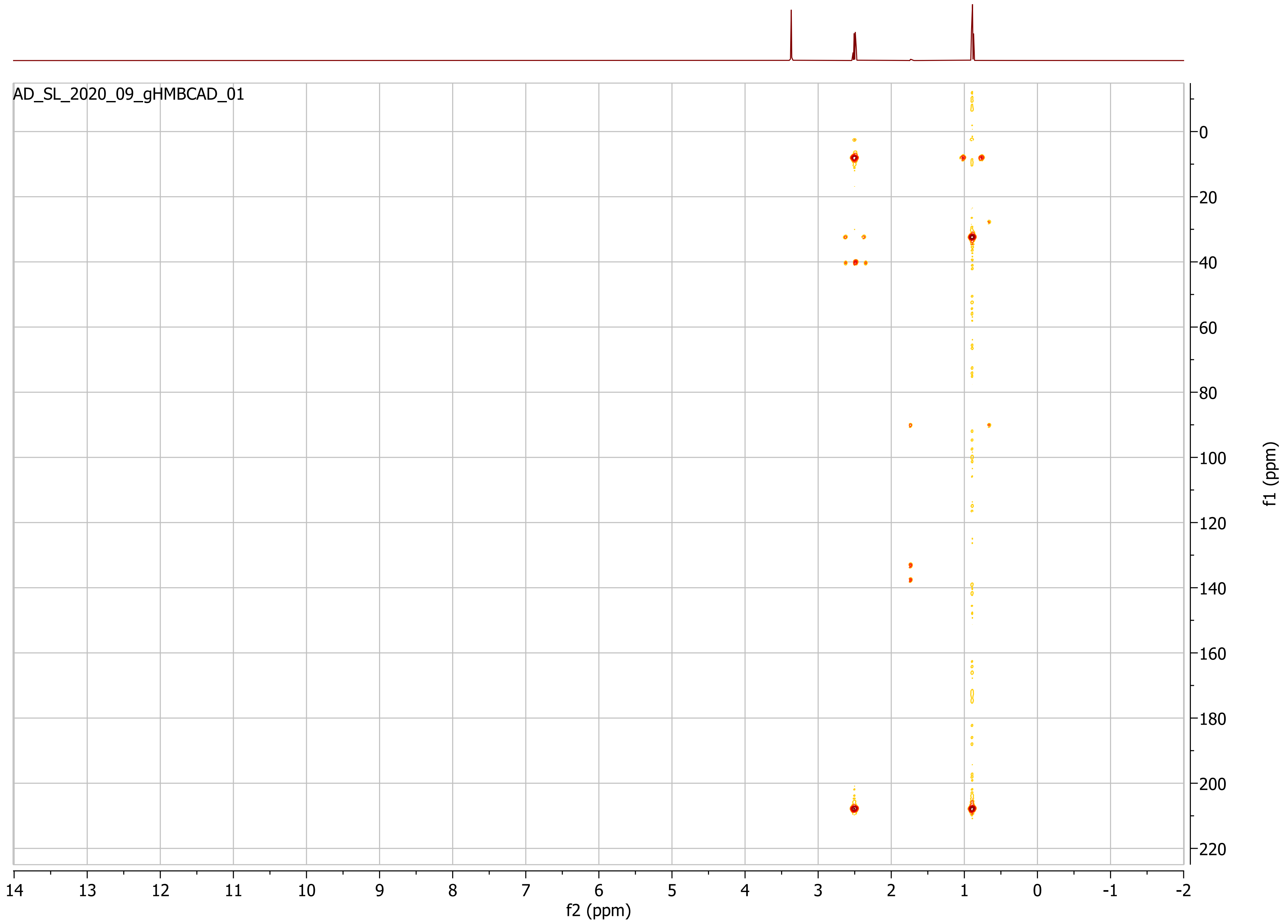

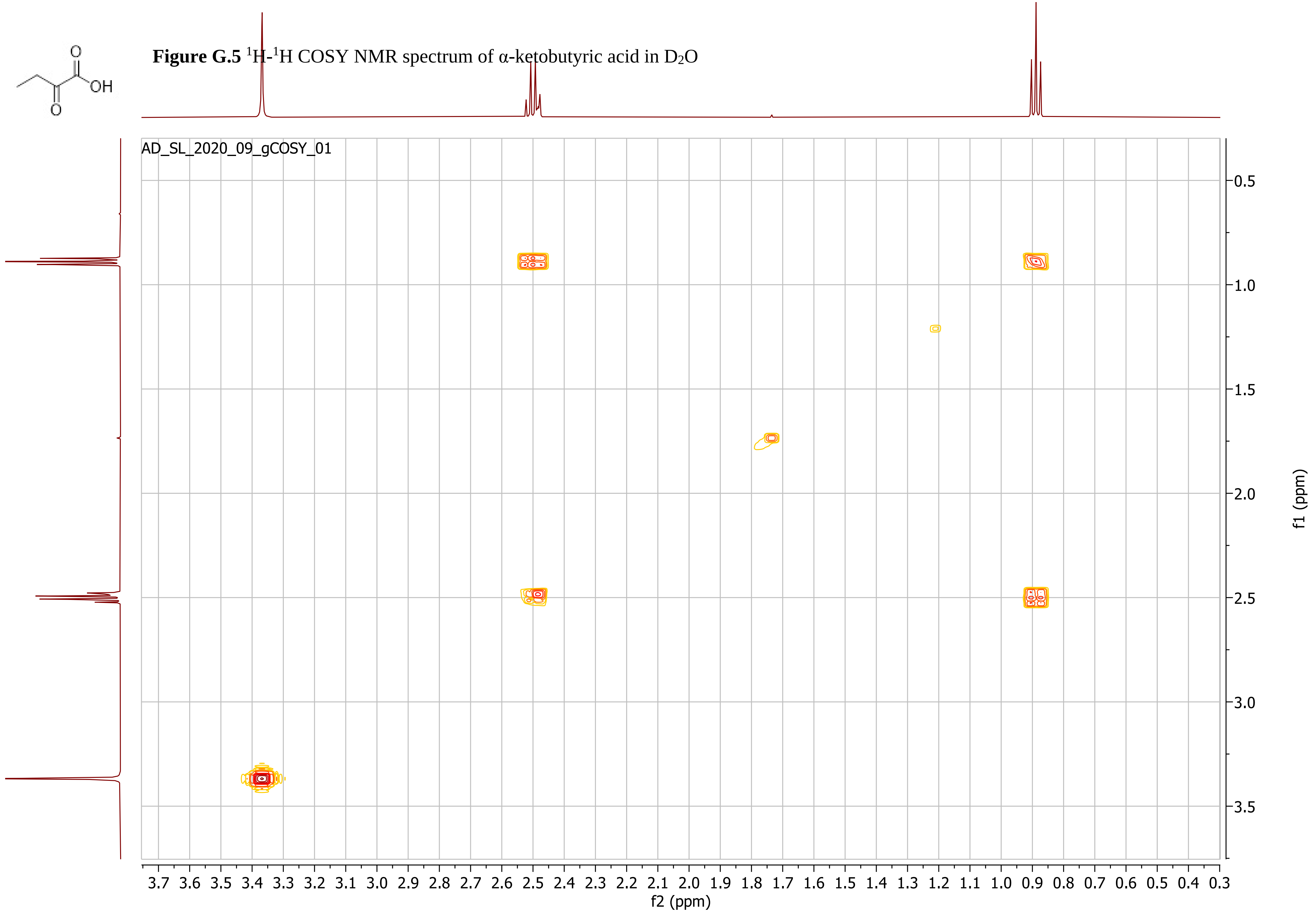

Figure G.5 ^{1}H-^{1}H COSY NMR spectrum of α-ketobutyric acid in D_2O

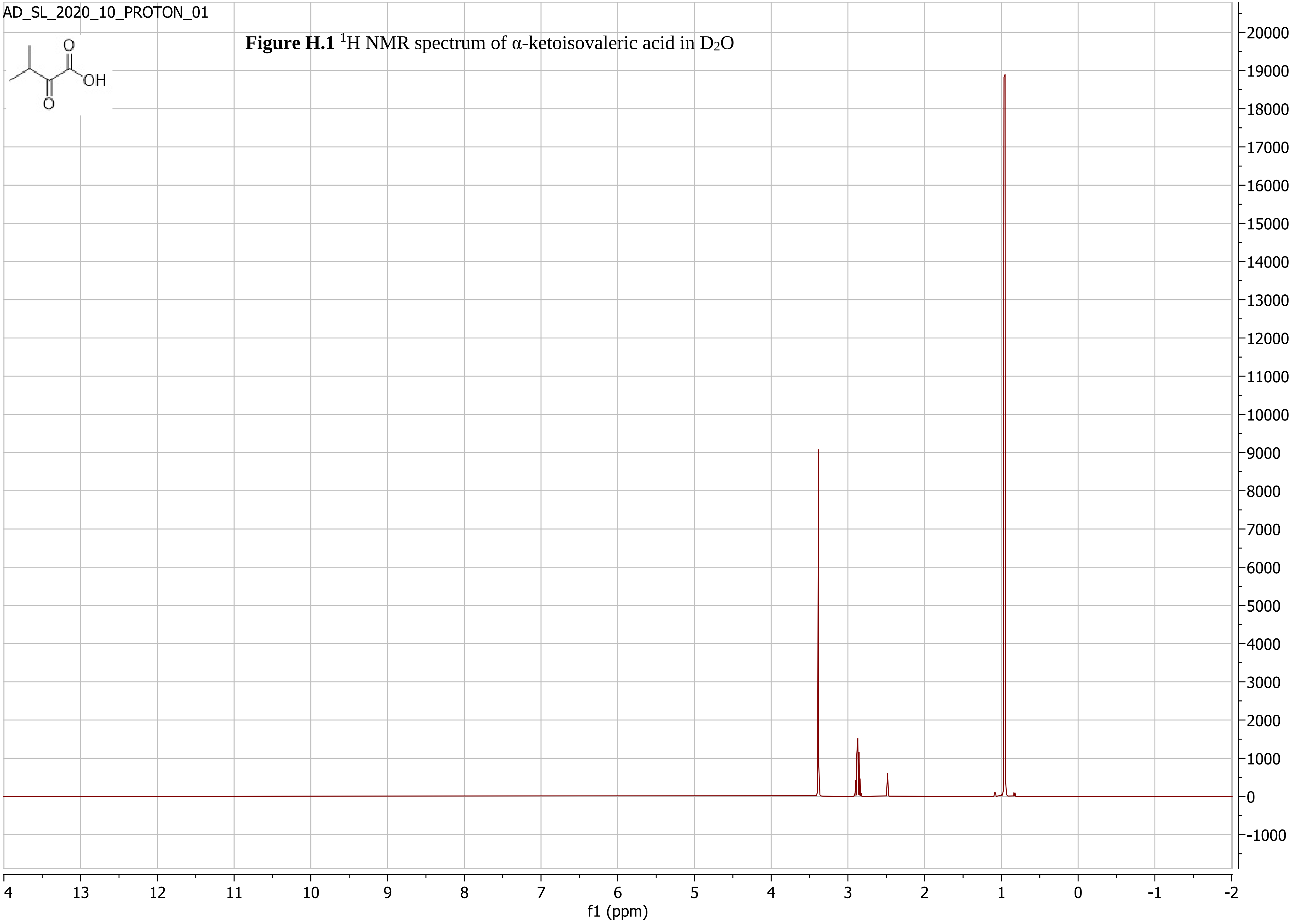

Figure H.1 ^{1}H NMR spectrum of α-ketoisovaleric acid in D_2O

Figure H.2 ^{13}C NMR spectrum of α-ketoisovaleric acid in D_2O

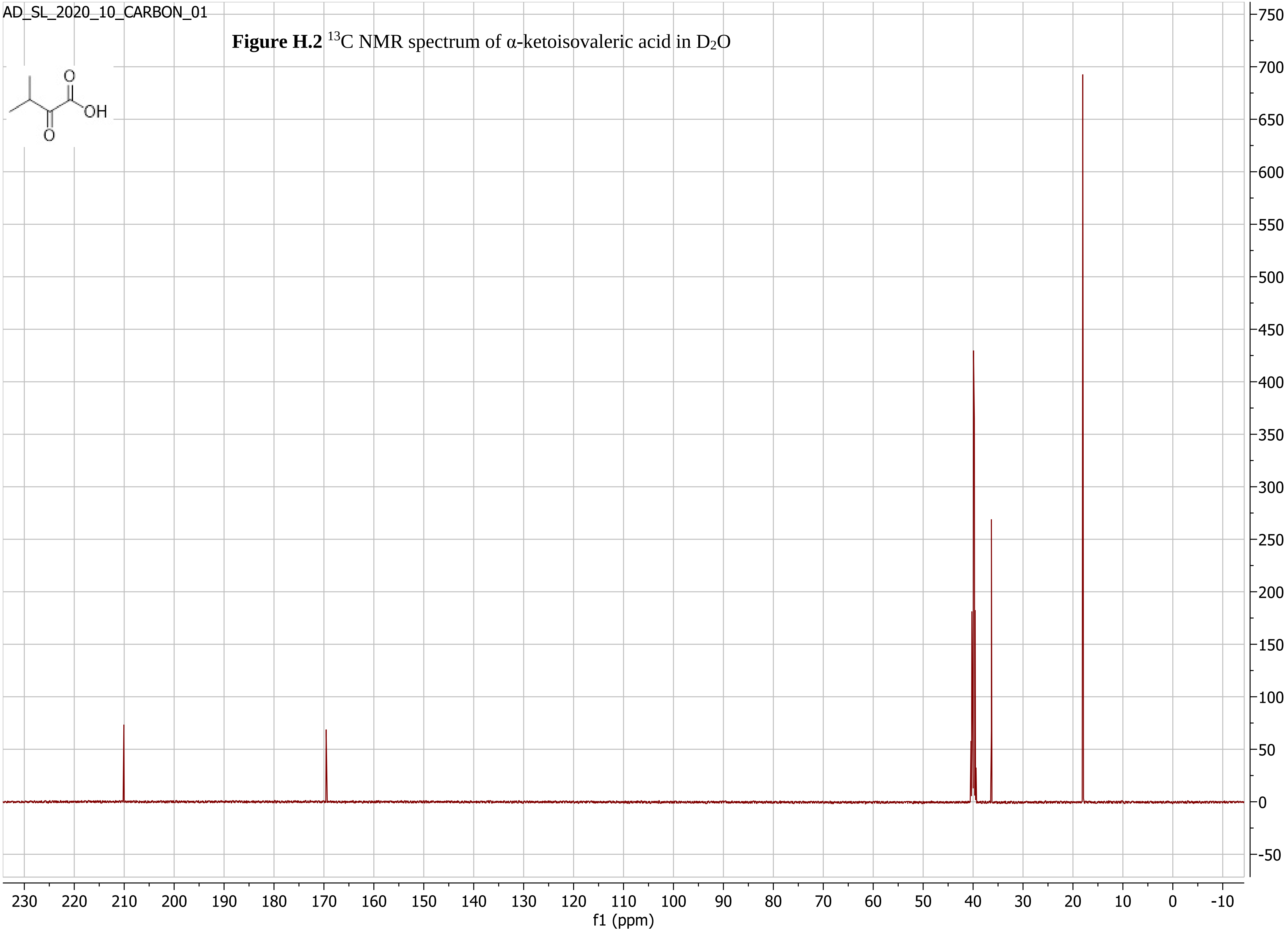

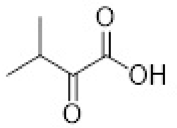

Figure H.3 HSQC NMR spectrum of α-ketoisovaleric acid in D_2O

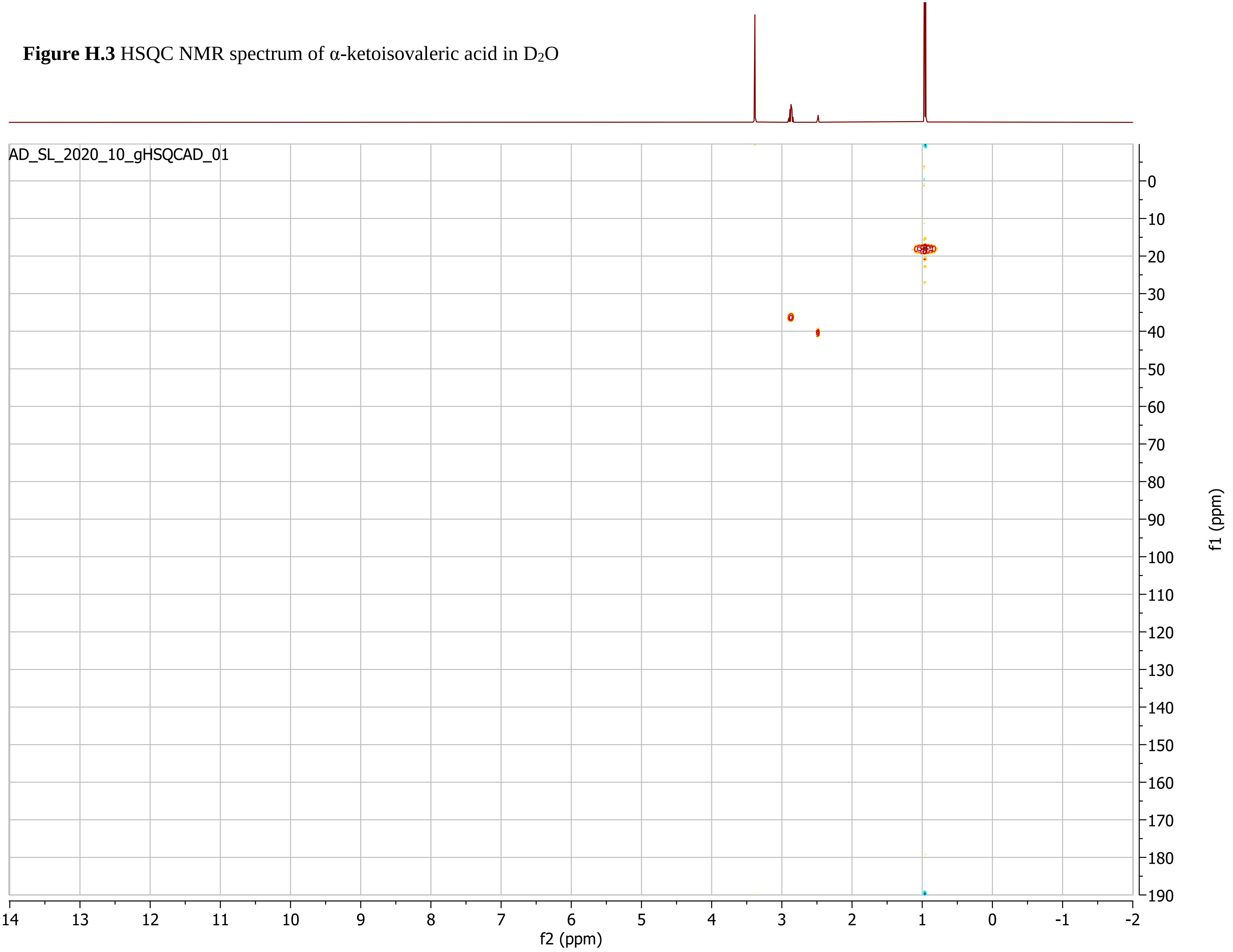

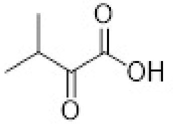

Figure H.4 HMBC NMR spectrum of α-ketoisovaleric acid in D_2O

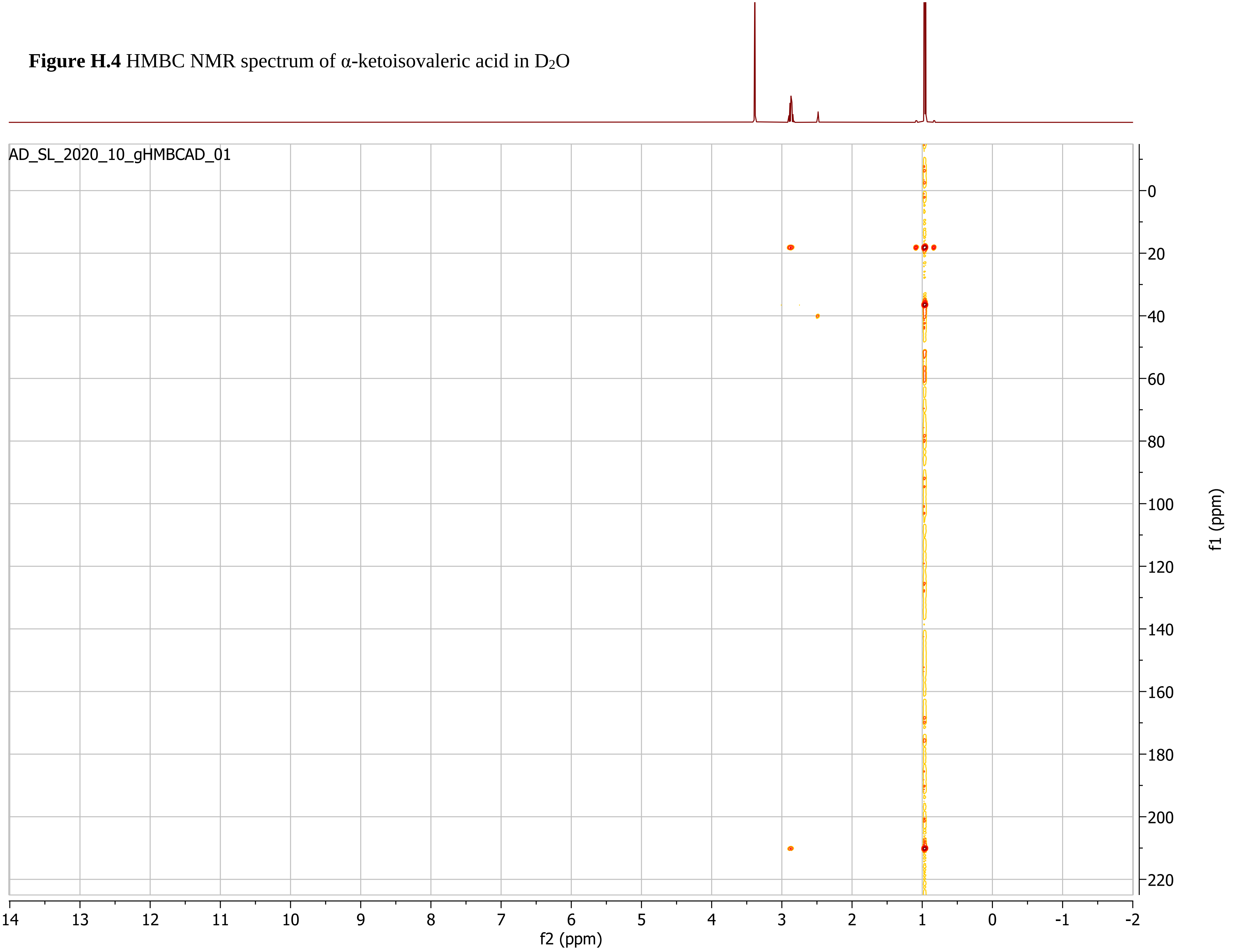

Figure H.5 ^{1}H-^{1}H COSY NMR spectrum of α-ketoisovaleric acid in D_2O

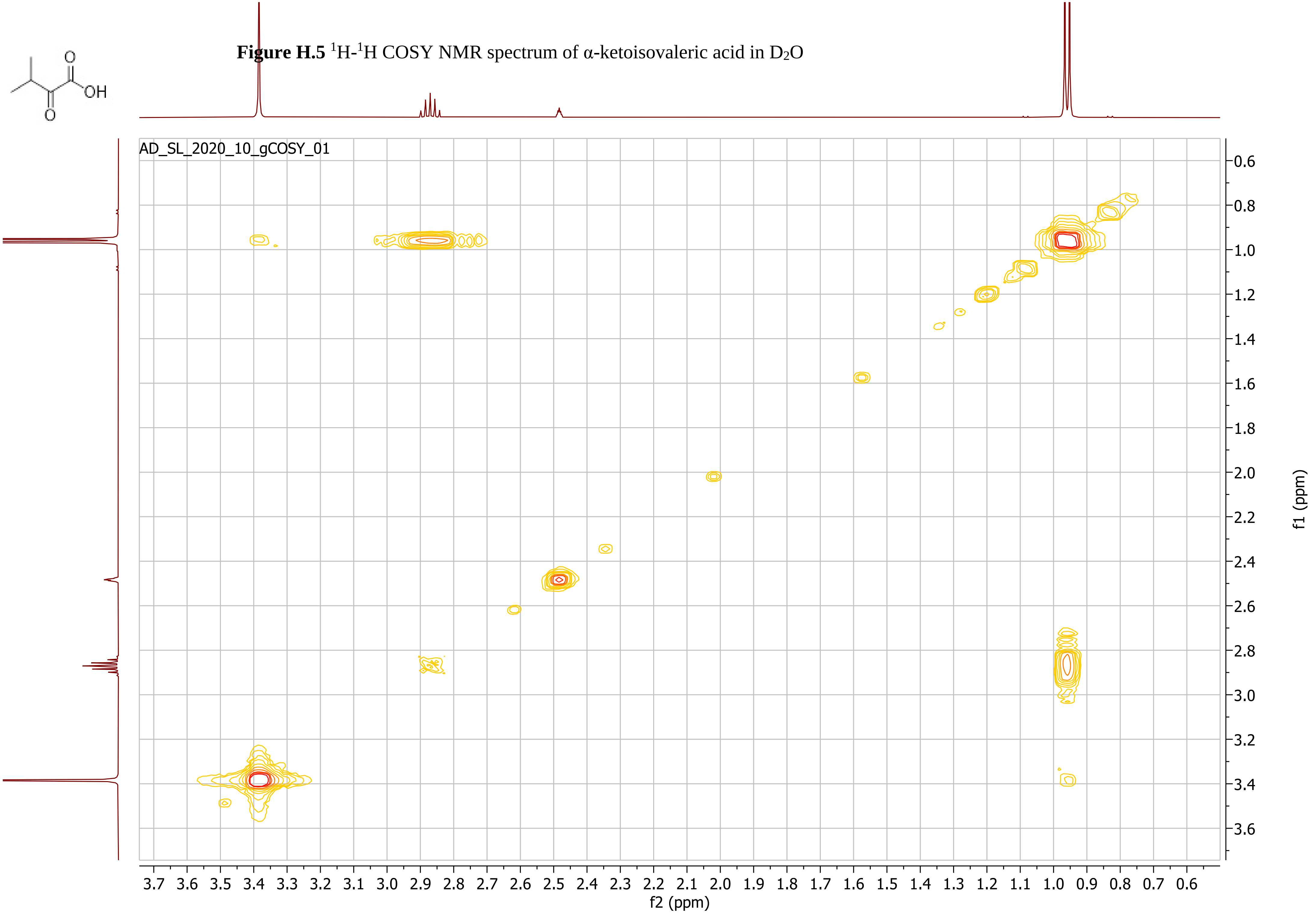

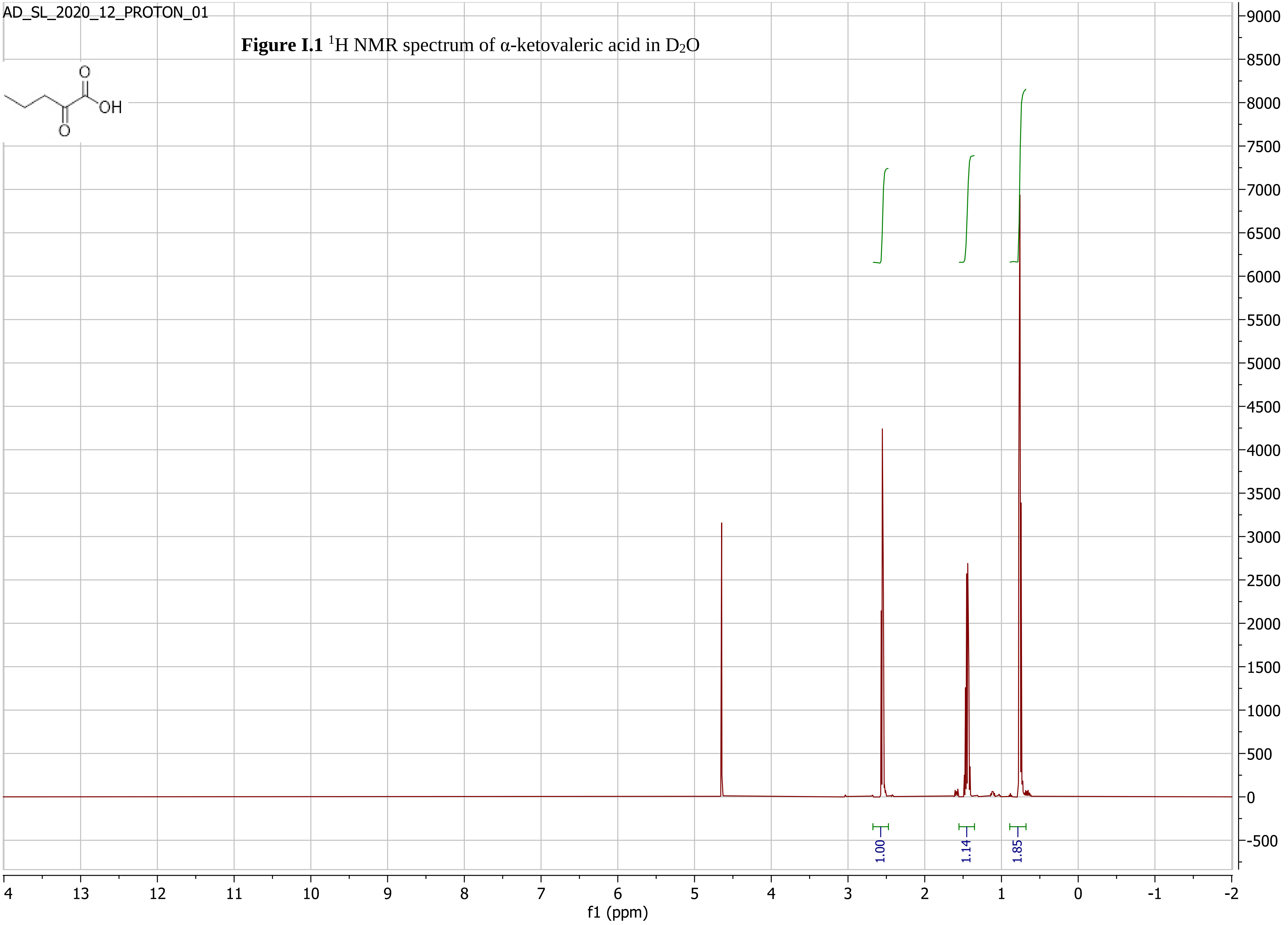

Figure I.1 ^{1}H NMR spectrum of α-ketovaleric acid in D_2O

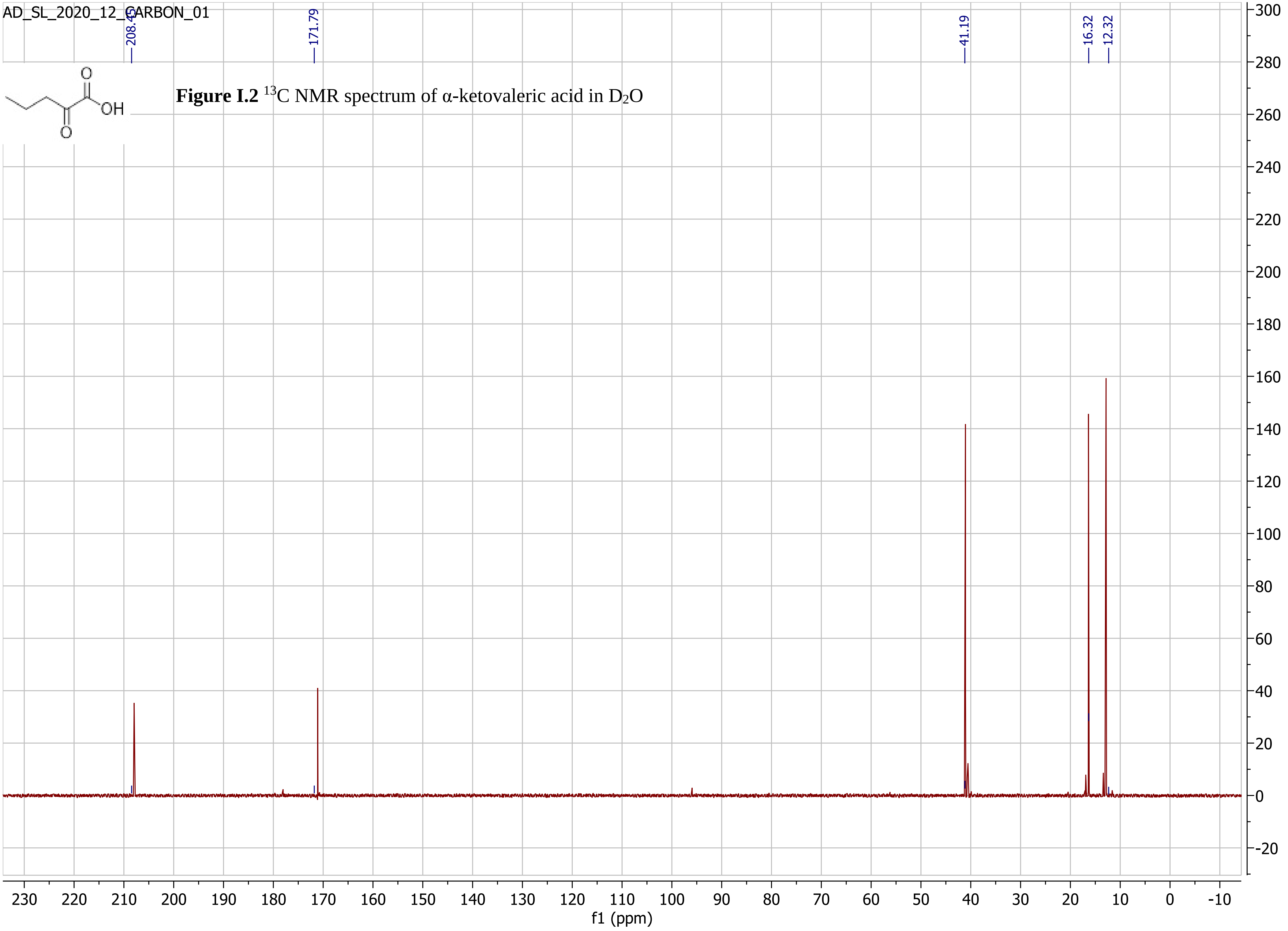

Figure I.2 ^{13}C NMR spectrum of α-ketovaleric acid in D_2O

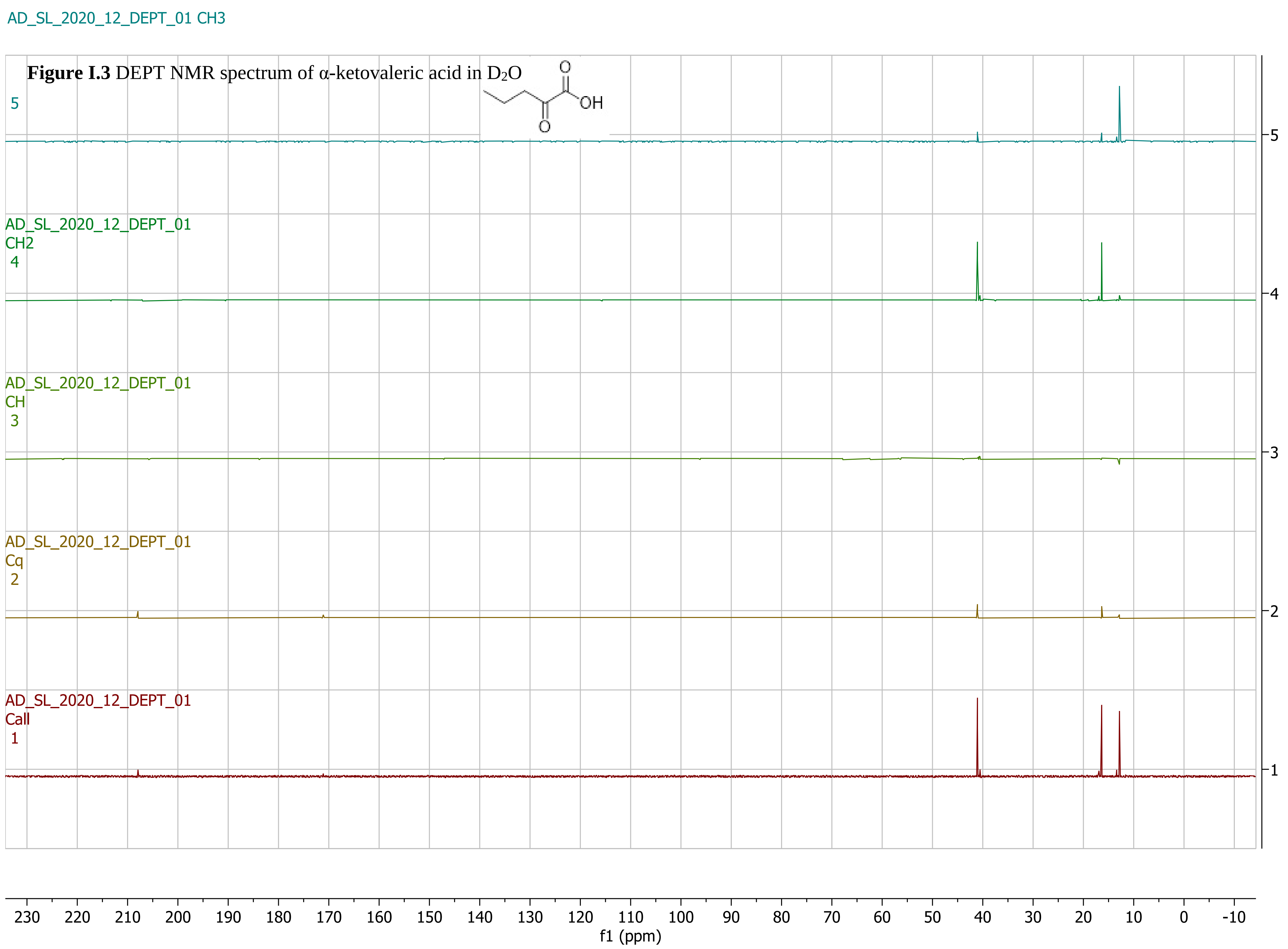

Figure I.3 DEPT NMR spectrum of α-ketovaleric acid in D_2O

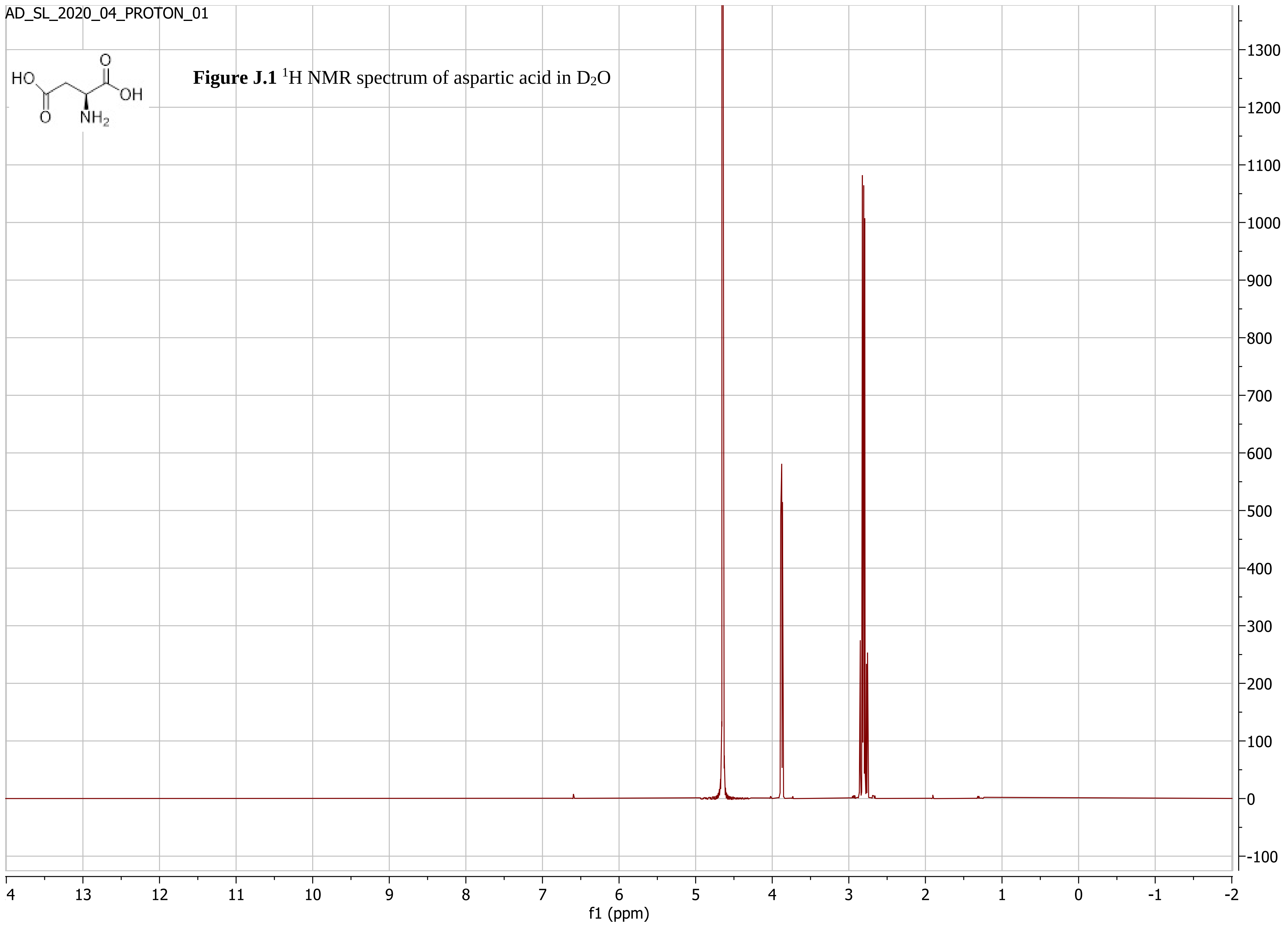

Figure J.1 ^{1}H NMR spectrum of aspartic acid in D_2O

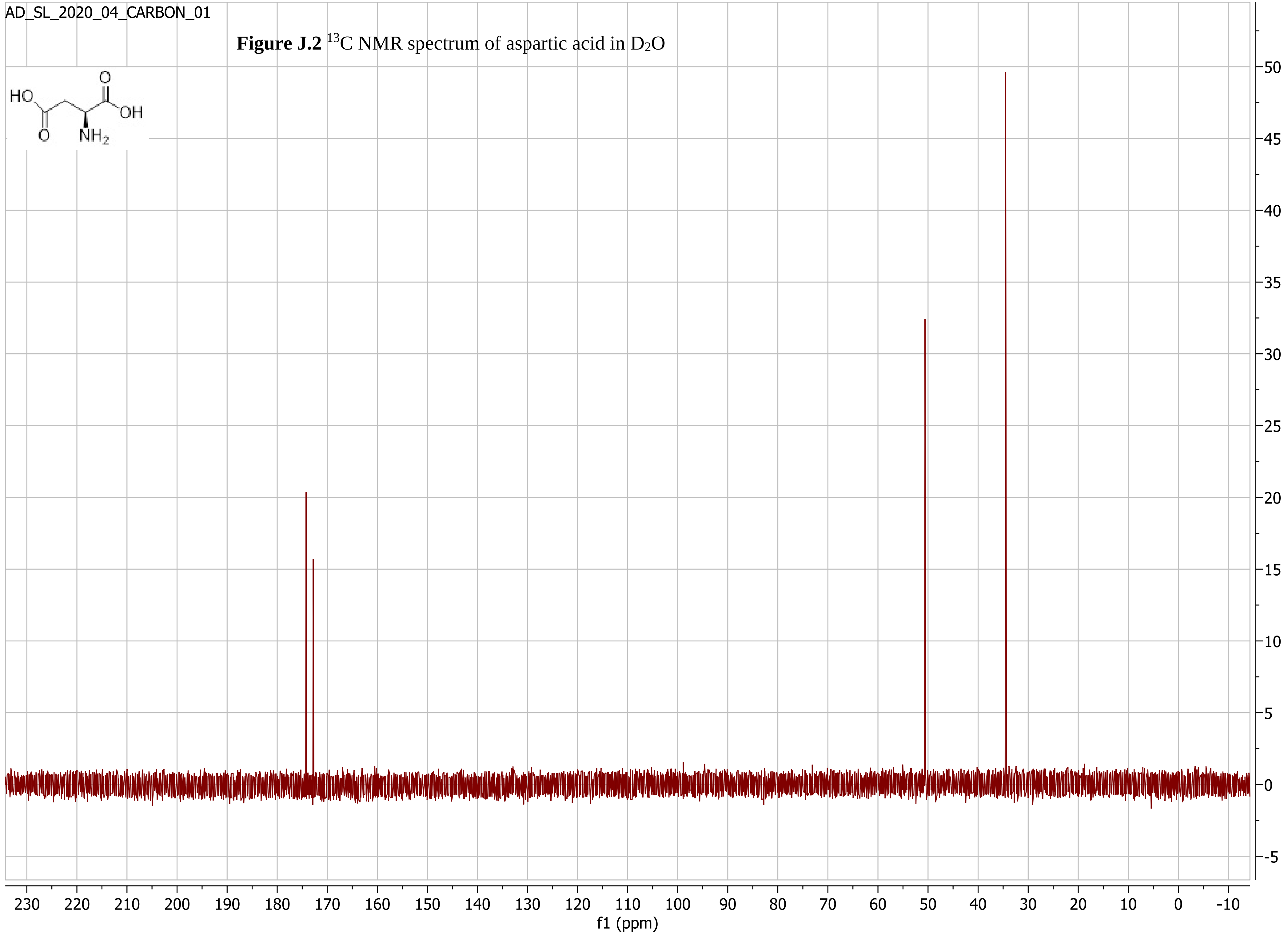

Figure J.2 ^{13}C NMR spectrum of aspartic acid in D_2O

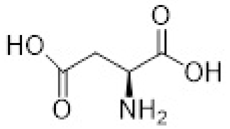

Figure J.3 HMBC NMR spectrum of aspartic acid in D_2O

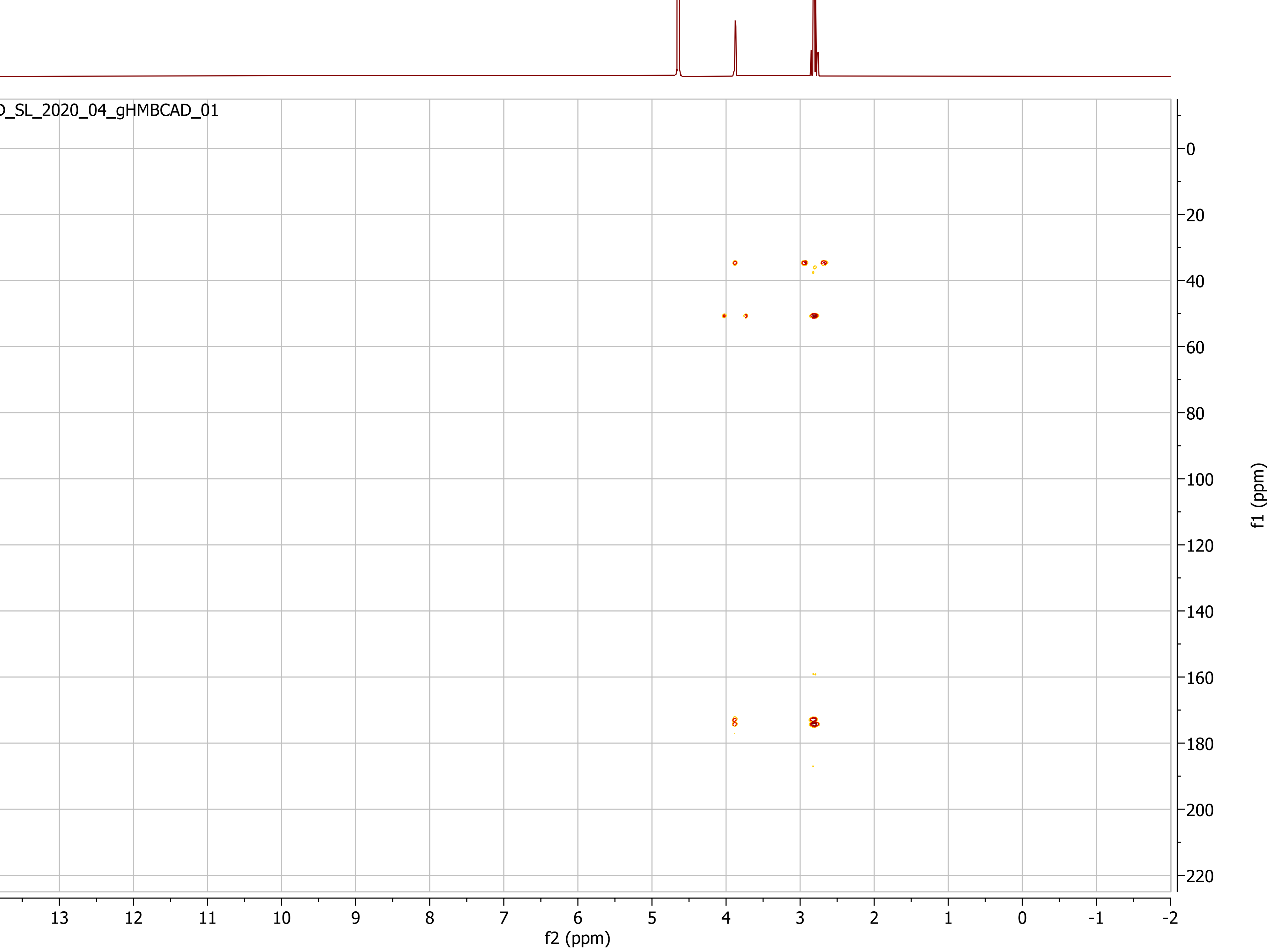

Figure K.1 ^1H NMR spectrum of putrescine in D_2O

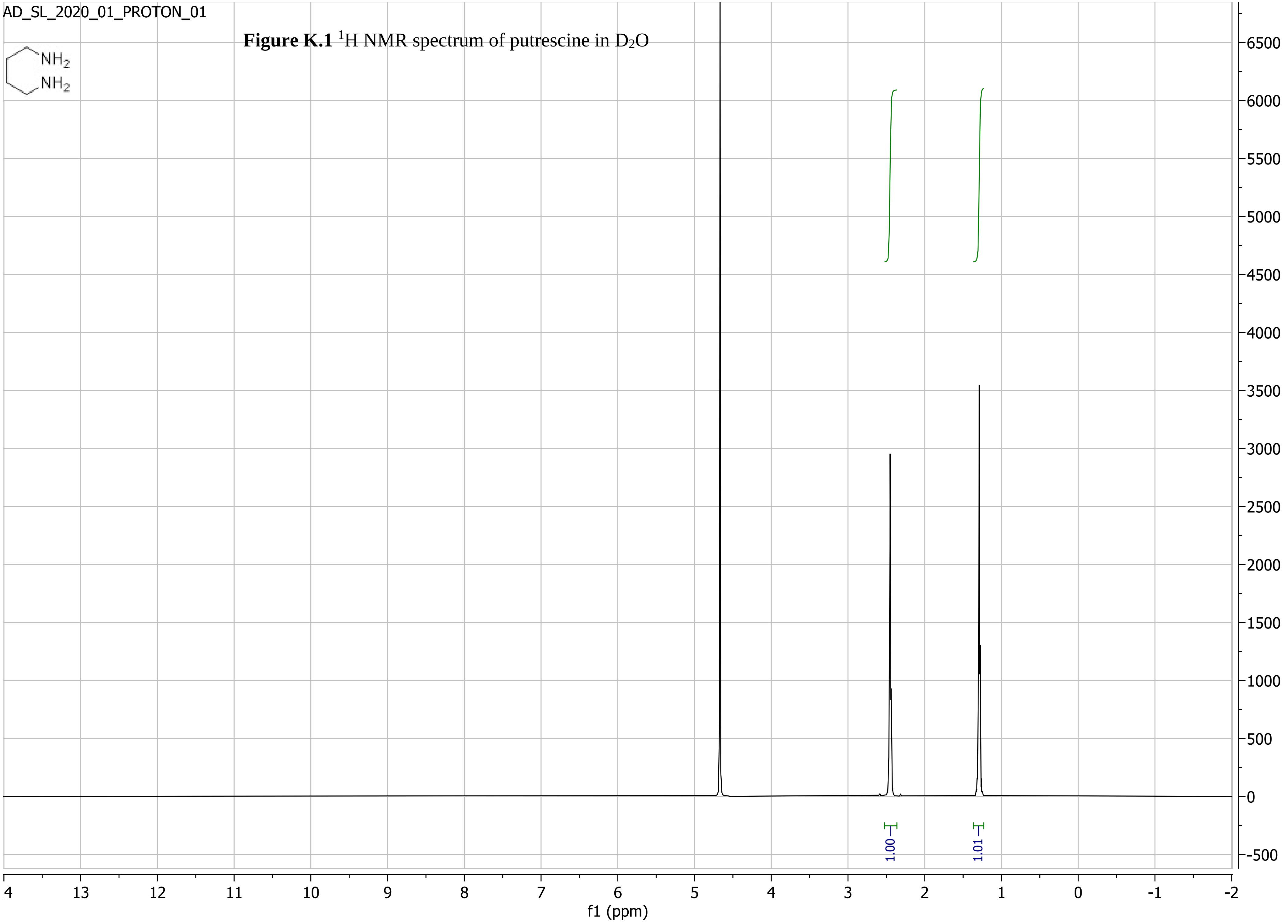

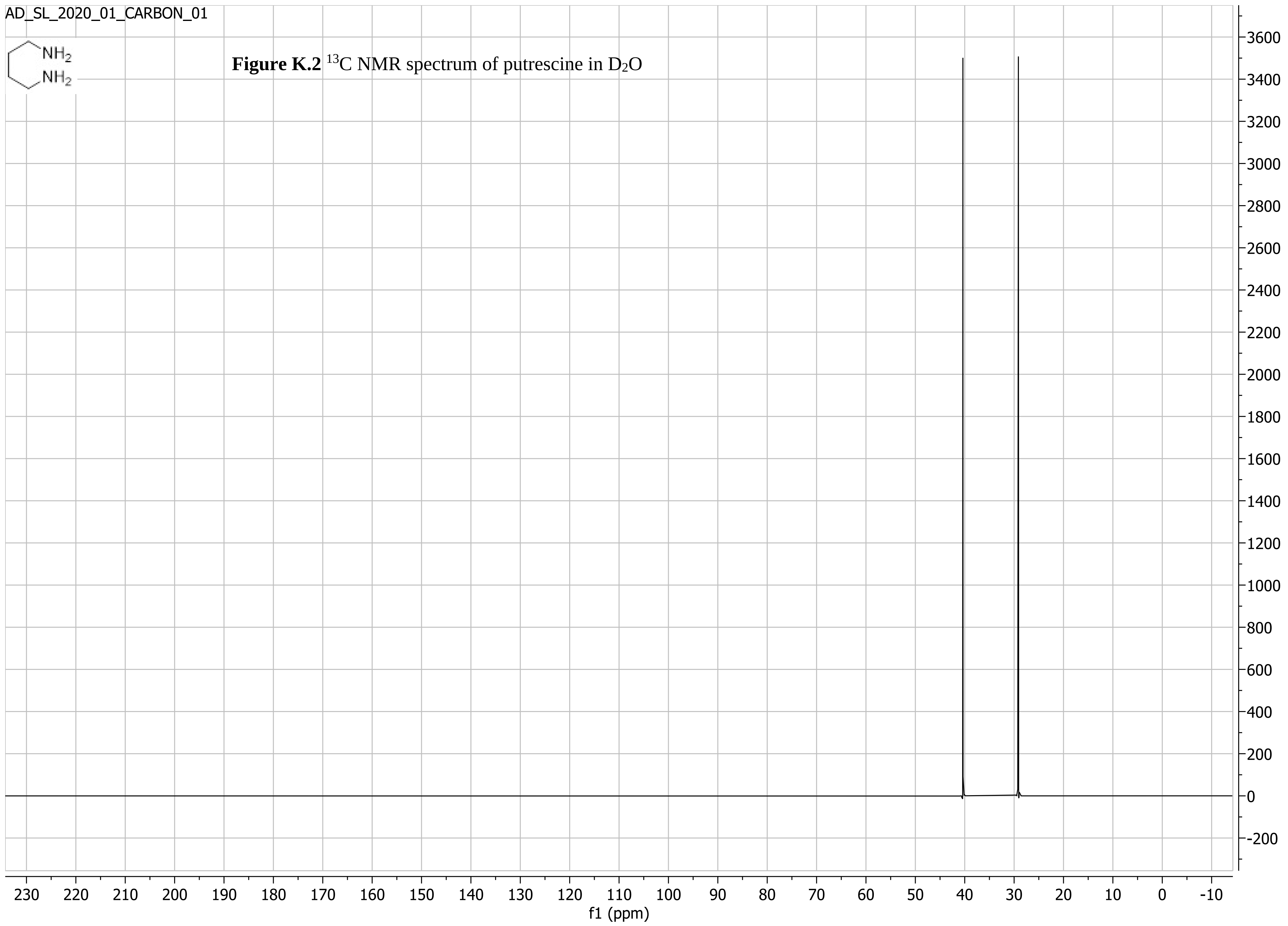

Figure K.2 ^{13}C NMR spectrum of putrescine in D_2O

Figure K.3 HSQC NMR spectrum of putrescine in D_2O

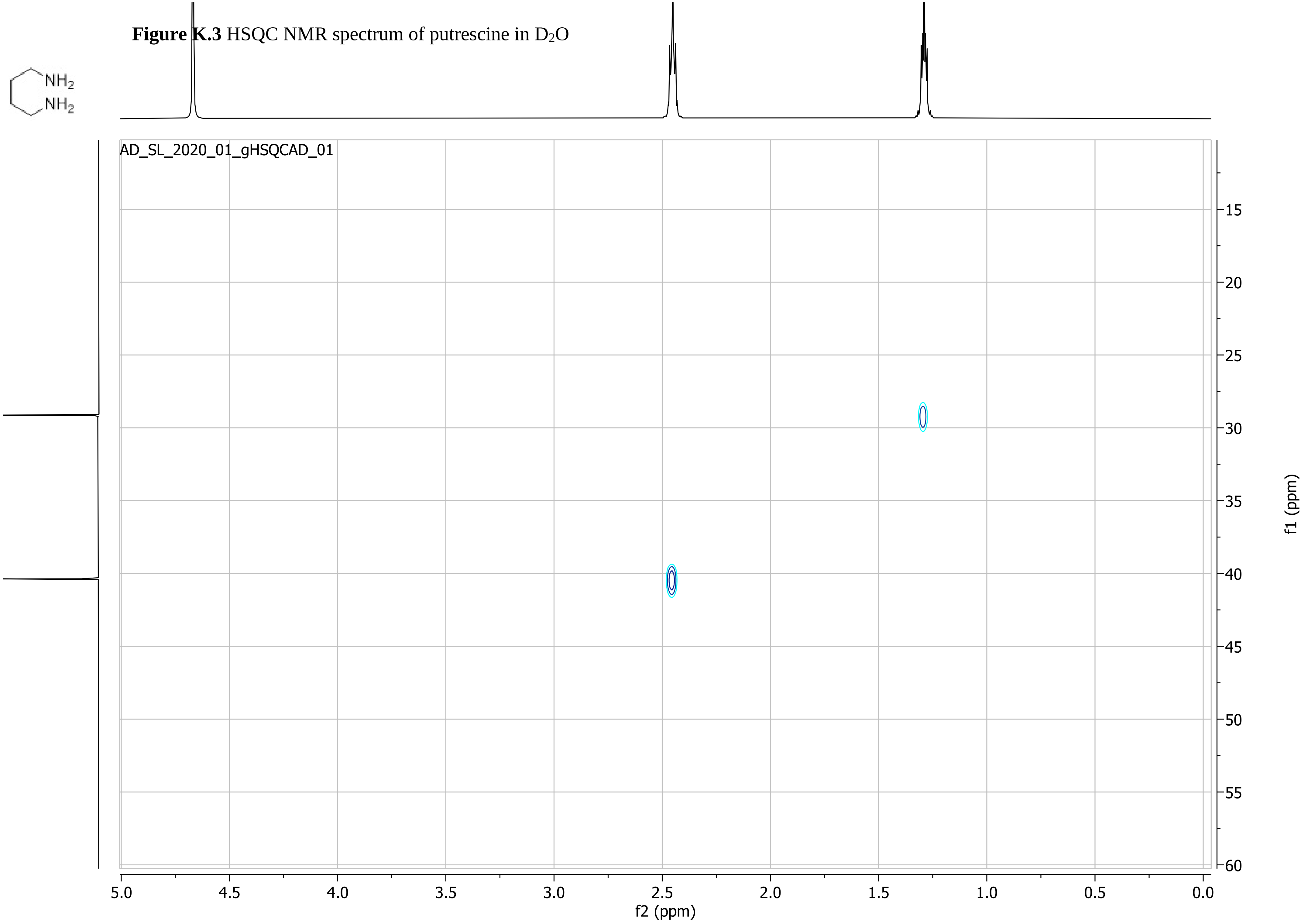

Figure K.4 HMBC NMR spectrum of putrescine in D_2O

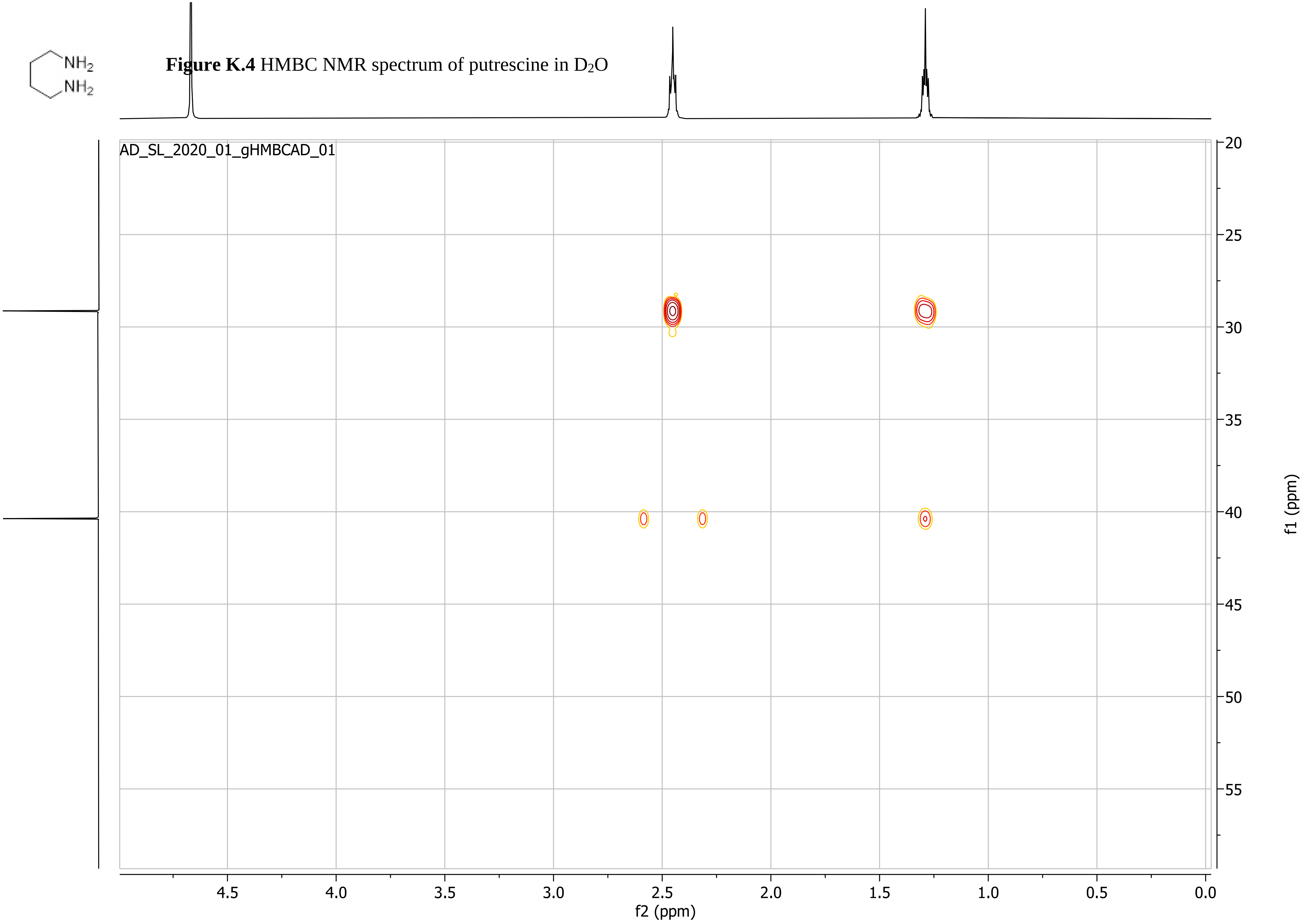

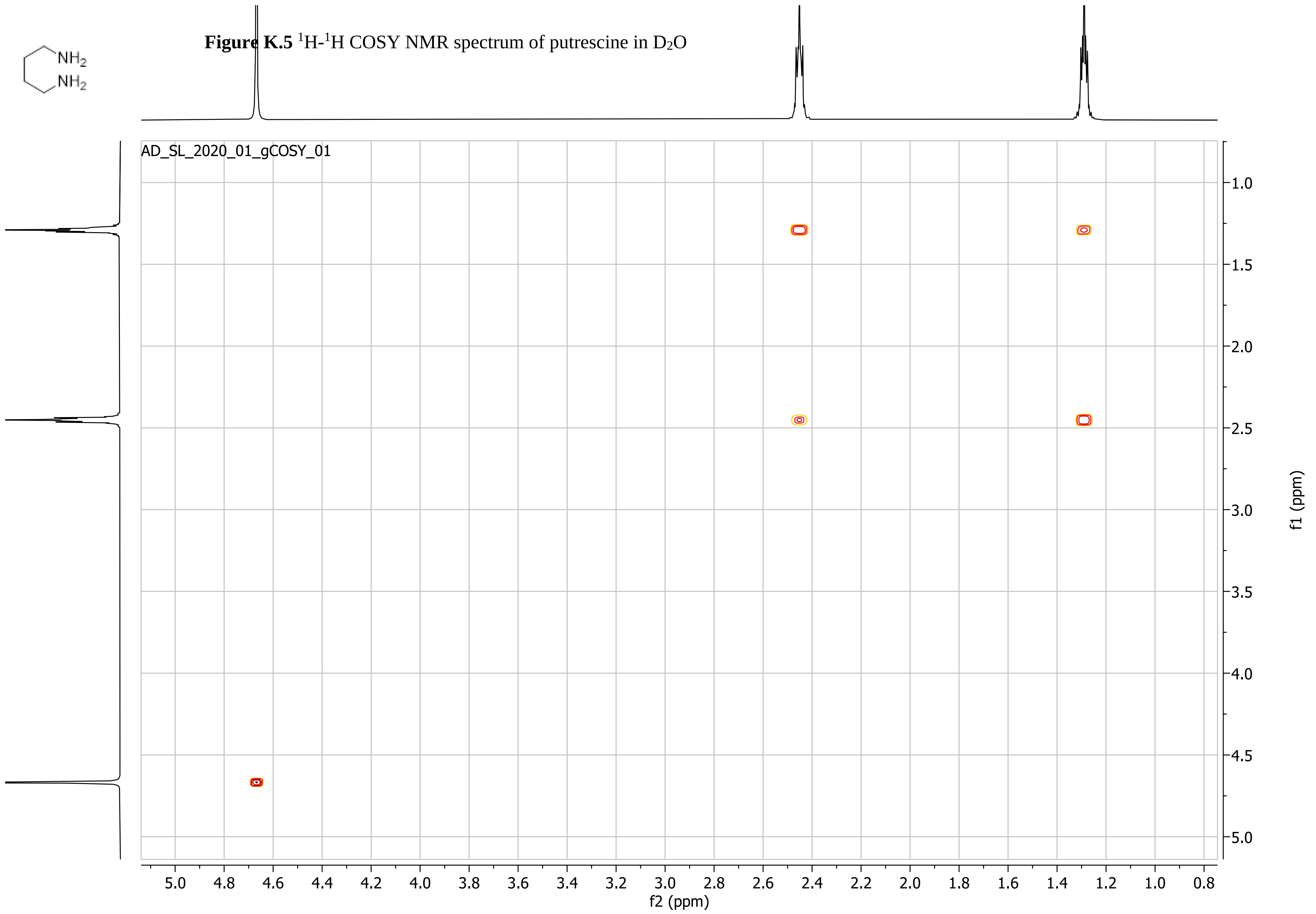

Figure K.5 ^{1}H-^{1}H COSY NMR spectrum of putrescine in D_2O

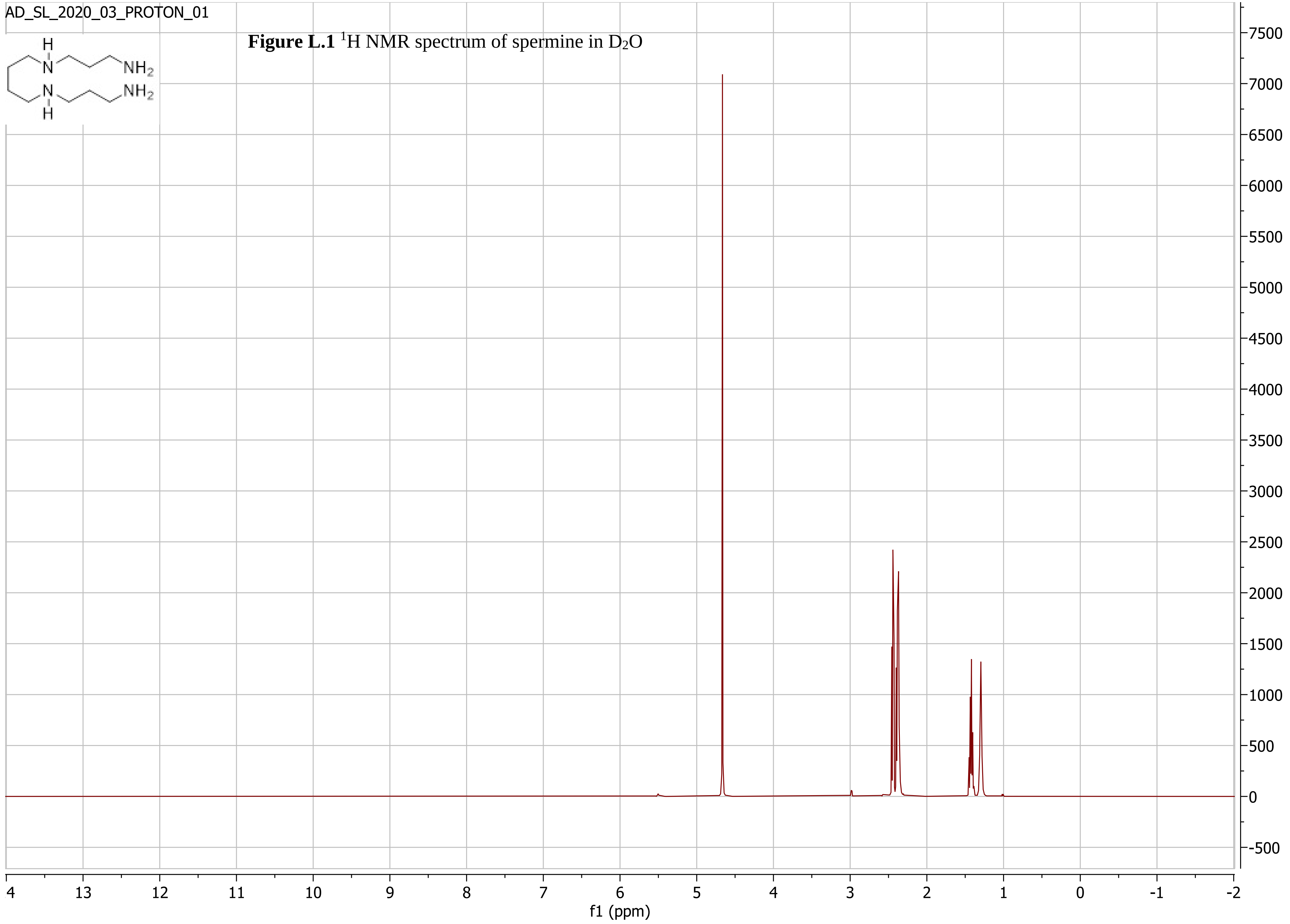

Figure L.1 ^{1}H NMR spectrum of spermine in D_2O

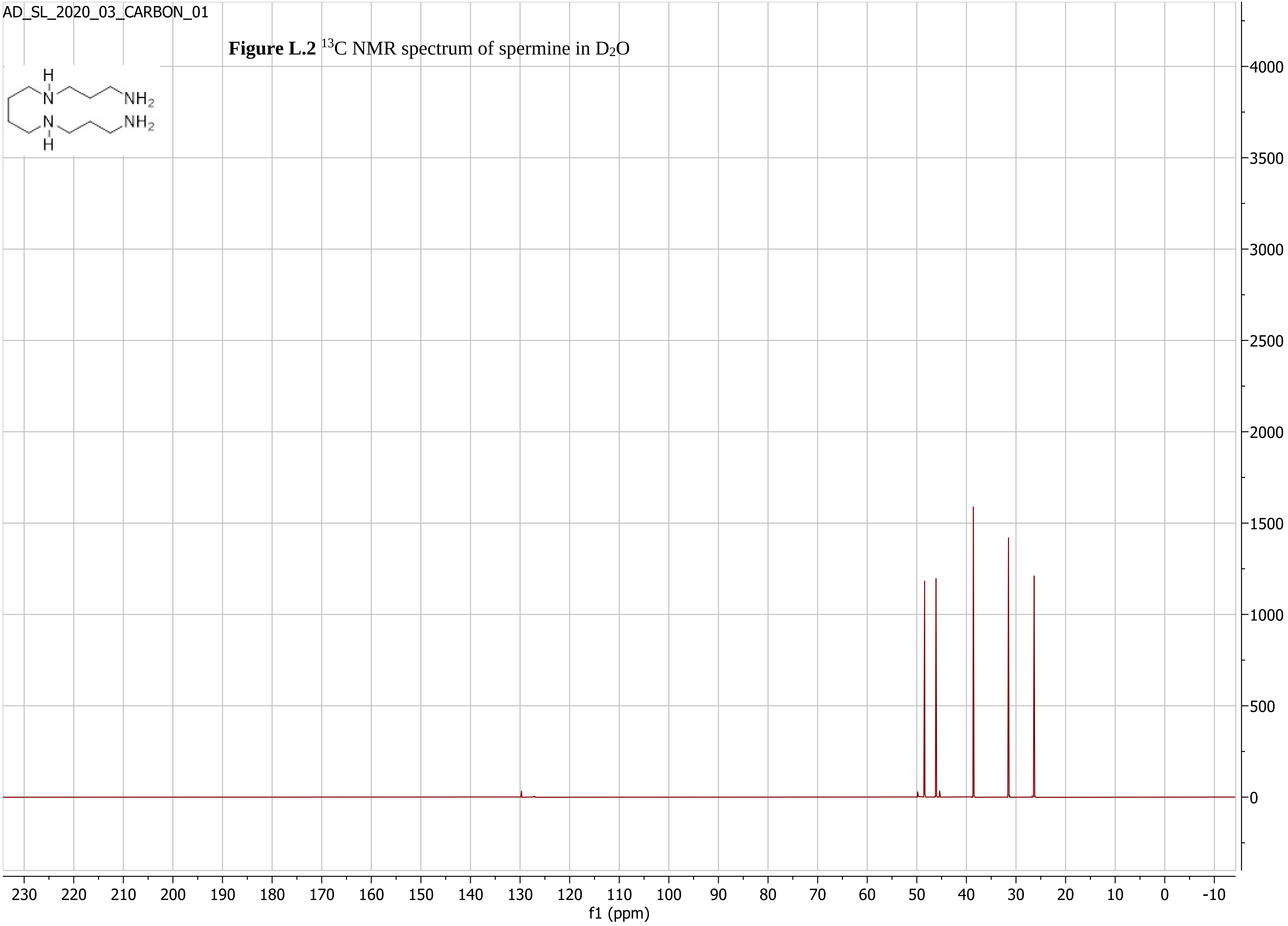

Figure L.2 ^{13}C NMR spectrum of spermine in D_2O

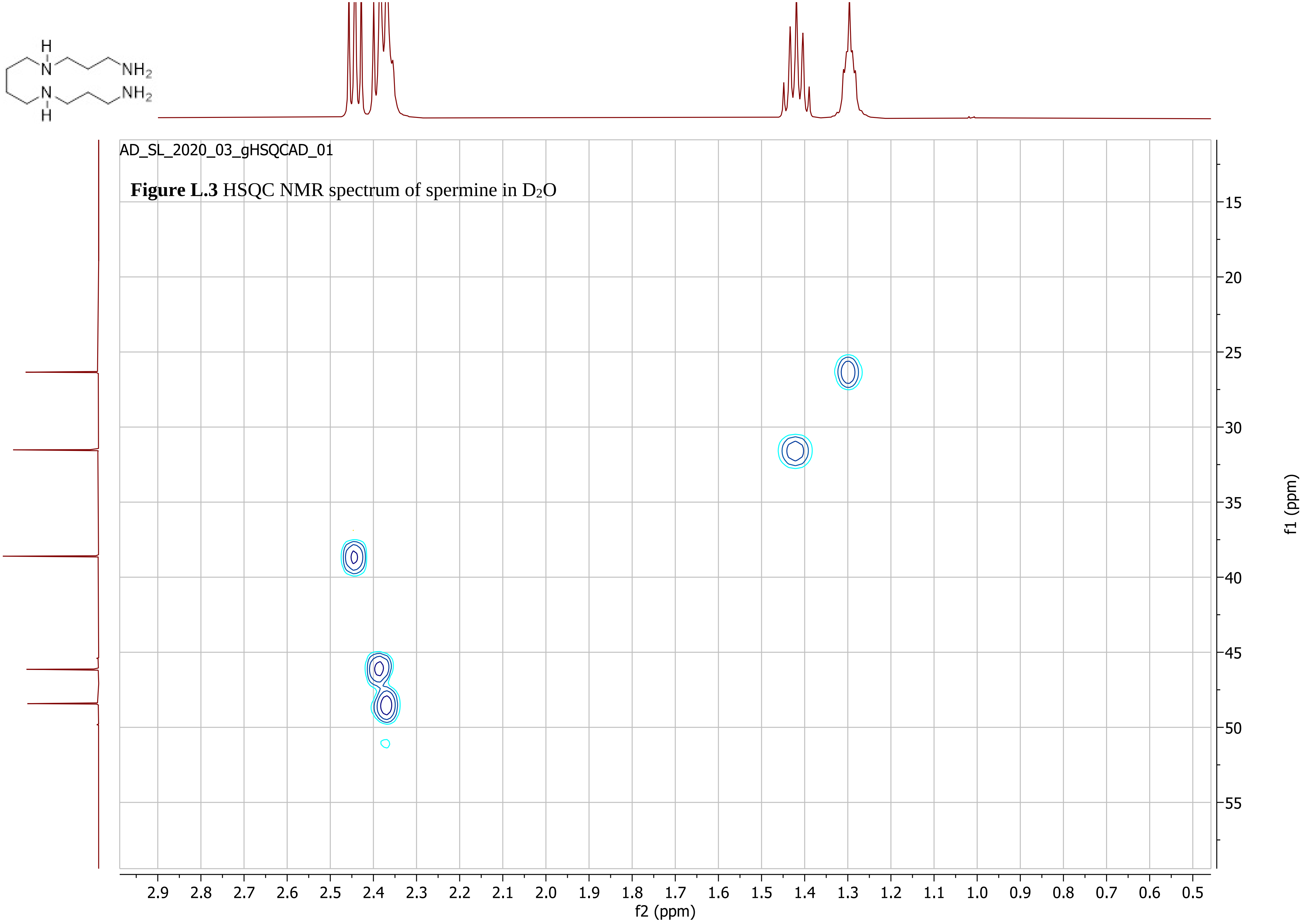

Figure L.3 HSQC NMR spectrum of spermine in D_2O

Figure L.4 HMBC NMR spectrum of spermine in D_2O

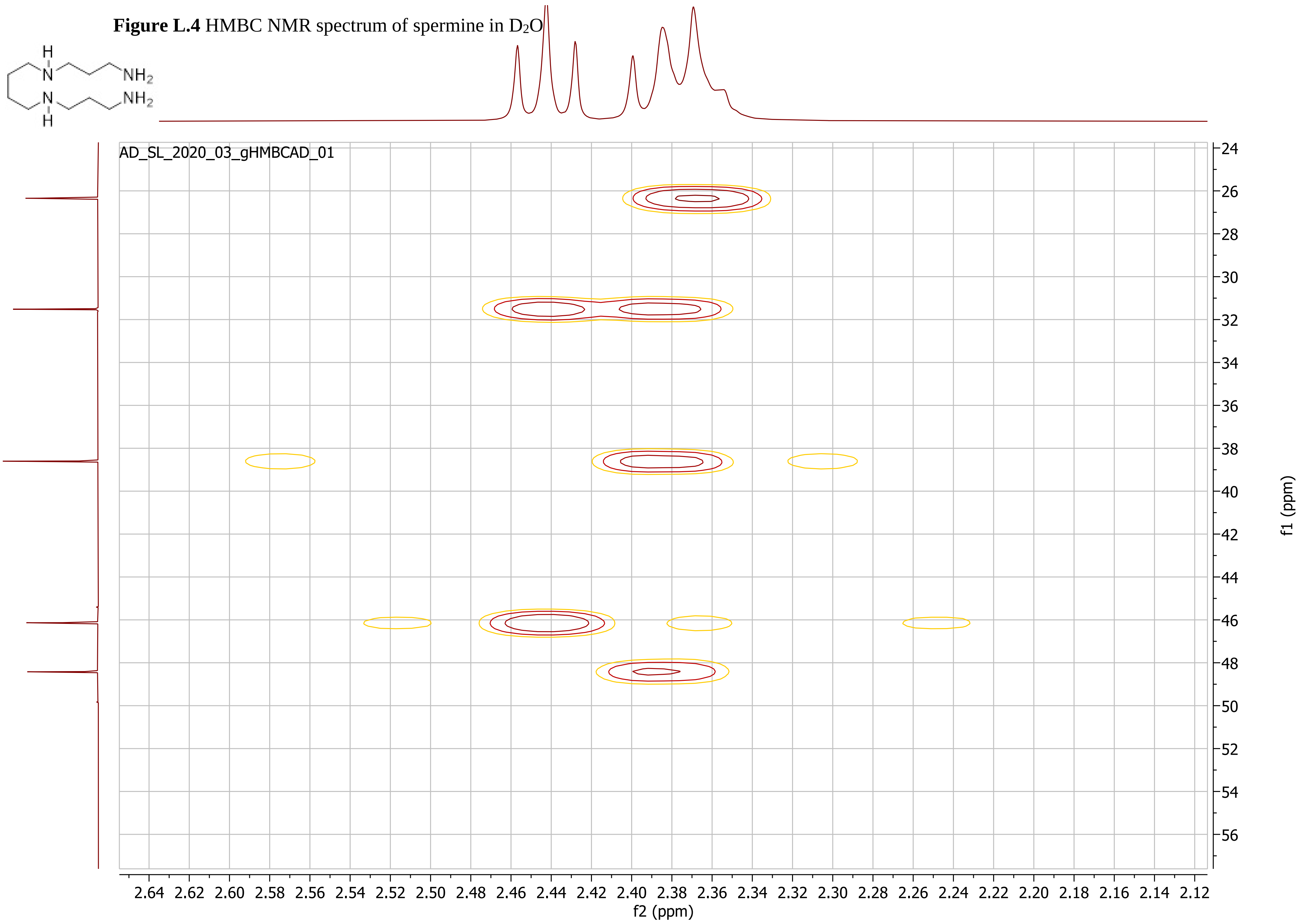

Figure L.5 ^{1}H-^{1}H COSY NMR spectrum of spermine in D_2O

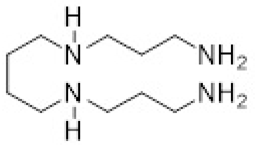

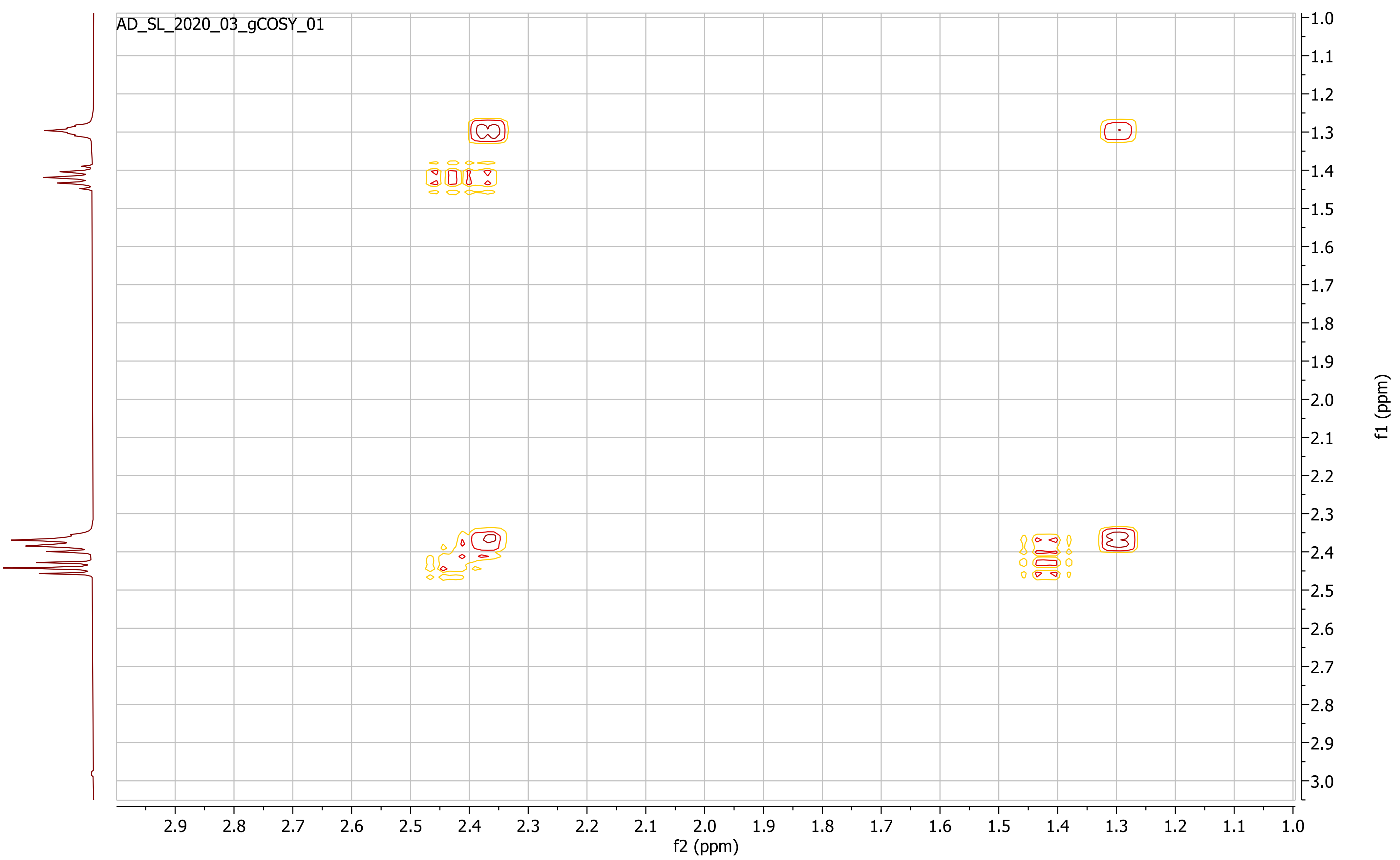

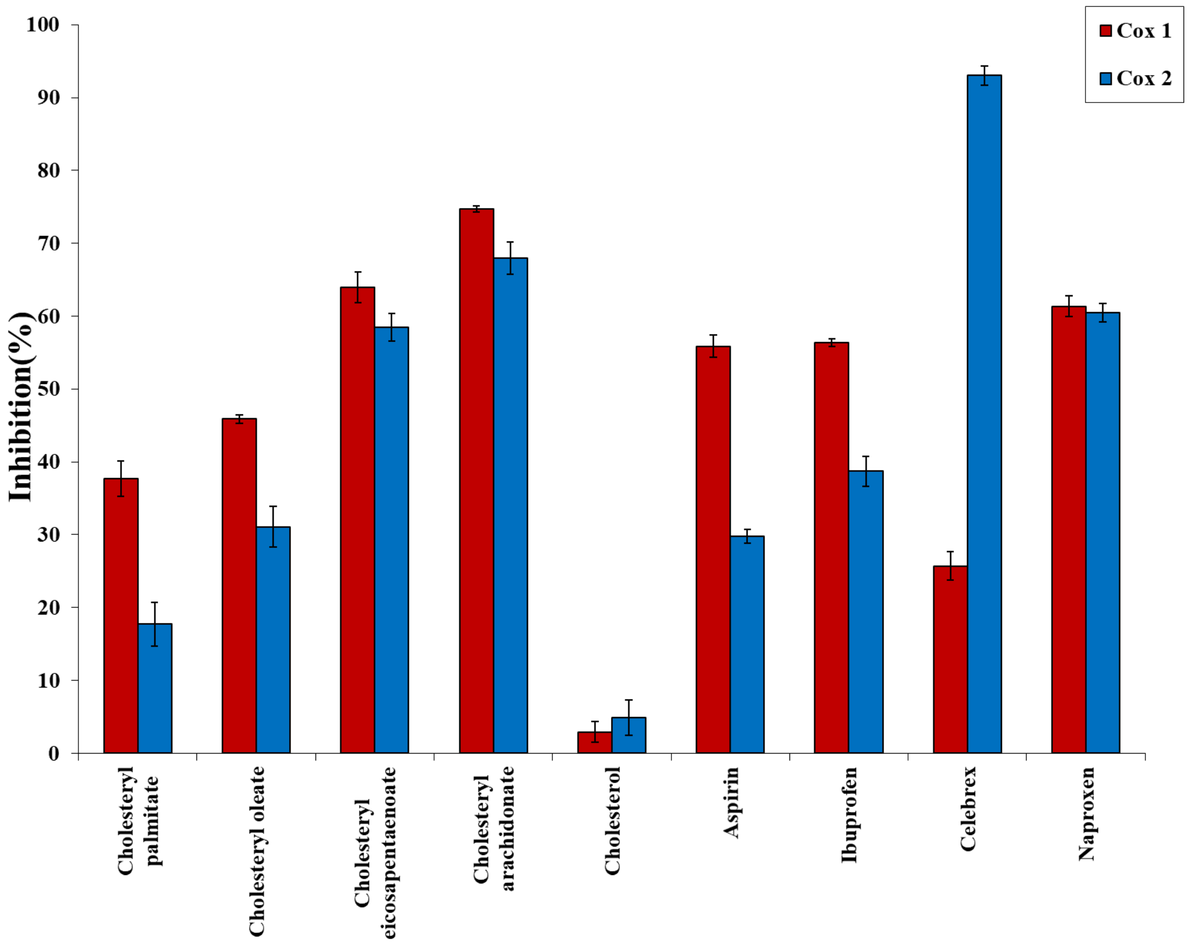

Figure M Cyclooxygenase enzyme (COX-1 and -2) inhibitory activity of positive control NSAIDs aspirin, ibuprofen, naproxen and Celebrex® were tested at 108, 12, 15 and 1 µg/mL, respectively. Sterol's cholesteryl palmitate, cholesteryl oleate, cholesteryl eicosapentaenoate, cholesteryl arachidonate and cholesterol were tested at 25 µg/mL concentration. Vertical bars represent the standard deviation of each data point (n=4). The varying concentrations of positive controls and compounds used were to yield comparable inhibitory activity values between 0–100%.

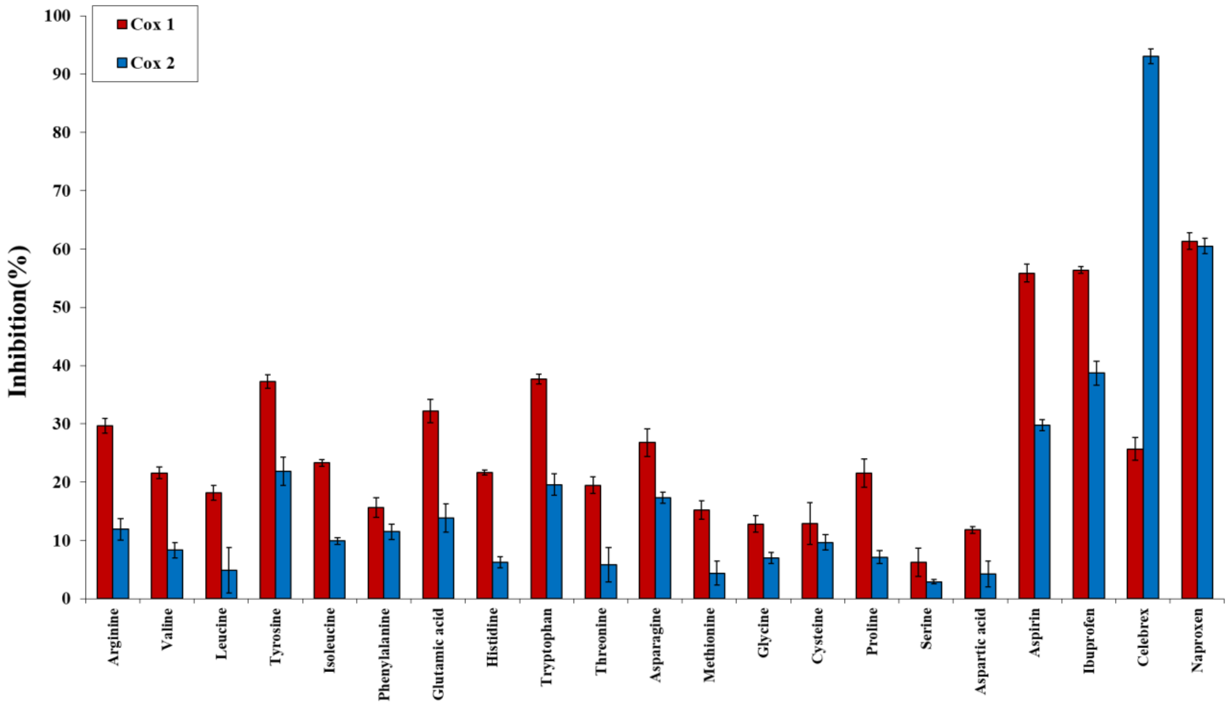

Figure N Cyclooxygenase enzyme (COX-1 and -2) inhibitory activity of positive control NSAIDs aspirin, ibuprofen, naproxen and Celebrex® were tested at 108, 12, 15 and 1 µg/mL, respectively. Amino acids arginine, valine, leucine, tyrosine, isoleucine, phenylalanine, glutamic acid, histidine, tryptophan, threonine, asparagine, methionine, glycine, cysteine, proline, serine, and aspartic acid were tested at 25 µg/mL. Vertical bars represent the standard deviation of each data point (n=4). The varying concentrations of positive controls and compounds used were to yield comparable inhibitory activity values between 0–100%.

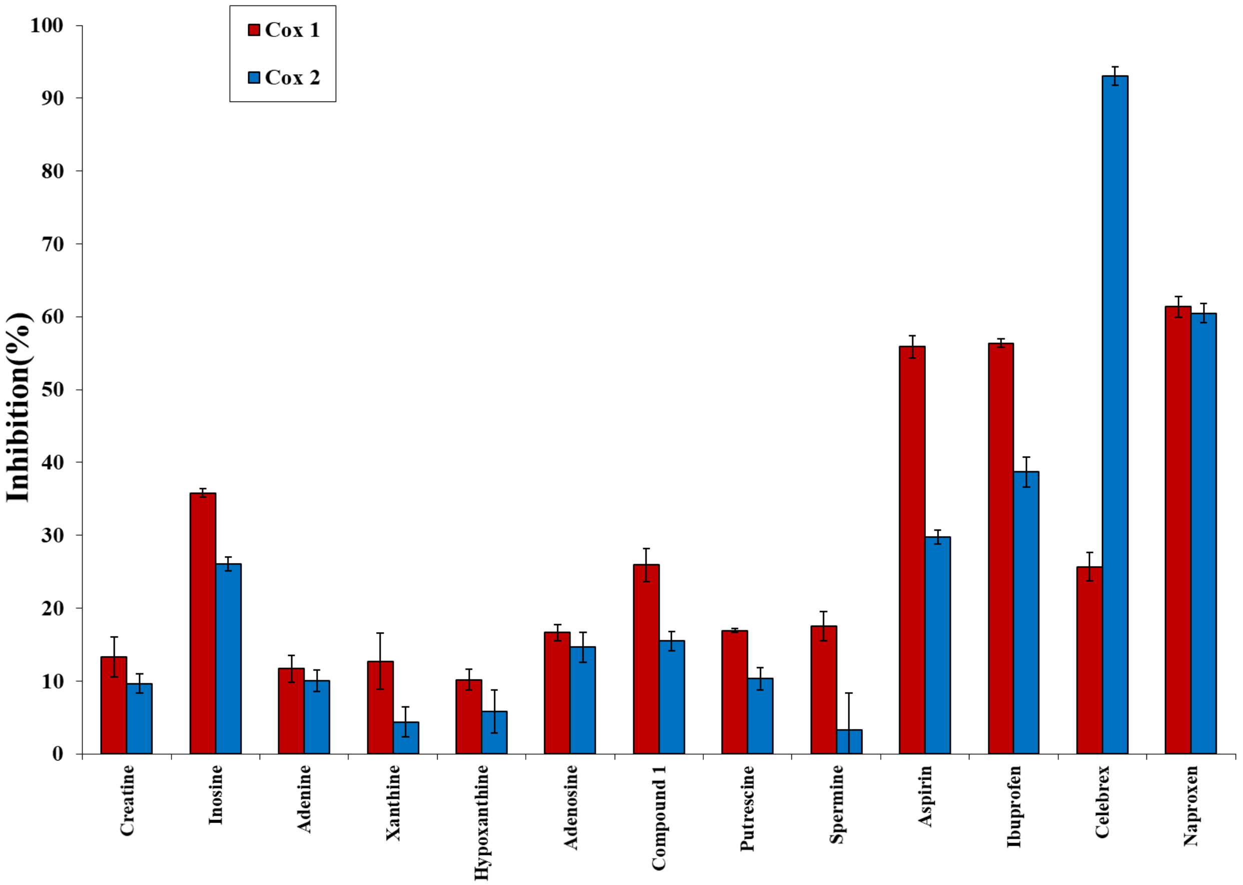

Figure O Cyclooxygenase enzyme (COX-1 and -2) inhibitory activity of positive control NSAIDs aspirin, ibuprofen, naproxen and Celebrex® were tested at 108, 12, 15 and 1 µg/mL respectively. Nitrogenous compounds creatine, inosine, adenine, xanthine, hypoxanthine, adenosine, compound 1, putrescine and spermine were tested at 25 µg/mL concentration. Vertical bars represent the standard deviation of each data point (n=4). The varying concentrations of positive controls and compounds used were to yield comparable inhibitory activity values between 0–100%.

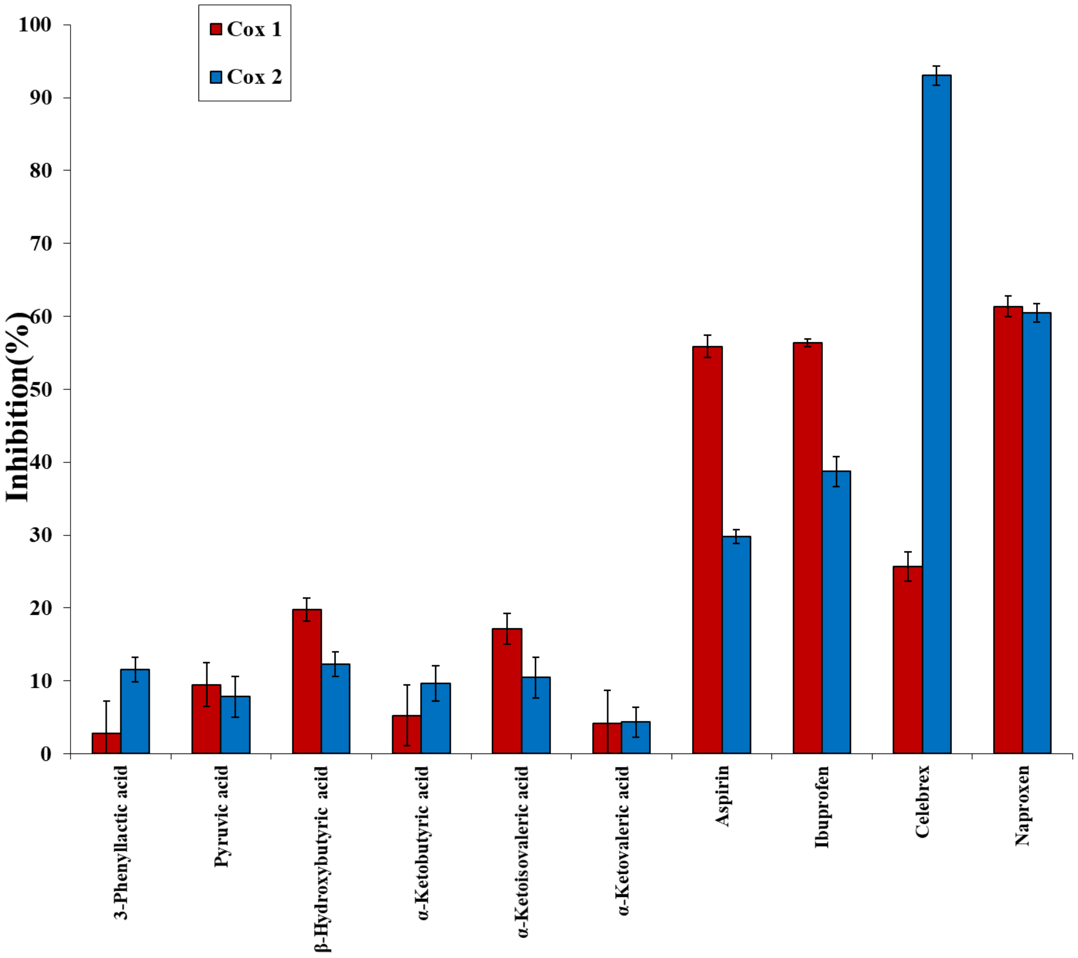

Figure P Cyclooxygenase enzyme (COX-1 and -2) inhibitory activity of positive control NSAIDs aspirin, ibuprofen, naproxen and Celebrex® were tested at 108, 12, 15 and 1 µg/mL, respectively. Organic acids α-ketovaleric acid, α-ketoisovaleric acid, α-ketobutyric acid, β-hydroxybutyric acid, pyruvic acid and 3-phenyllactic acid were tested at 25 µg/mL concentration. Vertical bars represent the standard deviation of each data point (n=4). The varying concentrations of positive controls and compounds used were to yield comparable inhibitory activity values between 0–100%.

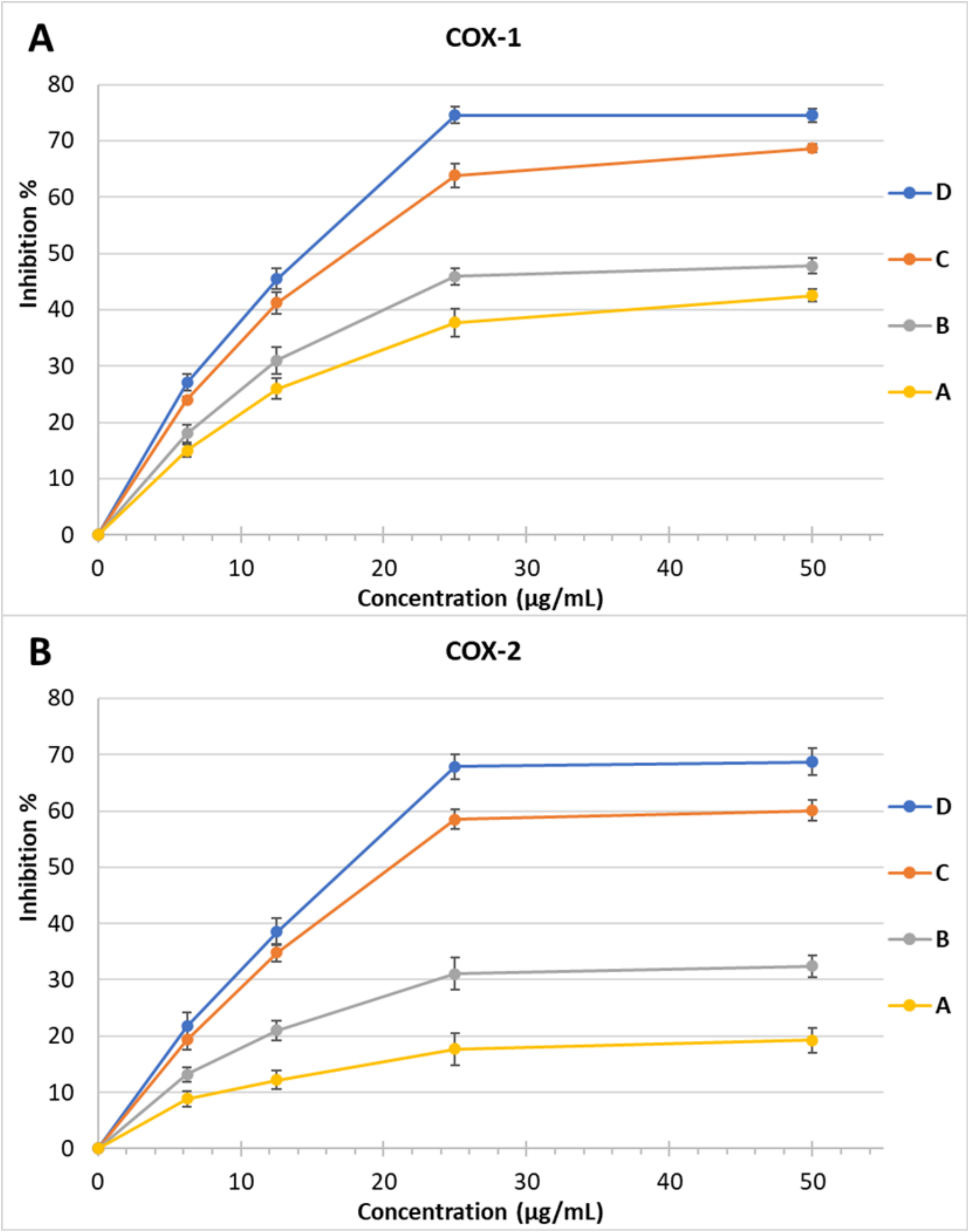

Figure Q (A) COX-1 enzyme inhibitory activities of cholesterol esters A (cholesteryl palmitate), B (cholesteryl oleate), C (cholesteryl eicosapentaenoate) and D (cholesteryl arachidonate) at 6.25, 12.5, 25 and 50 µg/mL, respectively. Values are expressed as mean ± SEM (n=4). (B) COX-2 enzyme inhibitory activities of cholesterol esters A, B, C and D at 6.25, 12.5, 25 and 50 µg/mL, respectively. Values are expressed as mean ± SEM (n=4).

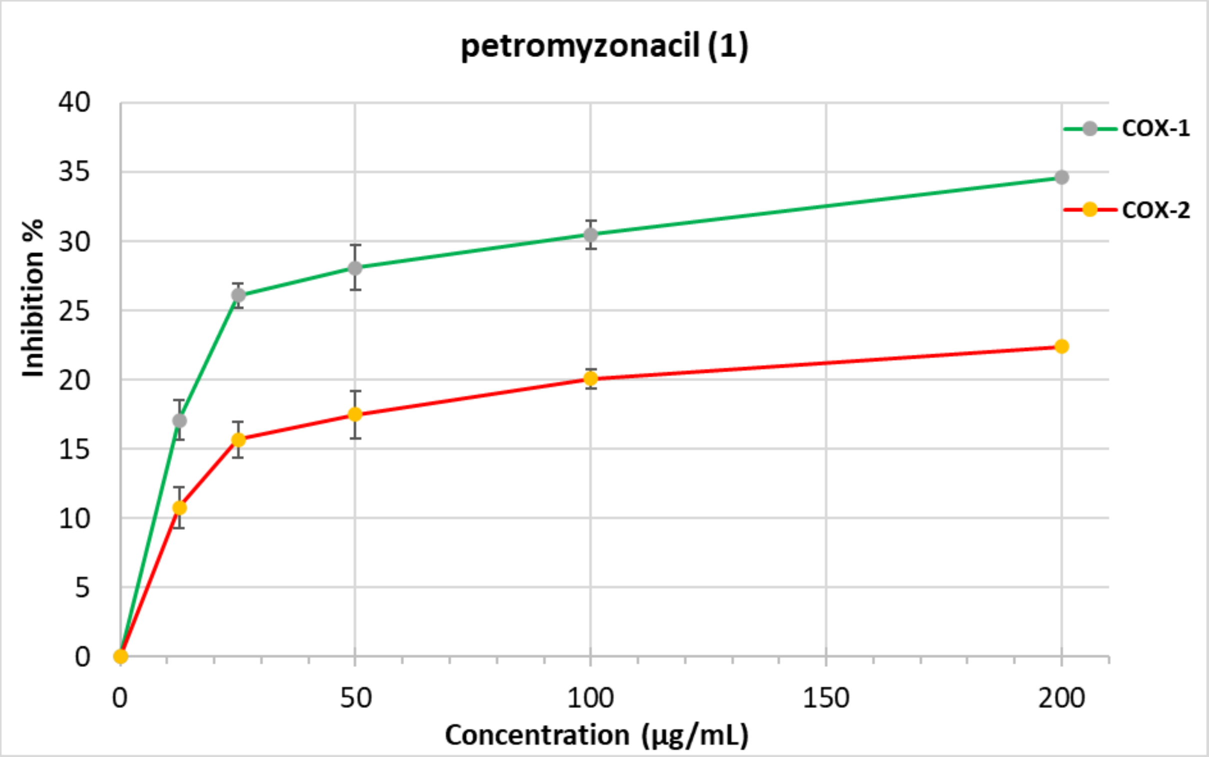

Figure R COX enzymes inhibitory activities of petromyzonacil (**1**) tested at 12.5, 25, 50,100 and 200 µg/mL, respectively. Positive controls aspirin, ibuprofen and naproxen exhibited COX-1 IC_{50} at 108, 15 and 12 µg/mL concentration, respectively. Values are expressed as mean ± SEM (n=4).

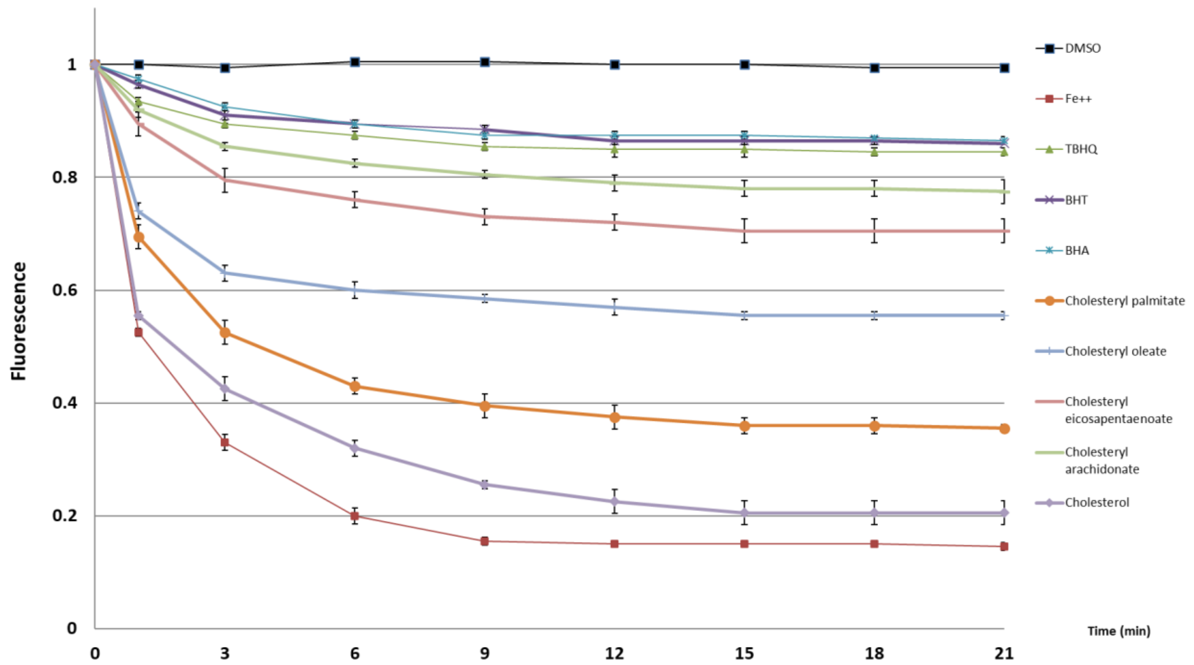

Figure S Inhibition of lipid peroxidation (LPO) by sterol's cholesteryl palmitate, cholesteryl oleate, cholesteryl eicosapentaenoate, cholesteryl arachidonate and cholesterol at 25 µg/mL concentration. Commercial antioxidants BHA, BHT and TBHQ used as positive controls at 1.8, 2.2 and 1.6 µg/mL, respectively. The varying concentrations used were to yield comparable activity profiles between 0-100 % by test compounds and positive controls alike. Vertical bars represent the standard deviation of each data point (n=2). The percent inhibition was calculated with respect to solvent control dimethyl sulfoxide.

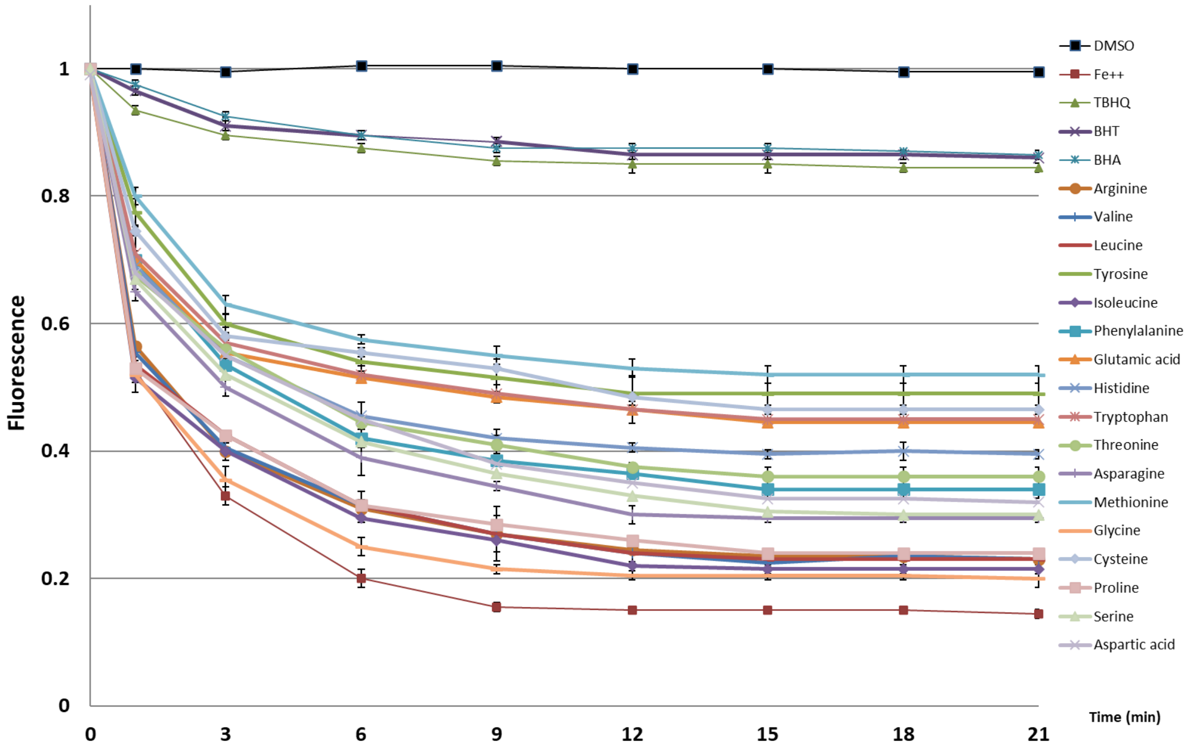

Figure T Inhibition of lipid peroxidation (LPO) by amino acids arginine, valine, leucine, tyrosine, isoleucine, phenylalanine, glutamic acid, histidine, tryptophan, threonine, asparagine, methionine, glycine, cysteine, proline, serine, and aspartic acid at 25 μg/mL concentration. Commercial antioxidants BHA, BHT and TBHQ used as positive controls at 1.8, 2.2 and 1.6 μg/mL, respectively. The varying concentrations used were to yield comparable activity profiles between 0-100 % by test compounds and positive controls alike. Vertical bars represent the standard deviation of each data point (n=2). The percent inhibition was calculated with respect to solvent control dimethyl sulfoxide.

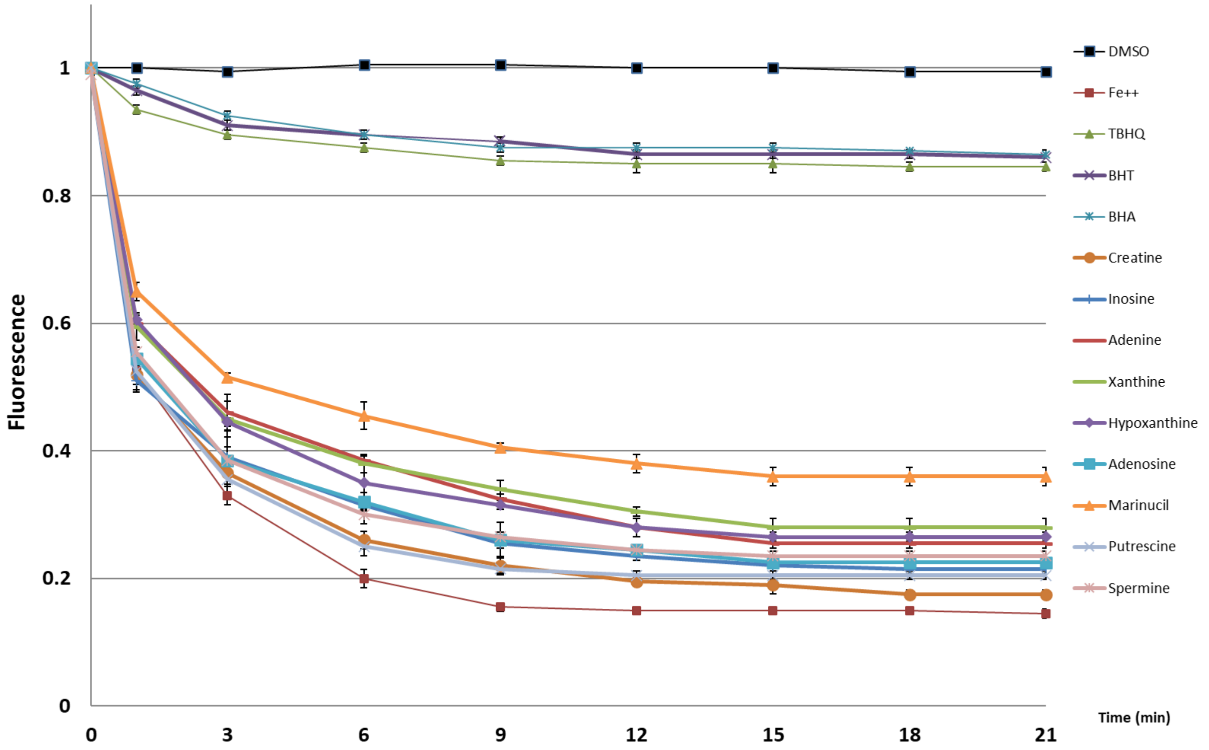

Figure U Inhibition of lipid peroxidation (LPO) by a nitrogenous compound's creatine, inosine, adenine, xanthine, hypoxanthine, adenosine, petromyzonacil (**1**), putrescine and spermine at 25 µg/mL concentration. Commercial antioxidants BHA, BHT and TBHQ used as positive controls at 1.8, 2.2 and 1.6 µg/mL, respectively. The varying concentrations used were to yield comparable activity profiles between 0-100 % by test compounds and positive controls alike. Vertical bars represent the standard deviation of each data point (n=2). The percent inhibition was calculated with respect to solvent control dimethyl sulfoxide.

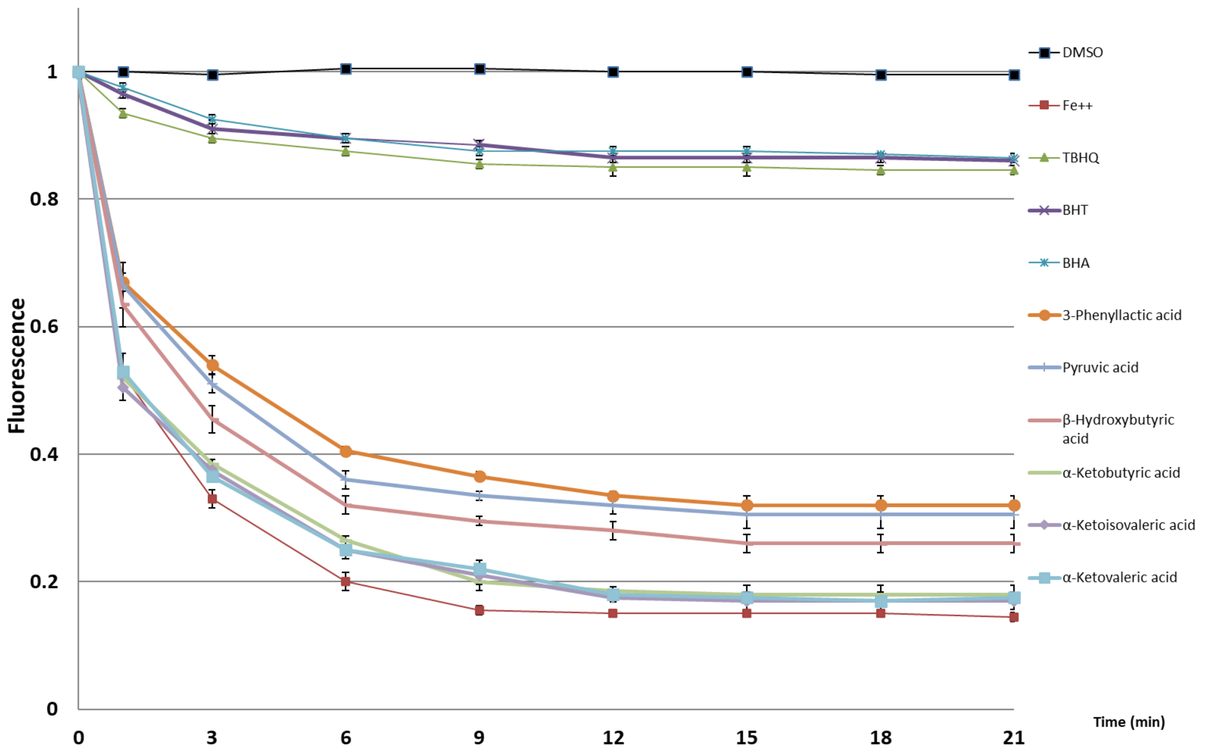

Figure V Inhibition of lipid peroxidation (LPO) by organic acids α-ketovaleric acid, α-ketoisovaleric acid, α-ketobutyric acid, β-hydroxybutyric acid, pyruvic acid and 3-phenyllactic acid at 25 µg/mL concentration. Commercial antioxidants BHA, BHT and TBHQ used as positive controls at 1.8, 2.2 and 1.6 µg/mL, respectively. The varying concentrations used were to yield comparable activity profiles between 0-100 % by test compounds and positive controls alike. Vertical bars represent the standard deviation of each data point (n=2). The percent inhibition was calculated with respect to solvent control dimethyl sulfoxide.
